# The value of Bayesian predictive projection for variable selection: an example of selecting lifestyle predictors of young adult well-being

**DOI:** 10.1186/s12889-021-10690-3

**Published:** 2021-04-09

**Authors:** A. Bartonicek, S. R. Wickham, N. Pat, T. S. Conner

**Affiliations:** Department of Psychology, University of Otago, Dunedin, New Zealand

**Keywords:** Variable selection, Prediction, Inference, Psychological well-being, Young adults, Health habits, Health behaviors, Sleep, Diet, Exercise

## Abstract

**Background:**

Variable selection is an important issue in many fields such as public health and psychology. Researchers often gather data on many variables of interest and then are faced with two challenging goals: building an accurate model with few predictors, and making probabilistic statements (inference) about this model. Unfortunately, it is currently difficult to attain these goals with the two most popular methods for variable selection methods: stepwise selection and LASSO. The aim of the present study was to demonstrate the use predictive projection feature selection – a novel Bayesian variable selection method that delivers both predictive power and inference. We apply predictive projection to a sample of New Zealand young adults, use it to build a compact model for predicting well-being, and compare it to other variable selection methods.

**Methods:**

The sample consisted of 791 young adults (ages 18 to 25, 71.7% female) living in Dunedin, New Zealand who had taken part in the Daily Life Study in 2013–2014. Participants completed a 13-day online daily diary assessment of their well-being and a range of lifestyle variables (e.g., sleep, physical activity, diet variables). The participants’ diary data was averaged across days and analyzed cross-sectionally to identify predictors of average flourishing. Predictive projection was used to select as few predictors as necessary to approximate the predictive accuracy of a reference model with all 28 predictors. Predictive projection was also compared to other variable selection methods, including stepwise selection and LASSO.

**Results:**

Three predictors were sufficient to approximate the predictions of the reference model: higher sleep quality, less trouble concentrating, and more servings of fruit. The performance of the projected submodel generalized well. Compared to other variable selection methods, predictive projection produced models with either matching or slightly worse performance; however, this performance was achieved with much fewer predictors.

**Conclusion:**

Predictive projection was used to efficiently arrive at a compact model with good predictive accuracy. The predictors selected into the submodel – felt refreshed after waking up, had less trouble concentrating, and ate more servings of fruit – were all theoretically meaningful. Our findings showcase the utility of predictive projection in a practical variable selection problem.

**Supplementary Information:**

The online version contains supplementary material available at 10.1186/s12889-021-10690-3.

## Background

*Variable selection* is an important topic in public health, well-being, and other fields. Researchers often collect data on large numbers of variables (predictors) that are plausibly related to the outcome of interest, and then try to find an optimal subset of the predictors that can maximally predict the outcome measure [26, 28, 54]. For example, in health and well-being research, researchers may collect data on many demographic, lifestyle, and psychological variables, and then aim to build a compact model with fewer variables that can accurately predict the participants’ self-reported well-being. There are important reasons for why researchers might choose to prefer a more compact model - simpler models align with the core scientific principle of parsimony, and are by definition easier to interpret [26]. Importantly, the simplified model should still be able to fulfil two important functions. First, its performance should be close to that of the original model, and should generalize to new, out-of-sample data. Second, researchers should be able make probabilistic statements about it – how uncertain is the selection, how variable is the model’s performance, and, perhaps most importantly, how strong and reliable are the relationships between the selected predictors and the outcome [69]. Ideally, variable selection should produce models that simultaneously provide both of these important functions: predictive power and inference.

Additionally, variable selection is often necessary because the exact set of predictors to be included in the model is simply not known. This goes against the core assumption in traditional regression modelling that all important predictors should be known a-priori. However, across many fields, the theory is rarely as strong as to determine the set of predictors to include in the model exactly, and there are choices to be made [26, 36, 74]. Under favourable circumstances, variable selection can be done by fitting all possible models and keeping the best one. This is possible when the number of candidate predictors is small; however, with many predictors, this best-subset approach quickly becomes computationally expensive – with k predictors, there are 2^k^ models that need to be fitted and evaluated (e.g. 1024 models with 10 predictors, 1,048,576 models with 20 predictors [26, 35];). Specialized variable selection methods are thus necessary to reduce the computational burden and make the task of finding the optimal subset of predictors possible.

Currently, there are two widely used variable selection methods: stepwise selection [16], and the least absolute shrinkage and selection operator [71]. Stepwise selection is the more traditional method of the two, and is characterized by building up the model step-by-step, each time either adding or subtracting a predictor based on some pre-specified criterion (typically either *p*-values or information criteria such as the AIC or BIC [80];). LASSO is a newer, popular machine learning method, in which models are typically fitted via the efficient least-angle regression (LARS) algorithm [15]. LARS resembles forward stepwise regression except that instead of adding or removing predictors wholesale, the predictor slopes are continuously increased towards their least-squares solution. A key additional feature of LASSO is that the method places a constraint or a limited “budget” on the sum of the absolute predictor slopes [72], via the penalization parameter lambda. The constraint on the sum of the predictor slopes produces shrinkage – the slopes are shrunk towards zero when compared to classical least squares estimates, and this in turn leads to better out-of-sample predictive performance [87]. The optimal value of lambda is typically tuned through k-fold cross-validation – a procedure in which the data are split into k parts, and then k - 1 parts are repeatedly are used for model fitting, with the leftover part always used for model evaluation [3, 25, 44].

Unfortunately, at the present moment, it is difficult to obtain predictive power and inference simultaneously with stepwise selection and LASSO. Stepwise selection has major issues when it comes to predictive power, since it suffers from *overfitting* – it is liable to select predictors that fit to pure noise in the data, and shows poor out-of-sample predictive performance as a result [17, 35, 70, 80]. Overfitting is a well-known problem in machine learning: extra parameters always make a model more flexible and allow it to fit the data better, and so even completely irrelevant predictors can ostensibly improve a model’s fit [24, 27]. Overfit models seem to explain the data at hand well (as indicated by good performance metrics, such as high R^2^, low RMSE). However, this performance does not generalize, and so overfit models end up performing poorly when faced with new data. Importantly, some authors have argued that overfitting may be one of the less-known causes of replication crisis, in that some research findings may fail to replicate not because of any ill-will of the researchers but because they are supported by fragile trends that are idiosyncratic to the training data [86]. Stepwise selection is liable to overfitting, especially when *p*-values are used as the criterion for adding or subtracting predictors [17, 35, 70, 80]. In contrast, LASSO models are specifically designed to counteract overfitting. By penalizing the sum of the absolute slopes, LASSO models produce more restrained predictions that generalize well to new data. However, both stepwise regression and LASSO are lacking when it comes to inference. In stepwise selection, classical inferential tools such as *p*-values and confidence intervals are sometimes used to summarize the final model. Yet, these *p*-values and confidence intervals are invalid because they ignore the selection procedure [17, 45, 66, 67, 80]. In LASSO, classical inferential tools were simply not available for a long time, and while some methods for inference with have been developed recently, they are fairly involved, and the area is still undergoing development [4, 69].

*Predictive projection feature selection* is a novel method for variable selection within the Bayesian framework [56] that delivers both predictive performance and inference. The method consists of two steps. At the first step, a flexible reference model is fitted using all available predictors. At the second step, smaller submodels are fitted to approximate the reference model’s predictions, using projection. Finally, the smallest submodel which makes predictions “similar enough” to those of the reference model is selected (i.e., the submodel with estimated performance matching the reference model within some uncertainty bound, such as one standard error [56];). To avoid overfitting, the submodels are compared to the reference model on cross-validated prediction accuracy, via the efficient Bayesian approximation of leave-one-out cross-validation: Pareto-smoothed importance sampling leave-one-out cross-validation (PSIS-LOO [75];). There are three key advantages to predictive projection feature selection: it selects a parsimonious model with good predictive accuracy, is robust to overfitting, and produces a valid posterior distribution that can be used for inference just like in any other Bayesian posterior [56]. That is, the posterior distribution of the projected submodel can be used to make statements about the uncertainty in the model’s performance and its parameters, for example by summarizing these via credible intervals.

### Applying Bayesian predictive projection: predictors of well-being

In the present paper, we applied predictive projection feature selection on a dataset related to well-being. The issue of variable selection arises frequently in well-being research. Determining which factors are associated with greater well-being is important because there is robust evidence showing that psychological well-being is linked with slew of positive outcomes including better physical health and greater longevity [12, 39–42, 62]. Past research has identified predictors of well-being on many levels, including socioeconomic trends and policies, such as population density, income inequality, and strength of social welfare systems [13, 31, 47, 55, 68], community and family factors, such as childhood socioeconomic status (SES [58];). Particularly interesting to well-being researchers are individual-level lifestyle factors such as sleep, diet, and exercise, as these can be modified and can thus be target for intervention studies. Among these health habits, sleep quality has been consistently shown as one of the strongest predictors of well-being [38, 57, 61, 64, 78, 82]. Diet quality has also been linked with well-being – specifically, fruit and vegetable consumption has been shown to be associated with greater well-being in observational [1, 5, 10, 33, 52, 59, 61], prospective [32], micro-longitudinal or daily diary [32, 48, 77, 79] and experimental studies [9, 34]. Conversely, poor diet as indicated by soft drink and fast-food consumption has also been linked to lower well-being [29, 52, 61]. Finally, exercise has been linked to greater well-being [13, 21, 23, 61]. The question arises though, which of these lifestyle factors, or possibly other demographically related factors (such as BMI, SES, age, gender, etc.) offers the most compact and efficient model predicting well-being?

In the present study, we demonstrate the use of predictive projection feature selection by constructing a compact model for predicting well-being, using data from a sample of 791 young adults. The participants took part in the 2013 and 2014 waves of the Daily Life Study, a daily diary study of the health and well-being of young adults in Dunedin, New Zealand. Well-being was surveyed every day for 13 days using the 8-item Flourishing Scale [14] adapted for daily measurement. We first fitted a Bayesian multiple regression reference model, predicting average daily flourishing cross-sectionally from 28 candidate well-being predictors. The candidate predictors included demographic and background variables (e.g., age, gender, childhood SES, BMI), and an extensive range of lifestyle variables assessed in the daily diary related to stress (stress, most stressful event of the day), somatic symptoms (e.g. tiredness, lack of ability to concentrate), diet-related variables (e.g. fruit and vegetable consumption), and health habit variables (e.g. sleep quality/quantity, exercise). After fitting the reference model, we used predictive projection to find a smallest possible submodel that would predict average daily flourishing almost as well as the reference model. Finally, we compared predictive projection to stepwise selection and LASSO.

## Method

### Software and packages

Throughout our entire workflow, we used the programming language R, version 3.6.3 [63]. For general-purpose Bayesian modelling, we used the brms package [7], which provides an interface to the state-of-the-art Bayesian statistical programming language Stan [8]. For predictive projection feature selection, we used the projpred package [56]. For additional packages used, see Appendix A.

### Participants and procedure

Participants came from the second two waves (2013/2014) of the Daily Life Study, a daily diary study assessing the psychological well-being and daily health habits in a large sample of young adults living in Dunedin, New Zealand (total *n* = 821, 71.7% female). The Daily Life Study was run across four years from 2011 to 2014; however, we selected participants from the 2013/2014 waves only because the information that was collected changed over time and fewer variables of interest were collected during the 2011/2012 waves (for example, no diet-related variables). Most of the participants were University of Otago students, and all were between 17 and 25 years old (m = 19.73, sd = 1.73). About half of the participants (57.2%) were recruited via psychology courses, the rest were recruited via physical advertising (22.1%) and online recruitment (20.7%). The over-representation of females in our sample may have been due to the over-representation in the target population (young University students in New Zealand), as well as the characteristics of the study (micro-longitudinal study focused on health and well-being). The majority of participants were New Zealand European/Pākehā (77.5%), followed by Asian (10.6%), and Māori/Pacifica participants (5.4%). Participants of all other ethnicities made up 6.4% of the sample and were aggregated into one category. The participants first completed an initial survey on demographics, and then starting the next day were tracked across 13 days via an online daily diary completed each night between 3 and 8 pm. Participants also attended a single clinic visit during which height and weight was recorded and used to compute BMI.

### Measures, data cleaning, and preprocessing

A list of measures with reliabilities and descriptive statistics can be found in the Supplementary Materials. We used the following demographic variables (*n* = 5) from the initial survey: age, gender, ethnicity, self-reported childhood socioeconomic status (SES [22];), and a measure of body mass index (BMI). From the daily survey, we took the daily flourishing scale items [14], as well as all stress self-assessment, somatic self-assessment, diet-related, and health habit variables (total *n* = 22) that were measured for the entirety of 2013–2014 and could be theoretically linked with flourishing (see Supplementary Materials for detailed description of all survey items and response options). While most of the 22 variables assessed the participants’ state on the day of reporting, some of the variables also assessed the participants’ state on the night before. Of the 22 variables, the stress self-assessment variables (*n* = 2) were: felt stressed today, most stressful event today. The somatic self-assessment variables (*n* = 5) were: felt tired today, felt rundown today, felt cold or flu today, had hangover today, and had trouble concentrating today. The diet-related (*n* = 11) variables were: servings of fruit today, servings of vegetables today, servings of sweets today, servings of soft drink today, servings of chips today, servings of fruit last night, servings of vegetables last night, servings of sweets last night, servings of soft drink last night, servings of chips last night, and standard drinks of alcohol last night. The health habits (*n* = 4) were: hours slept last night (sleep quantity), felt refreshed after waking up today (sleep quality), minutes physically active today, and minutes spent in nature today. Most variables were reported on a 5-point Likert scale, (0 = not at all, 1 = a little, 2 = somewhat, 3 = moderately, 4 = very). Drinking alcohol, sleep quantity, time spent in nature, physical activity, and the diet related variables were freely reported in the specified units (standard drinks, hours, and minutes, respectively, and servings).

**Fig. 1:**
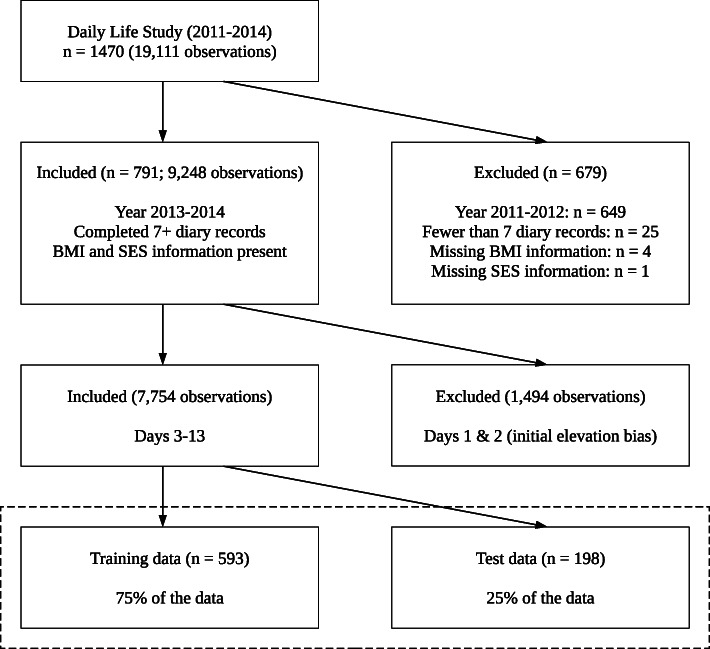
Flow diagram of the data selection procedure. Not applicable

Figure 1 presents a flow diagram of data cleaning. We excluded those participants who had provided fewer than 7 diary records (out of 13 possible) across the course of the study (*n* = 25). Across all daily variables, we dropped the first two days of observations to account for initial elevation bias – the tendency of participants to over-report symptoms in the beginning of longitudinal studies [65]. To make sure that excluding the first two days did not bias our results, we ran a sensitivity analysis (see Supplementary Materials). Additionally, there were 0.5% of missing values across all 29 daily variables (8 flourishing items + 22 lifestyle habits). All variables except for minutes spent in nature today were missing less than 1.5% of all values. The minutes spent in nature variable was missing 4.35% of all values, and there were several participants for whom all or majority (> 50%) of values were missing. Because of the number of missing values, we decided to drop the variable, we again ran sensitivity analysis to show that its exclusion did not affect the selection (see Supplementary Materials). After excluding the time spent in nature variable, the daily (*n* = 21) and demographic (*n* = 5) made up a total of 26 variables. Because the ethnicity variable had four levels, this added two additional dummy predictors, and so there were in the end 28 predictors, including dummy variables. We did not impute 0.4% of missing values that were left after excluding the time spent nature variable, as the values for each variable would be averaged across days for each participant. As for demographic variables, there were four participants missing their BMI information, and one participant missing two out of the three of the childhood SES items. These five participants were dropped from the analysis. Thus, the final sample consisted of 791 participants. Dropping the 30 participants from our had little effect on the demographic variables. The mean age in the final sample was 19.72 (sd = 1.73), and a majority of participants were female (72.3%). Most of the participants retained for analysis were New Zealand European/Pākehā (77.5%), followed by Asian (10.8%), Māori/Pacifica (5.4%) participants, with the rest making up 6.3% of the sample.

The eight flourishing scale items were averaged into one daily flourishing variable. Following that, all variables from the daily survey were averaged across days (including the newly created daily flourishing variable). Additionally, childhood SES was averaged across items as well. Finally, all continuous variables were centred and scaled to 1-unit standard deviation.

### Statistical analyses and modeling

Prior to fitting any models, we randomly split our data into a training set (75%; *n* = 593) and test set (25%, *n* = 198). The training data was used to fit all models. The test data was used to validate the models’ predictions on held-out data. All relevant statistics are reported for both the training and test data.

We first fitted a Bayesian multiple regression as the reference model, predicting average daily flourishing from all 28 candidate well-being predictors (21 daily predictors, 5 demographic predictors, with 2 additional dummy predictors for ethnicity). We used weakly informative normal priors for the predictor slopes and the intercept (normal: mean = 0, sd = 1). For model standard deviation, we also used a weakly informative prior (half-normal: mean = 0, sd = 1). As for sampling, we ran four chains of 4000 iterations, with 2000 iterations of warm-up and 2000 iterations of sampling each. The chains were run in parallel to speed up convergence.

After fitting the reference model, we used predictive projection feature selection to fit a submodel with fewer predictors that would give similar predictions to the full model. To implement predictive projection, we used the projpred package [56]. The variables were entered into the submodels using L1 search (projpred default for > 20 variables) and the submodels’ predictive performance was evaluated and compared using expected log predictive density (ELPD) obtained through PSIS-LOO cv (Pareto-smoothed importance sampling leave-one-out cross-validation; [75]). For the optimal submodel, we chose the smallest submodel that had ELPD within 1 standard error of the reference model (1SE-submodel; projpred default). That is, using this rule, we chose the smallest submodel which was expected to perform the same as/outperform the reference model with at least 16% probability (and perform worse with 84% probability).

Finally, we compared the predictive performance of predictive projection feature selection to five other models: the original reference model (Bayesian multiple regression), a frequentist multiple regression model with all predictors, frequentist stepwise selection model using *p*-values, frequentist stepwise selection model using Akaike Information criterion [2], LASSO with minimum cross-validated RMSE (min-LASSO), and LASSO with cross-validated RMSE within 1 SE of the minimum (1SE-LASSO). R does not provide a default function for stepwise selection using *p*-values so we used a publicly available R code that implements an SPSS-like stepwise selection with p-values [51]. Besides the projected 1SE-submodel described above, we also included in the comparison a projected submodel with cross-validated predictive performance set to match reference model, that is, the smallest possible projection submodel for which there was at least 50% probability that it would perform as well as/better than the reference model. In total, eight models were compared. To evaluate the performance of all models, we used root mean squared error (RMSE) and Bayesian *R*^2^ = $\frac{Var_{fit}}{Var_{fit}+{Var}_{res}}$. Bayesian *R*^2^ is a generalization of classical *R*^2^, with the advantage that it can incorporate posterior uncertainty and remains bounded below 1 (with 0 indicating no predictive power and 1 indicating perfect predictive power), even in the presence of strong priors and weak data [19]. Here we used Bayesian *R*^2^ just as a convenient summary of predictive performance that we could compare: a) between training and test data, and b) across models on test data only.

## Results

The reference model converged well, with no divergent transitions and good $\hat{R}$ values (all ≈ 1), indicating that the chains mixed well [20]. All parameters had a good effective sample size (all > 5000). The model passed simple posterior predictive checks and visual checks of residuals did not reveal any heteroscedasticity or gross non-linear trends (see Supplementary Materials). There were few outliers with very low average daily flourishing in the data; however, when we evaluated the model via PSIS-LOO cv, we found no evidence of these observations having a disproportionate influence on the model fit, as indicated by satisfactory Pareto-k values (all “good”, k < 0.5; see Supplementary Materials). On the training data, the reference model had a Bayesian *R*^2^ of 0.359 (0.308–0.405, 95% credible interval; CI) and a model standard deviation/RMSE of 0.821 (0.774–0.870 95% CI), indicating a moderately good predictive performance.

**Fig. 2:**
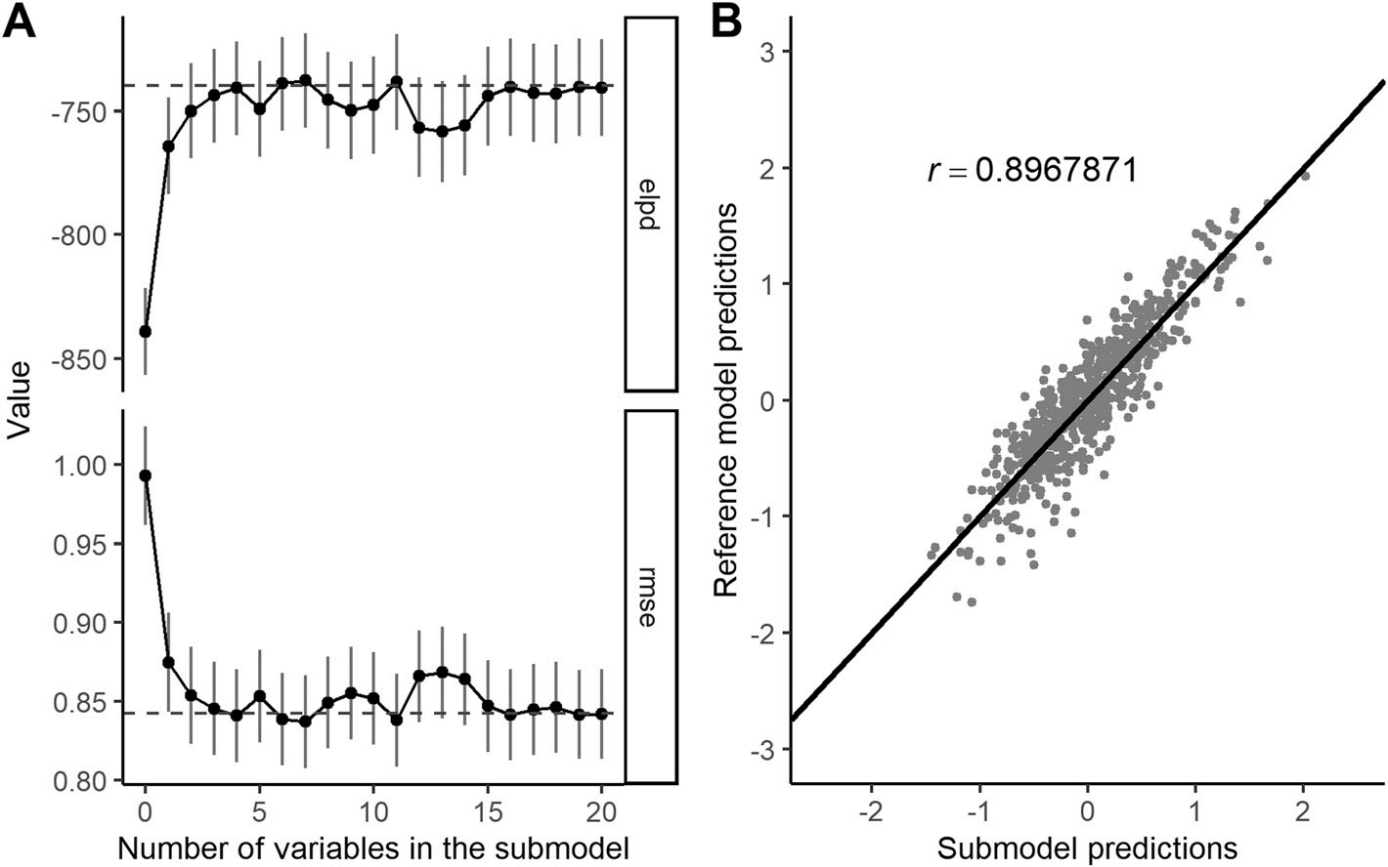
Predictive projection feature selection trajectory and scatterplot of reference model’s vs. submodel’s prediction. **a**) Change in ELPD/decrease in RMSE as more predictors entered the submodel. **b**) Average daily flourishing predicted by the submodel (3 predictors) vs. the average daily flourishing predicted by the reference model (28 predictors; both predicting training data)

**Fig. 3:**
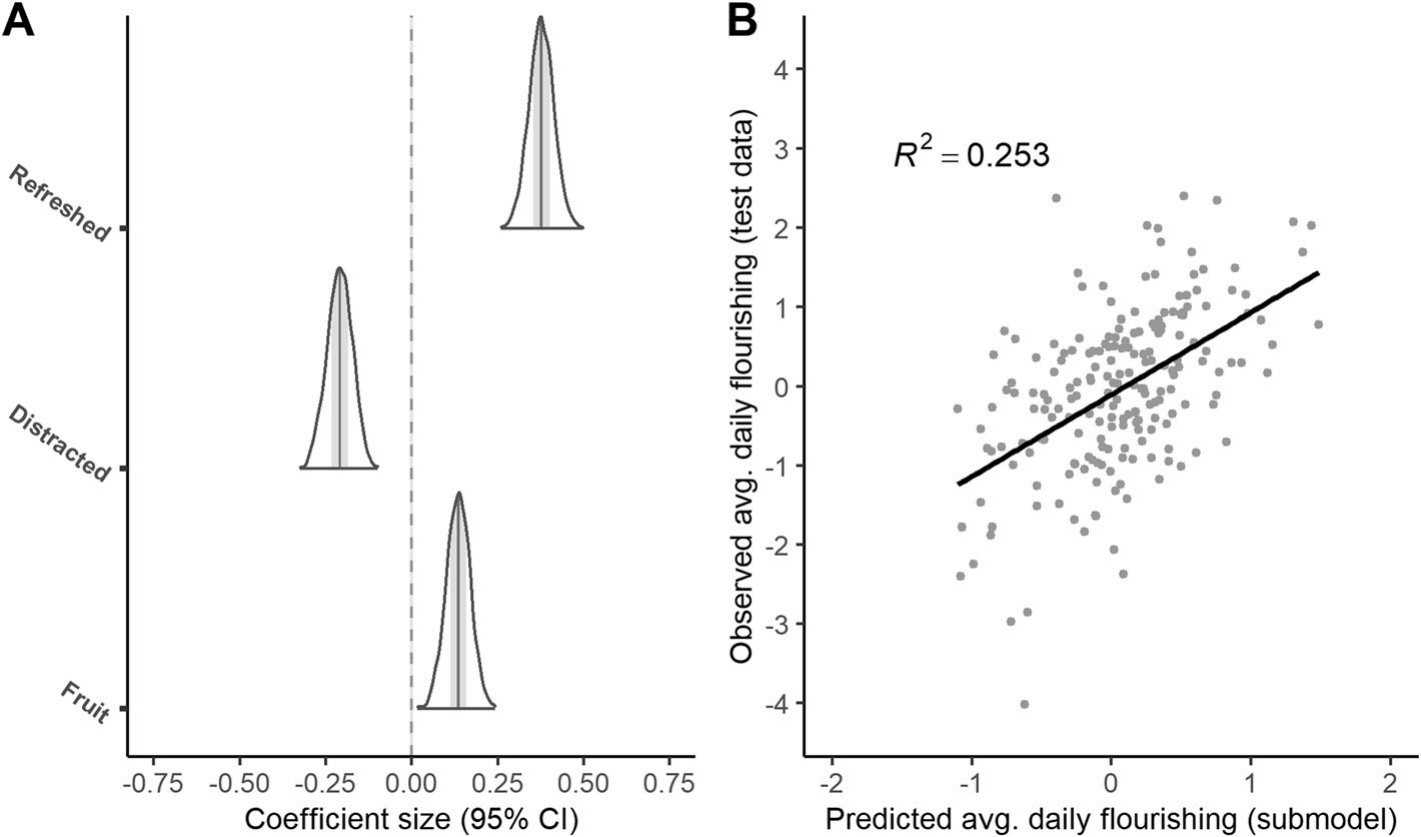
Credible intervals for predictors in the submodel and scatterplot of submodel’s predictions vs. observed values. Marginal posterior distributions of predictors selected for the submodel (in order: felt refreshed after waking up today, had trouble concentrating today, servings of fruit today). **b**) Average daily flourishing predicted by the submodel vs. observed daily flourishing (unseen test data), with overlaid least squares fit

Using the predictive projection, we found that the submodel based on the 1SE rule, which included only 3 predictors, made predictions that were similar enough to those of the reference model with all 28 predictors. The sensibility of the 1SE rule was confirmed with a visual check of the feature selection trajectory, which showed that predictive performance did seem to stop improving after 3 predictors (see Fig. 2a). On the training data, the 1SE-submodel had a Bayesian *R*^2^ of 0.267 (0.213–0.323, 95% CI) and a model standard deviation/RMSE of 0.878 (0.826–0.931 95% CI). The 1SE-submodel’s predictions were strongly correlated with the reference model’s predictions (Pearson r = 0.897, see Fig. 2b). The three predictors in the optimal submodel, in order as they were entered into the submodel, were: felt refreshed after waking up today (0.377, 0.306–0.453 95% CI), had trouble concentrating today (− 0.211, − 0.281 – − 0.138 95% CI), and servings of fruit today (0.132, 0.065–0.200 95% CI; see Fig. 3a). We tested the submodel’s performance on the independent test data and it performed well, with an observed Bayesian *R*^2^ of 0.253 and an observed RMSE of 0.884 (see Fig. 3b).

The results of the comparison of variable selection methods are shown in Table 1. The overall trend was that the largest models had the best predictive accuracy, as assessed by test data *R*^2^ and RMSE: frequentist multiple regression reference model (28 predictors; R^2^: 0.332, RMSE: 0.858), reference model (28 predictors; R^2^: 0.331, RMSE: 0.858) and the min-LASSO (23 predictors; R^2^: 0.283, RMSE: 0.857). Based on Bayesian R^2^ alone, the models that performed the best were: frequentist multiple regression (0.332), the reference model (0.331), AIC-stepwise (0.315), matched-submodel (0.284), min-LASSO (0.283), p-stepwise (0.275), 1SE-submodel (0.253), 1SE-LASSO (0.139). Based on RMSE, the models that performed the best were: the reference model (0.858), the frequentist multiple regression (0.858), min-LASSO (0.857), matched-submodel, p-stepwise (0.864), p-stepwise (0.871), AIC-stepwise (0.871), 1SE-submodel (0.883), 1SE-LASSO (0.893). In terms of the numbers of selected predictors, predictive projection produced the simplest models, with 1SE-submodel containing only 3 predictors and the matched projection submodel containing 6 predictors, fewer than any of the other methods apart from 1SE-LASSO (4 predictors).

**Table 1:** Comparison of variable selection methods

Model	R2	RMSE	# of selected predictors	Selected predictors	
Reference model	0.331	0.858	(28)	–	
Freq. multiple regression	0.332	0.858	(28)	–	
Projected submodel (1 SE)	0.253	0.883	3	Felt refreshed after waking up today, had trouble concentrating today, servings of fruit today	
Projected submodel (matched)	0.284	0.864	6	Felt refreshed after waking up today, had trouble concentrating today, servings of fruit today, servings of soft drink last night, servings of vegetables today, gender: female	
Stepwise selection (AIC)	0.315	0.872	10	Felt refreshed after waking up today, ethnicity: asian, had trouble concentrating today, gender: female, servings of soft drink last night, servings of sweets today, servings of sweets last night, felt tired today, servings of fruit today, bmi	
Stepwise selection (p-values)	0.275	0.871	8	Felt refreshed after waking up today, had trouble concentrating today, gender: female, servings of sweets today, felt tired today, servings of sweets last night, servings of fruit today, servings of soft drink last night	
LASSO (1 SE)	0.139	0.897	4	Felt refreshed after waking up today, had trouble concentrating today, servings of fruit today, servings of soft drink last night	
LASSO (min.)	0.283	0.857	23	–	

Summary statistics of model selection strategies, showing test data RMSE and Bayesian R^2^, number of selected predictors, and the names of the significant predictors (where 10 or fewer predictors were selected, ranked by absolute slope size)

## Discussion

Using predictive projection feature selection, we found that only three of the 28 candidate well-being predictors were sufficient to approximate the predictions of a large reference model with all 28 predictors. Specifically, participants who reported feeling more refreshed after waking up, having less trouble concentrating, and eating more servings of fruit scored highest in their average daily flourishing. We tested the optimal 3-predictor submodel on test data and found that its predictive performance generalized well to new data, with performance measured by RMSE and Bayesian *R*^2^ only slightly worse than on the training data and well-within the training data uncertainty bounds. Lastly, we also found that, when comparing predictive projection to other variable selection methods, larger models generally tended to have better predictive accuracy on test data. However, predictive projection tended to produce smaller models, and the projection submodel matched to the reference model performed better than stepwise selection based on *p*-values, with fewer predictors.

The projected 1SE-submodel had somewhat worse predictive accuracy on test data than most of the other variable selection methods, as measured by RMSE and Bayesian R^2^, yet it achieved this performance with much fewer selected predictors than the other methods. In fact, the 1SE-submodel had better test data performance (as indicated by both RMSE and *R*^2^) than the second smallest model – 1SE-LASSO – which included an additional predictor. The other models included at least twice as many predictors as the projected 1SE-submodel or more. Interestingly, 1SE-LASSO had a particularly low Bayesian R^2^. However, this was due to strong shrinkage – the model predicted only a narrow range of outcomes which lead to very small variance in predictions (the numerator in Bayesian R^2^, see Supplementary Materials). The reason why large models performed the best in our study is most likely because we had used a relatively large training sample (*n* = 593). Overfitting is less of a concern the when ratio of training observations to the number of predictors is large [50], as was the case in our study. The more regularizing methods, namely the 1SE predictive projection and 1SE LASSO, may have performed comparatively better, if the training sample had been smaller. Still, predictive projection produced a well-performing model, considering it had contained only 3 predictors.

It is also important to highlight that while the two stepwise models and the min-LASSO model had higher test-data predictive accuracy than the projected 1SE-submodel, none of these models can be readily used for inference. The stepwise models cannot be used for inference because the *p*-values and standard errors from these models are not adjusted for the selection and as such do not control for type-I error [17, 66]. As for LASSO, the tools for inference with these models are still undergoing development [4, 69]. The projected submodels, on the other hand, have a valid posterior distribution [56] and thus can be used for inference.

The fact that the submodel with only three predictors was sufficient to predict almost as much variation in average daily flourishing as the reference model with all 28 predictors does not suggest that the left out 25 predictors have no relationship with flourishing. Instead, our results suggest that the three selected predictors are the strongest predictors of average daily flourishing, at least among groups of correlated predictors. For example, inasmuch as diet is related to flourishing, our results suggest that fruit consumption may be the strongest indicator of good diet and flourishing, as indicated by the fact that it was the earliest from the group of diet-related predictors in the feature selection trajectory, and was the only diet-related predictor that was present in the optimal submodel. Likewise, while there were several correlated candidate predictors related to fatigue and somatic issues, the fact that having (less) trouble concentrating was the first and only predictor from this group selected into the optimal submodel suggests that it may be the strongest somatic predictor of well-being.

The three predictors selected into the projected submodel are all theoretically meaningful within the field of well-being research. The first predictor selected into the submodel was sleep quality – how refreshed participants felt after waking up, on their average day. Sleep quality has been consistently shown to be one of the strongest predictors of well-being, especially in young adults, with poor sleep quality being strongly linked to poor mental health outcomes including symptoms of depression ([57, 64, 78]; S.-R [82, 84].). Additionally, while sleep quality has often been shown to be an important predictor of well-being, sleep quantity has not [57, 78], and this is congruent with our results – while sleep quality was entered into the submodels early along the feature selection trajectory, sleep quantity was only entered long after any improvement in predictive accuracy was shown, indicating a lack of predictive power. The second predictor selected into the submodel was how much trouble concentrating a participant had on their average day. This results is also meaningful – having trouble concentrating is one of the key symptoms of the major depressive disorder (MDD), and is often assessed by diagnostic scales, such as the popular CES-D scale [43] and the DSSS scale [30]. Finally, the third and the last predictor entered into the submodel was daytime fruit consumption. As discussed above, the fact that daytime fruit consumption was the first diet-related predictor to be selected in the feature selection trajectory and the only diet-related predictor that made it into the submodel suggests that it may be one of the strongest indicators of diet quality, as it relates to flourishing. Fruit and vegetable consumption has been previously shown to predict psychological well-being and flourishing [5, 9, 10, 29, 53, 60]. There were other diet related predictors entered into the submodel early along the feature selection trajectory, namely (lower) night-time soft drink consumption and daytime vegetable consumption as the 4th and 5th predictors, respectively. However, based on the 1 SE rule, fruit consumption only was sufficient to approximate the reference model’s predictions, suggesting that fruit consumption may contain enough information about the quality of one’s diet to make other diet-related predictors redundant. Additionally, there is evidence that raw fruit and vegetables are stronger predictors of well-being than cooked fruit and vegetables [6, 82], and since fruit is more often eaten raw, general fruit consumption may be a stronger indicator of good diet than vegetable consumption. Be it as it may, sleep quality, having trouble concentrating, and fruit consumption are all meaningful predictors within the well-being literature.

Our study has several limitations. First, the predictive performance of predictive projection and the other methods was evaluated on only one independent test set. As such, the RMSE and R^2^ values we obtained in our study may be subject to sampling variation. While this is an issue, our main goal was to demonstrate the use of predictive projection and show that it is a viable alternative to the other methods – not to firmly prove that it has superior predictive performance. Even if other methods predict better, they are still lacking when it comes to inference. Future studies may use simulation to compare predictive performance of predictive projection, LASSO, and stepwise selection, under different conditions such as sample size and distribution of effect sizes. Second, as was mentioned earlier, the fact that only three variables were selected into the optimal submodel does not mean that the other predictors are not related to well-being. Therefore, we cannot conclude that the three predictors are the only predictors of flourishing, only that they are sufficient to predict it with high degree of accuracy, similar to the model with all 28 predictors. Third, given that our data is observational, we cannot make causal claims about the predictors’ influence. When we say that a predictor predicts higher average daily flourishing, we mean that participants with higher values in the predictor tend to report higher average daily flourishing. In concrete terms, poor sleep, lack of ability to concentrate, and poor diet, may not cause low well-being, but instead may be just indicators or even a product of low well-being. While the lack of ability to make directional causal claims is certainly a limitation, analyses of observational data are important and necessary to identify possible targets of interventions, to be investigated in follow up research. Fourth, our sample was relatively homogenous, consisting of young, mostly female, mostly Caucasian, college-age adults (age-range 17–25 years) from New Zealand. Thus, the findings are not necessarily likely to generalize to other populations. For example, young adults tend to be at an increased risk for poor sleep quality [49], and so the strong association between sleep quality and well-being in our sample may be tied to the demographic characteristics of our sample. Ultimately, the only concrete evidence for generalizability is direct replication [44, 85]. Fifth, as our outcome measure, we used the Flourishing Scale [14], and while this is a popular measure of well-being, it is by far not the only one. There is an extensive amount of theoretical work comparing the different ways of measuring well-being that is outside the scope of the present article; however, it may be interesting to see how much the results from predictive projection generalize to other well-being scales. Finally, while our data comes from a micro-longitudinal daily diary study, we only analysed our data cross-sectionally. There are several reasons for why we did not analyse the within-person patterns over time. First off, predictive projection feature selection is not yet implemented for mixed effects models [56] and so at the present moment we do not have the ability to build within-participants models in the same way as we did across participants. Second, while we could use the three predictors from the submodel to fit a within-participant mixed effects model, there is no guarantee that the three strongest cross-sectional predictors of flourishing across participants will also be the strongest predictors of flourishing within-participants – in fact, this is unlikely (Simpson’s paradox; see [76]). As such, we believe that repeated measures analyses using mixed effects models are outside of the scope of this article.

## Conclusion

We have demonstrated that how predictive projection feature selection can be used to build compact models with good predictive power that can also be used for inference. Specifically, we were able to accurately predict average daily flourishing across young adults in our sample with a model that used information from just three predictors: how refreshed the participants felt after waking up, how much trouble they had concentrating, and how many servings of fruit they ate on their average day. That is, using a model with only three predictors, we were able to obtain a predictive accuracy that was fairly comparable to that of a large model with all 28 predictors. Compared with the other variable selection methods, predictive projection performed adequately and produced much more parsimonious models. Our final submodel was congruent with established findings in the well-being literature, in that sleep quality was more strongly associated with better well-being than sleep quantity, having trouble concentrating was related to lower well-being, and having good dietary habits (as indicated by fruit consumption) was related to higher well-being. Finally, variable selection is a common issue that arises frequently across many fields. The currently popular methods for variable selection (stepwise selection, LASSO) do not produce models that simultaneously provide good out-of-sample prediction and valid, straightforward inference. Predictive projection is both robust to overfitting and provides valid Bayesian inference, but has not yet been widely adopted. We believe that predictive projection is a method with great utility and we hope the present article shows how it can be used to solve practical variable selection problems.

### Supplementary Information

**Additional file 1.** Supplementary materials.

## Data Availability

The Daily Life Study dataset contains sensitive demographic information that could potentially be used to identify individual participants. De-identified dataset will be made available from the corresponding author upon reasonable request. All R code used for the analysis is available as part of the Supplementary Materials.

## Appendix

For data wrangling and visualization, we used the tidyverse package [81]. For general-purpose Bayesian modelling, we used the brms [7] – a package that provides an accessible interface to the state-of-the-art Bayesian statistical programming language Stan [8]. For predictive projection feature selection, we used the projpred package [56]. For Pareto-smoothed importance sampling leave-one-out cross-validation (PSIS-LOO), we used the loo package [75], and for model checking and visualization, we used the bayesplot package [18]. Additional packages used include pander [11], naniar [73], cowplot [83], ggcorrplot [37], and labelled [46].

## Abbreviations

BMI: Body mass index
CI: Credible interval
CES-D: Center for Epidemiological Studies - Depression scale
DSSS: Depression and somatic symptoms scale
ELPD: Expected log-predictive density
PSIS-LOO cv: Pareto-smoothed importance sampling leave-one-out cross-validation
RMSE: Root-mean-squared error
SE: Standard error
SES: Socioeconomic status

## References

[1] Adams, T. B., & Colner, W. (2008). The association of multiple risk factors with fruit and vegetable intake among a nationwide sample of college students. J Am Coll Heal, 56(4), 455–461. https://doi.org/10.3200/JACH.56.44.455-464. 10.3200/JACH.56.44.455-464 18316291

[2] Akaike H (1974). A new look at the statistical model identification. IEEE Trans Autom Control.

[3] Arlot, S., & Celisse, A. (2010). A survey of cross-validation procedures for model selection. Statistics Surveys, 4, 40–79. https://doi.org/10.1214/09-SS054, none.

[4] Benjamini, Y. (2010). Simultaneous and selective inference: current successes and future challenges. Biom J, 52(6), 708–721. https://doi.org/10.1002/bimj.200900299. 10.1002/bimj.200900299 21154895

[5] Blanchflower, D. G., Oswald, A. J., & Stewart-Brown, S. (2013). Is psychological well-being linked to the consumption of fruit and vegetables? Soc Indic Res, 114(3), 785–801. https://doi.org/10.1007/s11205-012-0173-y.

[6] Brookie, K. L., Best, G. I., & Conner, T. S. (2018). Intake of raw fruits and vegetables is associated with better mental health than intake of processed fruits and vegetables. Front Psychol, 9(APR), 1–14. https://doi.org/10.3389/fpsyg.2018.00487. 10.3389/fpsyg.2018.00487 PMC5902672 29692750

[7] Bürkner, P. C. (2017). Brms: an R package for Bayesian multilevel models using Stan. J Stat Softw, 80(1). https://doi.org/10.18637/jss.v080.i01

[8] Carpenter B, Gelman A, Hoffman MD, Lee D, Goodrich B, Betancourt M, et al. Stan: a probabilistic programming language. J Stat Softw. 2017;76(1). 10.18637/jss.v076.i01. 10.18637/jss.v076.i01 PMC9788645 36568334

[9] Conner, T. S., Brookie, K. L., Carr, A. C., Mainvil, L. A., & Vissers, M. C. M. (2017). Let them eat fruit! The effect of fruit and vegetable consumption on psychological well-being in young adults: a randomized controlled trial. PLoS One, 12(2), 1–19. https://doi.org/10.1371/journal.pone.0171206. 10.1371/journal.pone.0171206 PMC5291486 28158239

[10] Conner, T. S., Brookie, K. L., Richardson, A. C., & Polak, M. A. (2015). On carrots and curiosity: eating fruit and vegetables is associated with greater flourishing in daily life. Br J Health Psychol, 20(2), 413–427. https://doi.org/10.1111/bjhp.12113. 10.1111/bjhp.12113 25080035

[11] Daróczi, G., & Roman, T. (2018). pander: An R “Pandoc” Writer (0.6.3). https://cran.r-project.org/package=pander

[12] Diener, E., & Chan, M. Y. (2011). Happy people live longer: subjective well-being contributes to health and longevity. Appl Psychol, 3(1), 1–43. https://doi.org/10.1111/j.1758-0854.2010.01045.x.

[13] Diener, E., Seligman, M. E. P., Choi, H., & Oishi, S. (2018). Happiest people revisited. Perspect Psychol Sci, 13(2), 176–184. https://doi.org/10.1177/1745691617697077. 10.1177/1745691617697077 29592642

[14] Diener E, Wirtz D, Tov W, Kim-Prieto C, Choi D, Oishi S, et al. New well-being measures: short scales to assess flourishing and positive and negative feelings. Soc Indic Res. 2010;97(2):143–56. 10.1007/s11205-009-9493-y.

[15] Efron B, Hastie T, Johstone I, Tibshirani R (2004). Least angle regression. The Annal.

[16] Efroymson MA. Multiple regression analysis. Math Methods Digital Comput. 1960:191–203 https://ci.nii.ac.jp/naid/10007639144.

[17] Flom, P. L., & Cassell, D. L. (2007). Stopping stepwise: why stepwise and similar selection methods are bad, and what you should use. Northeast SAS User Group (NESUG ) Inc 20th Annual Conference: 11-14th November 2007; Baltimore, Maryland, 1–7.

[18] Gabry, J., Simpson, D., Vehtari, A., Betancourt, M., & Gelman, A. (2019). Visualization in Bayesian workflow. J R Stat Soc Series A, 182(2), 389–402. https://doi.org/10.1111/rssa.12378.

[19] Gelman A, Goodrich B, Gabry J, Vehtari A (2019). R-squared for Bayesian regression models. Am Stat.

[20] Gelman, Andrew, & Rubin, D. B. (1992). Inference from iterative simulation using multiple sequences. Stat Sci, 7(4), 457–511. https://doi.org/10.1214/ss/1177013437.

[21] Goodwin, R. D. (2003). Association between physical activity and mental disorders among adults in the United States. Prev Med, 36(6), 698–703. https://doi.org/10.1016/S0091-7435(03)00042-2. 10.1016/s0091-7435(03)00042-2 12744913

[22] Griskevicius, V., Delton, A. W., Robertson, T. E., & Tybur, J. M. (2011). Environmental contingency in life history strategies: the influence of mortality and socioeconomic status on reproductive timing. J Pers Soc Psychol, 100(2), 241–254. https://doi.org/10.1037/a0021082.Environmental. 10.1037/a0021082 PMC3556268 20873933

[23] Hassmén, P., Koivula, N., & Uutela, A. (2000). Physical exercise and psychological well-being: a population study in Finland. Prev Med, 30(1), 17–25. https://doi.org/10.1006/pmed.1999.0597. 10.1006/pmed.1999.0597 10642456

[24] Hawkins, D. M. (2004). The problem of Overfitting. J Chem Inf Comput Sci, 44(1), 1–12. https://doi.org/10.1021/ci0342472. 10.1021/ci0342472 14741005

[25] Hawkins, D. M., Basak, S. C., & Mills, D. (2003). Assessing model fit by cross-validation. J Chem Inf Comput Sci, 43(2), 579–586. https://doi.org/10.1021/ci025626i. 10.1021/ci025626i 12653524

[26] Heinze, G., Wallisch, C., & Dunkler, D. (2018). Variable selection – a review and recommendations for the practicing statistician. Biom J, 60(3), 431–449. https://doi.org/10.1002/bimj.201700067. 10.1002/bimj.201700067 PMC5969114 29292533

[27] Helwig, N. E. (2017). Adding bias to reduce variance in psychological results: a tutorial on penalized regression. Quant Methods Psychol, 13(1), 1–19. https://doi.org/10.20982/tqmp.13.1.p001

[28] Höge, M., Wöhling, T., & Nowak, W. (2018). A primer for model selection: the decisive role of model complexity. Water Resour Res, 54(3), 1688–1715. https://doi.org/10.1002/2017WR021902.

[29] Hong, S. A., & Peltzer, K. (2017). Dietary behaviour, psychological well-being and mental distress among adolescents in Korea. Child Adolesc Psychiatry Ment Health, 11(1), 1–12. https://doi.org/10.1186/s13034-017-0194-z. 10.1186/s13034-017-0194-z PMC5706161 29209411

[30] Hung, C. I., Weng, L. J., Su, Y. J., & Liu, C. Y. (2006). Depression and somatic symptoms scale: a new scale with both depression and somatic symptoms emphasized. Psychiatry Clin Neurosci, 60(6), 700–708. https://doi.org/10.1111/j.1440-1819.2006.01585.x. 10.1111/j.1440-1819.2006.01585.x 17109704

[31] Huppert, F. A., & So, T. T. C. (2013). Flourishing across Europe: application of a new conceptual framework for defining well-being. Soc Indic Res, 110(3), 837–861. https://doi.org/10.1007/s11205-011-9966-7. 10.1007/s11205-011-9966-7 PMC3545194 23329863

[32] Jacka, F. N., Kremer, P. J., Berk, M., de Silva-Sanigorski, A. M., Moodie, M., Leslie, E. R., Pasco, J. A., & Swinburn, B. A. (2011). A prospective study of diet quality and mental health in adolescents. PLoS One, 6(9), 1–7. https://doi.org/10.1371/journal.pone.0024805. 10.1371/journal.pone.0024805 PMC3177848 21957462

[33] Jacka, F. N., Kremer, P. J., Leslie, E. R., Berk, M., Patton, G. C., Toumbourou, J. W., & Williams, J. W. (2010). Associations between diet quality and depressed mood in adolescents: results from the Australian healthy Neighbourhoods study. Aust N Z J Psychiatry, 44(5), 435–442. https://doi.org/10.3109/00048670903571598. 10.3109/00048670903571598 20397785

[34] Jacka, F. N., O’Neil, A., Opie, R., Itsiopoulos, C., Cotton, S., Mohebbi, M., Castle, D., Dash, S., Mihalopoulos, C., Chatterton, M. Lou, Brazionis, L., Dean, O. M., Hodge, A. M., & Berk, M. (2017). A randomised controlled trial of dietary improvement for adults with major depression (the “SMILES” trial). BMC Med, 15(1), 1–13. https://doi.org/10.1186/s12916-017-0791-y. 10.1186/s12916-017-0791-y PMC5282719 28137247

[35] James, G., Witten, D., Hastie, T., & Tibshirani, R. (2013). An introduction to statistical learning. In Springer Texts in Statistics https://doi.org/10.1016/j.peva.2007.06.006, 64, 9-12, 856, 875.

[36] Kadane, J. B., & Lazar, N. A. (2004). Methods and criteria for model selection. J Am Stat Assoc, 99(465), 279–290. https://doi.org/10.1198/016214504000000269.

[37] Kassambara, A. (2019). ggcorrplot: Visualization of a Correlation Matrix using ‘ggplot2 (0.1.3). https://cran.r-project.org/package=ggcorrplot

[38] Kawada T, Kuratomi Y, Kanai T (2009). Lifestyle determinants of depressive feeling and a feeling of unhappiness among workers: a study in Japan. Work.

[39] Kern, M. L., Della Porta, S. S., & Friedman, H. S. (2014). Lifelong pathways to longevity: personality, relationships, flourishing, and health. J Pers, 82(6), 472–484. https://doi.org/10.1111/jopy.12062. 10.1111/jopy.12062 23927423

[40] Keyes, C. L. M. (2007). Promoting and protecting mental health as flourishing: a complementary strategy for improving National Mental Health. Am Psychol, 62(2), 95–108. https://doi.org/10.1037/0003-066X.62.2.95. 10.1037/0003-066X.62.2.95 17324035

[41] Keyes, C. L. M., Dhingra, S. S., & Simoes, E. J. (2010). Change in level of positive mental health as a predictor of future risk of mental illness. Am J Public Health, 100(12), 2366–2371. https://doi.org/10.2105/AJPH.2010.192245. 10.2105/AJPH.2010.192245 PMC2978199 20966364

[42] Keyes, C. L. M., & Simoes, E. J. (2012). To flourish or not: positive mental health and all-cause mortality. Am J Public Health, 102(11), 2164–2172. https://doi.org/10.2105/AJPH.2012.300918. 10.2105/AJPH.2012.300918 PMC3477942 22994191

[43] Kohout FJ, Berkman LF, Evans DA, Cornoni-Huntley J (1993). Two shorther forms of the CES-D depression symptoms index. J Aging Health.

[44] Koul, A., Becchio, C., & Cavallo, A. (2018). Cross-validation approaches for replicability in psychology*.* Front Psychol, *9*(JUL), 1–4. https://doi.org/10.3389/fpsyg.2018.01117. 10.3389/fpsyg.2018.01117 PMC6043802 30034353

[45] Kuhn, M., & Johnson, K. (2013). Applied predictive modeling (Vol. 26). Springer. https://doi.org/10.1007/978-1-4614-6849-3.

[46] Larmarange, J. (2020). labelled: Manipulating labelled data (2.2.2). https://cran.r-project.org/package=labelled

[47] Li NP, Kanazawa S (2016). Country roads, take me home … to my friends: how intelligence, population density, and friendship affect modern happiness. Br J Psychol.

[48] Liao, Y., Schembre, S. M., O’Connor, S. G. O., Belcher, B. R., Maher, J. P., Dzubur, E., & Dunton, G. F. (2018). An electronic ecological momentary assessment study to examine the consumption of high-fat/high-sugar foods, fruits/ vegetables and affective states among women. J Nutr Educ Behav, 50(6), 626–631. 10.1016/j.jneb.2018.02.003. An. 10.1016/j.jneb.2018.02.003 PMC5995648 29573964

[49] Lund, H. G., Reider, B. D., Whiting, A. B., & Prichard, J. R. (2010). Sleep patterns and predictors of disturbed sleep in a large population of college students. J Adolesc Health, 46(2), 124–132. https://doi.org/10.1016/j.jadohealth.2009.06.016. 10.1016/j.jadohealth.2009.06.016 20113918

[50] McNeish, D. M. (2015). Using Lasso for predictor selection and to assuage Overfitting: a method long overlooked in behavioral sciences. Multivar Behav Res, 50(5), 471–484. https://doi.org/10.1080/00273171.2015.1036965. 10.1080/00273171.2015.1036965 26610247

[51] Meys, J. (2009). *Automated model selection*. https://stackoverflow.com/questions/3701170/stepwise-regression-using-p-values-to-drop-variables-with-nonsignificant-p-value

[52] Moreno-Agostino, D., Caballero, F. F., Martín-María, N., Tyrovolas, S., López-García, P., Rodríguez-Artalejo, F., Haro, J. M., Ayuso-Mateos, J. L., & Miret, M. (2019). Mediterranean diet and wellbeing: evidence from a nationwide survey. Psychol Health, 34(3), 321–335. https://doi.org/10.1080/08870446.2018.1525492. 10.1080/08870446.2018.1525492 30320519

[53] Mujcic, R., & Oswald, A. J. (2016). Evolution of well-being and happiness after increases in consumption of fruit and vegetables. Am J Public Health, 106(8), 1504–1510. https://doi.org/10.2105/AJPH.2016.303260. 10.2105/AJPH.2016.303260 PMC4940663 27400354

[54] Müller, S., Scealy, J. L., & Welsh, A. H. (2013). Model selection in linear mixed models. Stat Sci, 28(2), 135–167. https://doi.org/10.1214/12-STS410.

[55] Oishi, S., Kesebir, S., & Diener, E. (2011). Income inequality and happiness. Psychol Sci, 22(9), 1095–1100. https://doi.org/10.1177/0956797611417262. 10.1177/0956797611417262 21841151

[56] Piironen, J., Paasiniemi, M., & Vehtari, A. (2018). Projective inference in high-dimensional problems: prediction and feature selection. 2015, 1–42. http://arxiv.org/abs/1810.02406

[57] Pilcher, J. J., Ginter, D. R., & Sadowsky, B. (1997). Sleep quality versus sleep quantity: relationships between sleep and measures of health, well-being and sleepiness in college students. J Psychosom Res, 42(6), 583–596. https://doi.org/10.1016/S0022-3999(97)00004-4. 10.1016/s0022-3999(97)00004-4 9226606

[58] Pinquart, M., & Sörensen, S. (2000). Influences of socioeconomic status, social network, and competence on subjective well-being in later life: a meta-analysis. Psychol Aging, 15(2), 187–224. https://doi.org/10.1037/0882-7974.15.2.187. 10.1037//0882-7974.15.2.187 10879576

[59] Piqueras JA, Kuhne W, Vera-Villarroel P, Van Straten A, Cuijpers P. Happiness and health behaviours in Chilean college students: a cross-sectional survey. BMC Public Health. 2011;11(1). 10.1186/1471-2458-11-443. 10.1186/1471-2458-11-443 PMC3125376 21649907

[60] Prendergast, K. B., Mackay, L. M., & Schofield, G. M. (2016a). The clustering of lifestyle Behaviours in New Zealand and their relationship with optimal wellbeing. Int J Behav Med, 23(5), 571–579. https://doi.org/10.1007/s12529-016-9552-0. 10.1007/s12529-016-9552-0 26944753

[61] Prendergast, K. B., Schofield, G. M., & Mackay, L. M. (2016b). Associations between lifestyle behaviours and optimal wellbeing in a diverse sample of New Zealand adults. BMC Public Health, 16(1), 1–11. https://doi.org/10.1186/s12889-016-2755-0. 10.1186/s12889-016-2755-0 PMC4722793 26801097

[62] Pressman, S. D., & Cohen, S. (2005). Does positive affect influence health? Psychol Bull, 131(6), 925–971. https://doi.org/10.1037/0033-2909.131.6.925. 10.1037/0033-2909.131.6.925 16351329

[63] R Core Team. (2019). R: A language and environment for statistical computing. In R Foundation for Statistical Computing, Vienna, Austria (3.6.0). https://www.r-project.org/

[64] Ridner, S. L., Newton, K. S., Staten, R. R., Crawford, T. N., & Hall, L. A. (2016). Predictors of well-being among college students. J Am Coll Heal, 64(2), 116–124. https://doi.org/10.1080/07448481.2015.1085057. 10.1080/07448481.2015.1085057 26630580

[65] Shrout, P. E., Stadler, G., Lane, S. P., Joy McClure, M., Jackson, G. L., Clavél, F. D., Iida, M., Gleason, M. E. J., Xu, J. H., & Bolger, N. (2018). Initial elevation bias in subjective reports. Proc Natl Acad Sci U S A, 115(1), E15–E23. https://doi.org/10.1073/pnas.1712277115. 10.1073/pnas.1712277115 PMC5776801 29255039

[66] Smith, G. (2018). Step away from stepwise. J Big Data, 5(1). https://doi.org/10.1186/s40537-018-0143-6.

[67] Steyerberg, E. W., Eijkemans, M. J. C., Harrell, F. E., & Habbema, J. D. F. (2001). Prognostic modeling with logistic regression analysis: in search of a sensible strategy in small data sets. Med Decis Mak, 21(1), 45–56. https://doi.org/10.1177/0272989X0102100106. 10.1177/0272989X0102100106 11206946

[68] Tay, L., & Kuykendall, L. (2013). Promoting happiness: the malleability of individual and societal subjective wellbeing. Int J Psychol, 48(3), 159–176. https://doi.org/10.1080/00207594.2013.779379. 10.1080/00207594.2013.779379 23551025

[69] Taylor, J., & Tibshirani, R. J. (2015). Statistical learning and selective inference. Proc Natl Acad Sci U S A, 112(25), 7629–7634. https://doi.org/10.1073/pnas.1507583112. 10.1073/pnas.1507583112 PMC4485109 26100887

[70] Thompson, B. (1995). Stepwise regression and stepwise discriminant analysis need not apply here: a guidelines editorial. In Educational and Psychological Measurement (Vol. 55, issue 4, pp. 525–534). https://doi.org/10.1177/0013164495055004001.

[71] Tibshirani, R. (1996). Regression shrinkage and selection via the Lasso. J R Stat Soc Ser B Methodol, 58(1), 267–288. https://doi.org/10.1111/j.2517-6161.1996.tb02080.x.

[72] Tibshirani, R. (2019). *Statistical learning and sparsity*. https://www.stat.auckland.ac.nz/en/about/news-and-events-5/events/events-2019/04/ihaka-lecture-series-2019-statistical-learning-and-sparsity.html

[73] Tierney, N., Cook, D., McBain, M., & Fay, C. (2020). naniar: Data Structures, Summaries, and Visualisations for Missing Data (0.5.0). https://cran.r-project.org/package=naniar

[74] van Erp, S., Oberski, D. L., & Mulder, J. (2019). Shrinkage priors for Bayesian penalized regression. J Math Psychol, 89, 31–50. https://doi.org/10.1016/j.jmp.2018.12.004.

[75] Vehtari, A., Gelman, A., & Gabry, J. (2017). Practical Bayesian model evaluation using leave-one-out cross-validation and WAIC. Stat Comput, 27(5), 1413–1432. 10.1007/s11222-016-9696-4.

[76] Wagner CH (1982). Simpson ’ s paradox in real life author. The American Statistician 1.

[77] Wahl, D. R., Villinger, K., König, L. M., Ziesemer, K., Schupp, H. T., & Renner, B. (2017). Healthy food choices are happy food choices: evidence from a real life sample using smartphone based assessments. Sci Rep, 7(1), 1–8. https://doi.org/10.1038/s41598-017-17262-9. 10.1038/s41598-017-17262-9 PMC5719018 29213109

[78] Wallace, D. D., Boynton, M. H., & Lytle, L. A. (2017). Multilevel analysis exploring the links between stress, depression, and sleep problems among two-year college students. J Am Coll Heal, 65(3), 187–196. https://doi.org/10.1080/07448481.2016.1269111. 10.1080/07448481.2016.1269111 PMC5373919 27937737

[79] White, B. A., Horwath, C. C., & Conner, T. S. (2013). Many apples a day keep the blues away - daily experiences of negative and positive affect and food consumption in young adults. Br J Health Psychol, 18(4), 782–798. https://doi.org/10.1111/bjhp.12021. 10.1111/bjhp.12021 23347122

[80] Whittingham, M. J., Stephens, P. A., Bradbury, R. B., & Freckleton, R. P. (2006). Why do we still use stepwise modelling in ecology and behaviour? J Anim Ecol, 75(5), 1182–1189. https://doi.org/10.1111/j.1365-2656.2006.01141.x. 10.1111/j.1365-2656.2006.01141.x 16922854

[81] Wickham, H. (2017). tidyverse: Easily Install and Load the “Tidyverse” (R package version 1.2.1). https://cran.r-project.org/web/packages/tidyverse/index.html

[82] Wickham, S.-R., Amarasekara, N. A., Bartonicek, A., & Conner, T. S. (2020). The big three health behaviors and mental health and well-being among young adults: a cross-sectional investigation of sleep, exercise, and diet. Front Psychol, 11, 3339. https://doi.org/10.3389/fpsyg.2020.579205. 10.3389/fpsyg.2020.579205 PMC7758199 33362643

[83] Wilke, C. O. (2019). cowplot: Streamlined Plot Theme and Plot Annotations for “ggplot2”. (R package version 0.9.4). https://cran.r-project.org/package=cowplot%0A

[84] Wilson, K. T., Bohnert, A. E., Ambrose, A., Davis, D. Y., Jones, D. M., & Magee, M. J. (2014). Social, behavioral, and sleep characteristics associated with depression symptoms among undergraduate students at a women’s college: a cross-sectional depression survey, 2012. BMC Womens Health, 14(1). https://doi.org/10.1186/1472-6874-14-8. 10.1186/1472-6874-14-8 PMC3893578 24410897

[85] Yarkoni, T. (2020). The generalizability crisis. Behav Brain Sci https://doi.org/10.1017/S0140525X20001685, 1, 37. 10.1017/S0140525X20001685 PMC10681374 33342451

[86] Yarkoni, T., & Westfall, J. (2017). Choosing prediction over explanation in psychology: lessons from machine learning. Perspect Psychol Sci, 12(6), 1100–1122. https://doi.org/10.1177/1745691617693393. 10.1177/1745691617693393 PMC6603289 28841086

[87] Zou, H., & Hastie, T. (2005). Regularization and variable selection via the elastic net. J R Stat Soc. Series B: Statistical Methodology, 67(2), 301–320. https://doi.org/10.1111/j.1467-9868.2005.00503.x.

