# Supplementary material for: The value of Bayesian predictive projection for variable selection: an example of selecting lifestyle predictors of young adult well-being
Source: BMC Public Health. 2021 Apr 9;21:695. doi: 10.1186/s12889-021-10690-3 (PMC8033696; doi:10.1186/s12889-021-10690-3)
Supplement: Supplementary file 1 — Additional file 1. Supplementary materials. [file 12889_2021_10690_MOESM1_ESM.pdf]

Supplementary materials

The value of Bayesian predictive projection for variable selection: An example selecting lifestyle predictors of young adult well-being

Adam Bartonicek

Data pre-processing

Packages

I used the following packages for pre-processing:

```
library(tidyverse)
library(haven)
library(naniar)
library(labelled)
library(pander)

theme_set(theme_classic())
```

Data

I first loaded up the following files with data from the Dunedin Daily Life survey 2011-2014:

```
data_subjectlist_raw <- read_sav('data/raw/subject_list.sav') # Master subject list
data_daily_raw <- read_sav('data/raw/DailySurvey_2011-2014.sav') # Daily survey
data_initial_raw <- read_sav('data/raw/InitialSurvey_2011-2014.sav') # Initial survey
data_followup_raw <- read_sav('data/raw/FollowupSurvey_2011-2014.sav') # Follow up survey
data_clinic_raw <- read_sav('data/raw/Clinic_BMI_CRP_2011-2014-corrected.sav') # Clinic data
```

Daily life survey

Below is a list of all variables from the daily & the visualized missingness across participants in 2013-2014. Not all variables were collected across the entirety of the study (see Figure 1 below).

```
data_daily_raw %>%
  map(attributes) %>%
  map('label') %>%
  enframe() %>%
  unnest(value) %>%
  rename(`Variable label` = name, Description = value) %>%
  mutate(Description = ifelse(str_detect(`Variable label`, 'daff\\d+'),
                       str_replace(Description, 'Mood', 'Affect'),
                       Description)) %>%
```

```
mutate(Description = str_replace_all(Description, "’", "'")) %>%
mutate(Description = str_replace_all(Description, """, "'")) %>%
mutate(Description = str_replace_all(Description, """, "'")) %>%
pander(split.cell = 80, split.table = Inf, justify = 'left',
       caption = 'All daily diary variables recorded across
       the Daily Life Study (2011-2014)')
```

Table 1: All daily diary variables recorded across the Daily Life Study (2011-2014)

Variable label	Description
ID	Participant ID
year	Year of Data Collection
wave	Wave of participation
wave_day	Wave and Day of Initial Session
ndiary	Number of diaries completed
day	Day of diary study (1 - 13)
DOW	Day of Week
Month	Month of Year
date	Date of survey entry
time	Time of survey entry
datetimestamp	Original Time/Date stamp from survey system
diary	Whether a diary survey was done that day
paper	Paper copy used
nfun	Previous nights FUN rating - How much fun did you have last night?
nenjoy	Previous nights ENJOYMENT rating - How much did you enjoy what you did last night?
npleasure	Previous nights PLEASURE rating - How much pleasure did you experience last night?
nmeaning	Previous nights MEANINGFUL rating- How meaningful were your experiences last night?
ncreate	Previous nights CREATIVITY rating - How creative were you last night?
npac	Last night’s physical activity (number of minutes)
ndrink	Number of Standard Drinks from previous night
ndrinkst	Time Started Drinking
ndrinkend	Time Finished Drinking
nfruit	LAST NIGHT - servings of fruit
nchips	LAST NIGHT - servings of hot chips, French fries, wedges or kumara chips
nveg	LAST NIGHT - servings of vegetables
nsweets	LAST NIGHT - servings of lollies, sweets, chocolate, or other confectionary items
nsoftdrk	LAST NIGHT - servings of regular soft drinks or energy drinks (not diet)
dmood1	Daily Mood - How HAPPY participants felt today
dmood2	Daily Mood - How EXCITED participants felt today
dmood3	Daily Mood - How NERVOUS participants felt today
dmood4	Daily Mood - How DEJECTED participants felt today
dmood5	Daily Mood - How IRRITABLE participants felt today
dmood6	Daily Mood - How CHEERFUL participants felt today
dmood7	Daily Mood - How PLEASED participants felt today
dmood8	Daily Mood - How CALM participants felt today
dmood9	Daily Mood - How INSPIRED participants felt today
dmood10	Daily Mood - How INACTIVE participants felt today
dmood11	Daily Mood - How HOSTILE participants felt today
dmood12	Daily Mood - How SAD participants felt today

Variable label	Description
dmood13	Daily Mood - How ANGRY participants felt today
dmood14	Daily Mood - How ENERGETIC participants felt today
dmood15	Daily Mood - How ACTIVE participants felt today
dmood16	Daily Mood - How ENTHUSIASTIC participants felt today
dmood17	Daily Mood - How UNHAPPY participants felt today
dmood18	Daily Mood - How ANXIOUS participants felt today
dmood19	Daily Mood - How TENSE participants felt today
dmood20	Daily Mood - How MOTIVATED participants felt today
dmood21	Daily Mood - How ENGAGED participants felt today
dmood22	Daily Mood - How AWAKE participants felt today
dmood23	Daily Mood - How CONTENT participants felt today
dmood24	Daily Mood - How GOOD participants felt today
dmood25	Daily Mood - How TIRED participants felt today
dmood26	Daily Mood - How RELAXED participants felt today
dmood27	Daily Mood - How UNENGAGED participants felt today
dmood28	Daily Mood - How ALERT participants felt today
dmood29	Daily Mood - How BAD participants felt today
rundown	How physically RUNDOWN participants felt today
cold	The extent to which participants felt they had a COLD or FLU on today
hangover	How much of a HANGOVER participants felt today
tired	How TIRED participants felt today
concent	How much trouble participants had CONCENTRATING today
refresh	How REFRESHED participants felt when they woke up today
sleep	How many hours SLEEP participants had the night before (rounded to nearest hour)
dstress	How much STRESS participants had been under today
devstres	How STRESSFUL was most stressful EVENT of the day?
dwrkld	WORKLOAD - The extent to which participants felt they were on top of their responsibilities and commitments today
daff1	Daily Affect - How MOTIVATED participants were today
daff2	Daily Affect - How POSITIVE participants were today
daff3	Daily Affect - How ENERGETIC participants were today
daff4	Daily Affect - How NEGATIVE participants were today
daff5	Daily Affect - How TENSE participants were today
dfs1	Daily Flourishing Scale - led a purposeful and meaningful life today
dfs2	Daily Flourishing Scale - social relationships supportive and rewarding
dfs3	Daily Flourishing Scale - engaged and interested in daily activities
dfs4	Daily Flourishing Scale - contributed to the happiness of others
dfs5	Daily Flourishing Scale - felt competent and capable in important activities
dfs6	Daily Flourishing Scale - was a good person and lived a good life today
dfs7	Daily Flourishing Scale - felt optimistic about my future
dfs8	Daily Flourishing Scale - people respected me
dewb1	Daily Eudaimonic Well-being Scale - Today, I tended to socialize with other people
dewb2	Daily Eudaimonic Well-being Scale - Today, I found myself looking for new opportunities to grow as a person (e.g., new knowledge, people, resources)
dewb3	Daily Eudaimonic Well-being Scale - Today, I felt like a failure
dewb4	Daily Eudaimonic Well-being Scale - Today, I found myself doing things purely for the interest and enjoyment of doing them
dewb5	Daily Eudaimonic Well-being Scale - Today, I felt satisfied with myself.
dewb6	Daily Eudaimonic Well-being Scale - Today, I took a positive attitude toward myself.
dewb7	Daily Eudaimonic Well-being Scale - Today, I thought I am no good at all

Variable label	Description
dewb8	Daily Eudaimonic Well-being Scale - Today, I felt that people in my life cared about me
dlet1_7scale	Daily Life Engagement Test - Today, there was enough purpose in my life.
dlet2_7scale	Daily Life Engagement Test - Today, the things I did were all worthwhile
dlet3_7scale	Daily Life Engagement Test - Today, most of what I did seemed trivial and unimportant to me.
dlet4_7scale	Daily Life Engagement Test - Today, I valued my activities a lot.
dlet5_7scale	Daily Life Engagement Test - Today, I didn't care very much about the things I did.
dlet1_5scale	Daily Life Engagement Test - Today, there was enough purpose in my life.
dlet2_5scale	Daily Life Engagement Test - Today, the things I did were all worthwhile
dlet3_5scale	Daily Life Engagement Test - Today, most of what I did seemed trivial and unimportant to me.
dlet4_5scale	Daily Life Engagement Test - Today, I valued my activities a lot.
dlet5_5scale	Daily Life Engagement Test - Today, I didn't care very much about the things I did.
dcei1	Daily Curiosity and Exploration Inventory - Today, I actively sought out as much information as I could in a new situation.
dcei2	Daily Curiosity and Exploration Inventory - Today, when I participated in an activity, I got so involved that I lost track of time.
dcei3	Daily Curiosity and Exploration Inventory - Today, I found myself looking for new opportunities to grow as a person.
dcei4	Daily Curiosity and Exploration Inventory - Today, I did not probe deeply into new situations or things.
dcei5	Daily Curiosity and Exploration Inventory - Today, when I was actively interested in something, it took a great deal to interrupt me.
dcei6	Daily Curiosity and Exploration Inventory - Today, I would have been described as 'extremely intense' when in the middle of doing something.
dcei7	Daily Curiosity and Exploration Inventory - Today, I looked for new things or experiences.
dfun	How much FUN participants had today
denjoy	How much ENJOYMENT participants had today
dpleasure	How much PLEASURE participants had today
dmeaning	How MEANINGFUL were experiences today
dcreate	Today's CREATIVITY rating - How creative were you today?
dpac	Physical Activity Questions - How much time particpant spent doing PHYSICAL ACTIVITIES
dse	Daily Sun Exposure Questionnaire - How many hours spent outside during DAYLIGHT hours
dfruit	TODAY - servings of fruit
dchips	TODAY - servings of hot chips, French fries, wedges or kumara chips
dveg	TODAY - servings of vegetables
dsweets	TODAY - servings of lollies, sweets, chocolate, or other confectionary items
dsoftdrk	TODAY - servings of regular soft drinks or energy drinks (not diet)
dunwb1	Behaviour Change Questionnaire - The strongest UNWANTED BEHAVIOUR participants choose to inhibit or change today
dunwb3ot	Behaviour Change Questionnaire - OTHER UNWANTED BEHAVIOUR participants choose to inhibit or change today
dunwb2	Behaviour Change Questionnaire - WHEN did this situation happen
dunwb3_1	Behaviour Change Questionnaire - Before the situation, the extent to which participants felt GOOD
dunwb3_2	Behaviour Change Questionnaire - Before the situation, the extent to which participants felt TIRED

Variable label	Description
dunwb3_3	Behaviour Change Questionnaire - Before the situation, the extent to which participants felt CALM
dunwb3_4	Behaviour Change Questionnaire - Before the situation, the extent to which participants felt AWAKE
dunwb3_5	Behaviour Change Questionnaire - Before the situation, the extent to which participants felt BAD
dunwb3_6	Behaviour Change Questionnaire - Before the situation, the extent to which participants felt NERVOUS
dunwb4	Behaviour Change Questionnaire - HOW OFTEN participants performed the unwanted behaviour in the PAST
dunwb5	Behaviour Change Questionnaire - How much would PERFORMING the unwanted behaviour would make them feel GOOD
dunwb6	Behaviour Change Questionnaire - How much would PERFORMING the unwanted behaviour would make them feel BAD
dunwb7	Behaviour Change Questionnaire - The STRATEGY participants used to stop them from performing the unwanted behaviour
dunwb7ot	Behaviour Change Questionnaire - OTHER STRATEGIES participants used to stop them from performing the unwanted behaviour
dunwb8	Behaviour Change Questionnaire - How SUCCESSFUL did the participant believe they were in changing their unwanted behaviour
dunwb9_1	Behaviour Change Questionnaire - How participants felt after their situtation, how GOOD
dunwb9_2	Behaviour Change Questionnaire - How participants felt after their situtation, how TIRED
dunwb9_3	Behaviour Change Questionnaire - How participants felt after their situtation, how CALM
dunwb9_4	Behaviour Change Questionnaire - How participants felt after their situtation, how AWAKE
dunwb9_5	Behaviour Change Questionnaire - How participants felt after their situtation, how BAD
dunwb9_6	Behaviour Change Questionnaire - How participants felt after their situtation, how NERVOUS
dunwb	Behaviour Change Questionnaire - Any UNWANTED BEHAVIOUR to inhibit or change today?
dunwb6b	Behaviour Change Questionnaire - How much did you DESIRE to perform the unwanted behaviour
snhour	Social Networking - hours spent today
snmin	Social Networking - minutes spent today
sntime	Social Networking - total time spent (snhour * 60) + (snmin)
sncheck	Social Networking - # times checked today
dnathour	Hours in nature today
dnatmin	Minutes in nature today
dnat	Total time in nature today (dnathour x 60, + dnatmin)
dnat_cat	Categories of time in nature today
dnatint	Whether time in nature was intentional
dnatdes	Type(s) of green space(s) visited today
dnatq1	Quality of experience in nature: Being there was an escape
dnatq2	Quality of experience in nature: Break from routine
dnatq3	Quality of experience in nature: Fascinating qualities
dnatq4	Quality of experience in nature: Attention drawn to interesting things
dnatq5	Quality of experience in nature: Awakened my curiosity
dnatq6	Quality of experience in nature: Much to explore and discover

```r
data_daily_raw %>%
  group_by(ID) %>%
  summarise_all(mean, na.rm = TRUE) %>%
  select(-c(ID:paper),
         -starts_with(c('dmood', 'dlet', 'swl', 'dfs',
                        'daff', 'dewb', 'dcei', 'dunwb'))) %>%
  vis_miss()
```

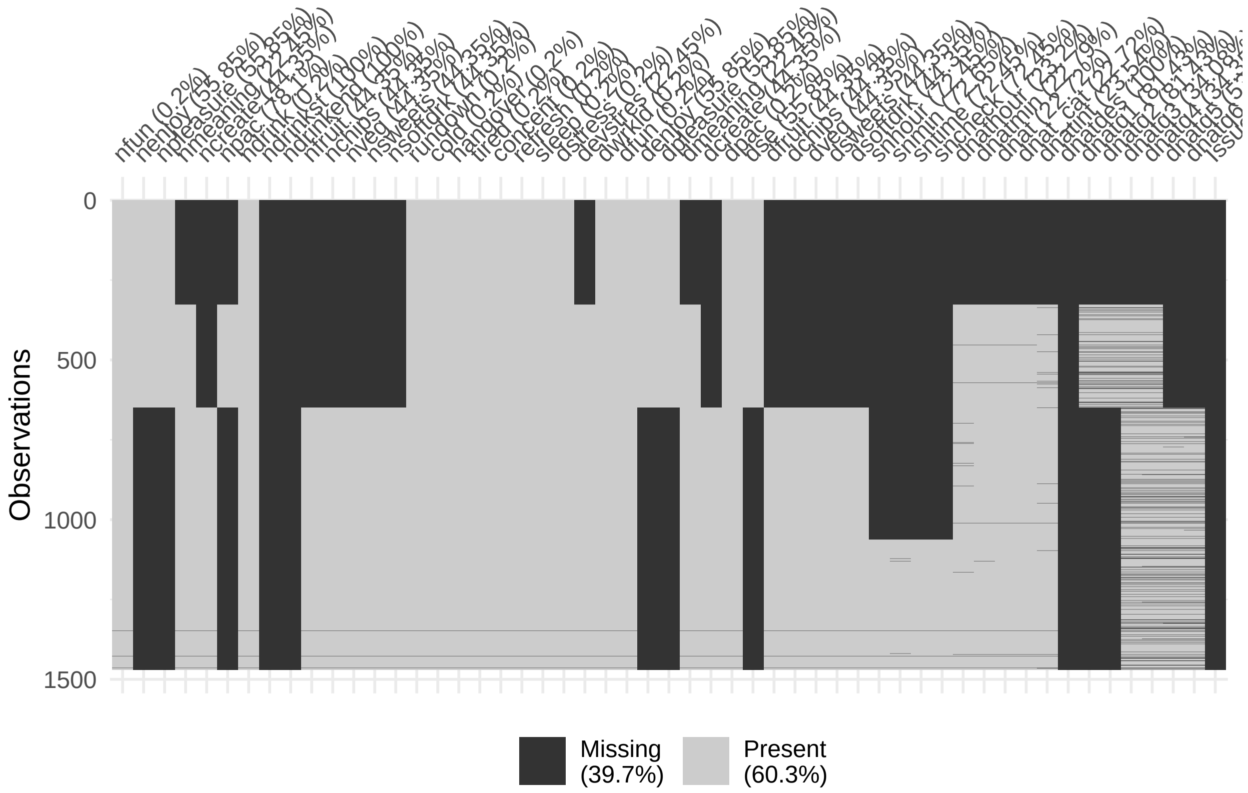

Figure 1: Missingness across all variables in the Daily Life Study (2011-2014), excluding admin variables & mood/behaviour scales

(Some variables were dropped from or added to the survey across the years, and these had records missing from large fractions of the participants. The mood/behaviour variables were excluded from the plot below to avoid overlapping labels on the x-axis)

From the daily survey, I kept only the records of participants who had participated in the survey in 2013-2014 and had submitted 7+ diary records. Additionally, I dropped the first two days of the study, to avoid initial elevation bias:

```r
data_daily_processing1 <- data_daily_raw %>%
  filter(year %in% c(2013, 2014) & diary == 1 & ndiary >= 7 & !(day %in% c(1, 2)))
```

As for variables, I selected all continuous variables that: 1) were collected during the entirety of 2013-2014, 2) were not too closely semantically related to the items from the Flourishing Scale (Diener et al. 2009). For example, I did not include the following items: had fun today, felt on top of workload today, felt creative today, the well-being/curiosity/unwanted behaviour scale items - as these are very similar to some of the

flourishing scale items. Additionally I also selected the flourishing scale items (i.e. the dependet variable), the day variable, and the ID variable. Below is the list of all selected variables:

Table 2: Daily diary variables for analysis from the Daily Life Study (2013-2014)

Variable label	Name	Short name	Description
ID	ID	ID	Participant ID
day	Day	Day	Day of diary study (1 - 13)
ndrink	Standard drinks last night	Drinks	Number of Standard Drinks from previous night
nfruit	Servings of fruit last night	Fruit night	LAST NIGHT - servings of fruit
nchips	Servings of chips last night	Chips night	LAST NIGHT - servings of hot chips, French fries, wedges or kumara chips
nveg	Servings of veges last night	Veges night	LAST NIGHT - servings of vegetables
nsweets	Servings of sweets last night	Sweets night	LAST NIGHT - servings of lollies, sweets, chocolate, or other confectionary items
nsoftdrk	Servings of soft drink last night	Soft drink night	LAST NIGHT - servings of regular soft drinks or energy drinks (not diet)
rundown	Felt rundown today	Rundown	How physically RUNDOWN participants felt today
cold	Felt cold or flu today	Cold/flu	The extent to which participants felt they had a COLD or FLU on today
hangover	Felt hangover today	Hangover	How much of a HANGOVER participants felt today
tired	Felt tired today	Tired	How TIRED participants felt today
concent	Had trouble concentrating today	Distracted	How much trouble participants had CONCENTRATING today
refresh	Felt refreshed after waking up today	Stress	How REFRESHED participants felt when they woke up today
sleep	Hours slept night before	Stressful event	How many hours SLEEP participants had the night before (rounded to nearest hour)
dstress	Felt stressed today	Refreshed	How much STRESS participants had been under today
devstres	Most stressful event today	Sleep quantity	How STRESSFUL was most stressful EVENT of the day?
dpac	Minutes physically active today	Physical act.	Physical Activity Questions - How much time particpant spent doing PHYSICAL ACTIVITIES
dfruit	Servings of fruit today	Fruit today	TODAY - servings of fruit
dchips	Servings of chips today	Chips today	TODAY - servings of hot chips, French fries, wedges or kumara chips
dveg	Servings of veges today	Veges today	TODAY - servings of vegetables
dsweets	Servings of sweets today	Sweets today	TODAY - servings of lollies, sweets, chocolate, or other confectionary items
dsoftdrk	Servings of soft drink today	Soft drink today	TODAY - servings of regular soft drinks or energy drinks (not diet)
dnat	Time spend in nature today	Nature	Total time in nature today (dnathour x 60, + dnatmin)
dfs1	Daily flourishing 1	DFS1	Daily Flourishing Scale - led a purposeful and meaningful life today

Variable label	Name	Short name	Description
dfs2	Daily flourishing 2	DFS2	Daily Flourishing Scale - social relationships supportive and rewarding
dfs3	Daily flourishing 3	DFS3	Daily Flourishing Scale - engaged and interested in daily activities
dfs4	Daily flourishing 4	DFS4	Daily Flourishing Scale - contributed to the happiness of others
dfs5	Daily flourishing 5	DFS5	Daily Flourishing Scale - felt competent and capable in important activities
dfs6	Daily flourishing 6	DFS6	Daily Flourishing Scale - was a good person and lived a good life today
dfs7	Daily flourishing 7	DFS7	Daily Flourishing Scale - felt optimistic about my future
dfs8	Daily flourishing 8	DFS8	Daily Flourishing Scale - people respected me

```r
data_daily_processing2 <- data_daily_processing1 %>%
  dplyr::select(ID, day, ndrink, nfruit:nsoftdrk, rundown:devstres,
         dpac, dfruit:dsoftdrk, dnat, starts_with('dfs')) %>%
  rename_at(vars(- ID), tolower)

glimpse(data_daily_processing2)
```

```
## Rows: 7,754
## Columns: 32
## $ ID       <dbl> 666, 666, 666, 666, 666, 666, 666, 667, 667, 667, 667, 667...
## $ day      <dbl> 3, 4, 6, 7, 8, 9, 10, 3, 4, 5, 6, 7, 8, 9, 10, 11, 12, 13,...
## $ ndrink   <dbl> 0, 0, 0, 0, 0, 0, 0, 0, 0, 0, 0, 0, 0, 0, 0, 0, 0, 0, 0, 0...
## $ nfruit   <dbl+lbl> 1, 0, 3, 0, 0, 0, 0, 2, 2, 2, 1, 0, 0, 0, 1, 0, 1, 1, ...
## $ nchips   <dbl+lbl> 0, 0, 0, 0, 0, 0, 0, 0, 0, 0, 0, 0, 0, 0, 0, 0, 0, 0, ...
## $ nveg     <dbl+lbl> 2.0, 1.0, 2.0, 2.0, 1.0, 2.0, 2.0, 2.0, 3.0, 3.0, 3.0,...
## $ nsweets  <dbl+lbl> 0.0, 1.0, 1.0, 1.0, 1.0, 2.0, 0.0, 4.0, 0.0, 0.0, 0.0,...
## $ nsoftdrk <dbl+lbl> 0, 0, 2, 0, 0, 1, 0, 0, 0, 0, 0, 0, 0, 0, 0, 0, 0, 0, ...
## $ rundown  <dbl+lbl> 1, 0, 1, 0, 1, 1, 1, 2, 2, 2, 1, 2, 1, 1, 1, 1, 1, 0, ...
## $ cold     <dbl+lbl> 0, 0, 0, 0, 0, 0, 0, 3, 3, 2, 1, 2, 1, 1, 1, 1, 1, 0, ...
## $ hangover <dbl+lbl> 0, 0, 0, 0, 0, 0, 0, 0, 0, 0, 0, 0, 0, 0, 0, 0, 0, 0, ...
## $ tired    <dbl+lbl> 4, 3, 4, 2, 3, 4, 4, 1, 1, 1, 1, 1, 1, 1, 1, 1, 2, 1, ...
## $ concent  <dbl+lbl> 4, 3, 4, 2, 4, 4, 4, 2, 2, 1, 1, 1, 1, 2, 1, 2, 3, 3, ...
## $ refresh  <dbl+lbl> 2, 1, 0, 0, 1, 0, 1, 2, 1, 1, 1, 1, 1, 1, 1, 1, 1, 1, ...
## $ sleep    <dbl> 7.0, 6.5, 8.0, 7.0, 8.5, 5.0, 5.0, 8.0, 8.5, 7.5, 7.0, 8.0...
## $ dstress  <dbl+lbl> 1, 1, 2, 2, 2, 3, 4, 1, 1, 1, 1, 1, 1, 1, 2, 1, 2, 1, ...
## $ devstres <dbl+lbl> 2, 0, 2, 1, 1, 2, 0, 1, 1, 1, 1, 1, 1, 1, 2, 1, 2, 1, ...
## $ dpac     <dbl> 30, 30, 0, 0, 0, 0, 0, 30, 0, 60, 90, 30, 90, 20, 90, 0, 0...
## $ dfruit   <dbl> 1, 0, 1, 1, 0, 0, 1, 4, 3, 0, 0, 0, 2, 2, 2, 3, 3, 2, 3, 1...
## $ dchips   <dbl> 1, 0, 0, 0, 0, 0, 0, 0, 0, 0, 0, 0, 0, 0, 0, 0, 0, 0, 0, 0...
## $ dveg     <dbl> 0, 0, 1, 1, 2, 1, 1, 3, 2, 2, 2, 2, 1, 2, 0, 1, 0, 1, 3, 2...
## $ dsweets  <dbl> 0.0, 0.0, 1.0, 3.0, 2.0, 1.0, 2.0, 0.0, 0.0, 0.5, 1.0, 0.0...
## $ dsoftdrk <dbl> 0, 0, 0, 0, 0, 0, 0, 0, 0, 0, 0, 0, 0, 0, 0, 0, 0, 0, 0, 0...
## $ dnat     <dbl> 0, 0, 0, 0, 0, 0, 0, 0, 0, 0, 120, 0, 120, 20, 0, 0, 0, 12...
## $ dfs1     <dbl+lbl> 4, 4, 1, 3, 4, 2, 1, 5, 4, 5, 5, 6, 5, 4, 5, 5, 5, 5, ...
## $ dfs2     <dbl+lbl> 6, 5, 4, 4, 3, 2, 1, 5, 5, 5, 5, 6, 6, 5, 5, 5, 7, 5, ...
## $ dfs3     <dbl+lbl> 2, 4, 1, 2, 2, 1, 1, 3, 3, 4, 5, 4, 3, 3, 5, 3, 3, 4, ...
```

```
## $ dfs4     <dbl+lbl> 4, 4, 5, 4, 2, 4, 1, 4, 5, 5, 4, 6, 5, 3, 5, 5, 6, 4, ...
## $ dfs5     <dbl+lbl> 3, 4, 1, 2, 2, 1, 1, 5, 5, 5, 5, 5, 5, 3, 5, 4, 3, 5, ...
## $ dfs6     <dbl+lbl> 4, 5, 3, 3, 2, 3, 1, 5, 5, 5, 5, 5, 5, 5, 5, 5, 5, 5, ...
## $ dfs7     <dbl+lbl> 4, 4, 2, 3, 2, 2, 1, 5, 5, 4, 4, 5, 5, 5, 4, 5, 5, 4, ...
## $ dfs8     <dbl+lbl> 4, 4, 4, 4, 4, 4, 4, 4, 4, 4, 5, 5, 5, 5, 5, 5, 5, 5, ...
```

Next, I visualized the missingness in the selected variables:

```r
vis_miss(data_daily_processing2)
```

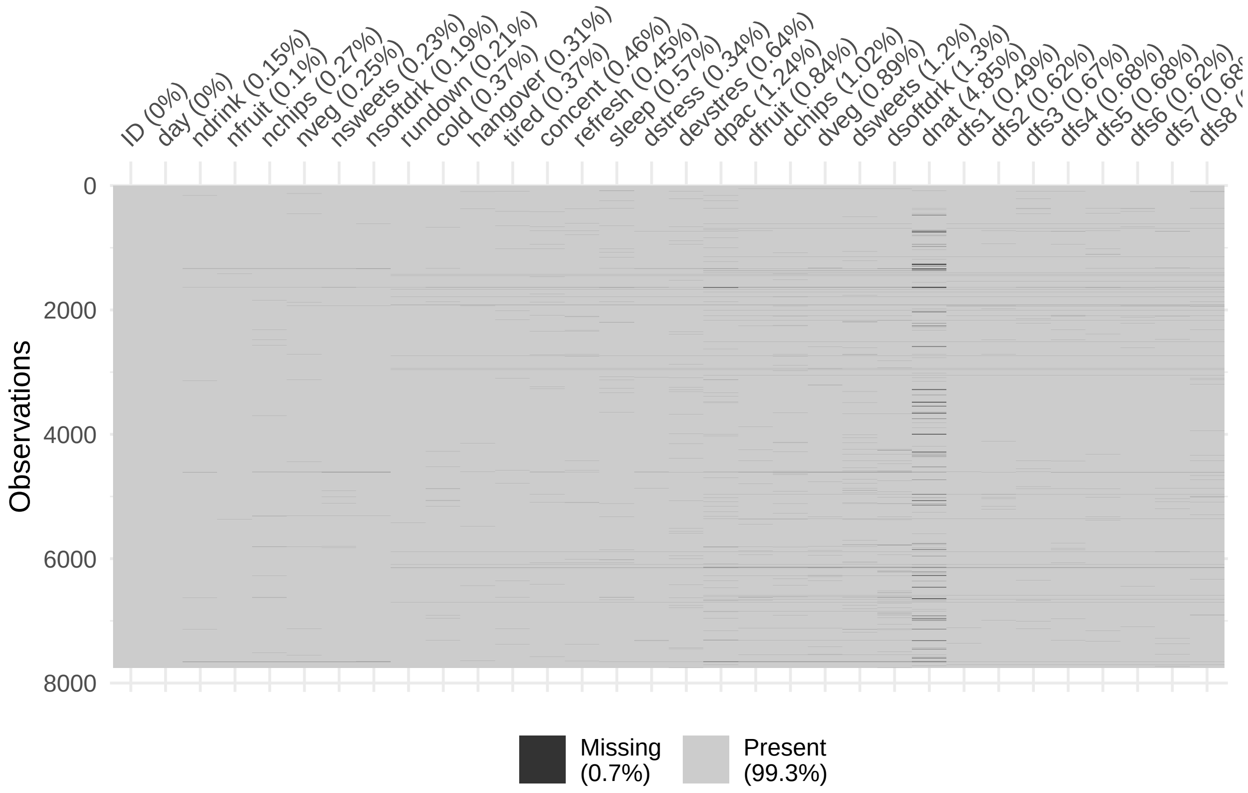

Figure 2: Missingness across selected variables in the Daily Life Study (2013-2014)

There were only very few missing records (0.672%) across all records. Next, I looked at the missingness across variables:

```r
data_daily_processing2 %>%
  dplyr::select(- ID) %>%
  gather('variable', 'value', everything()) %>%
  group_by(variable) %>%
  summarise(prop_missing = mean(is.na(value))) %>%
  arrange(desc(prop_missing)) %>%
  pander(split.cell = 80, split.table = Inf, justify = 'left',
         caption = 'Proportion of missing values across selected variables')
```

```
## Warning: attributes are not identical across measure variables;
## they will be dropped
```

```
## `summarise()` ungrouping output (override with `.groups` argument)
```

Table 3: Proportion of missing values across selected variables

variable	prop_missing
dnat	0.04849
dsoftdrk	0.01303
dpac	0.01238
dsweets	0.01199
dchips	0.01019
dveg	0.008899
dfruit	0.008383
dfs8	0.007867
dfs4	0.006835
dfs5	0.006835
dfs7	0.006835
dfs3	0.006706
devstres	0.006448
dfs2	0.00619
dfs6	0.00619
sleep	0.005674
dfs1	0.004901
concent	0.004643
refresh	0.004514
cold	0.00374
tired	0.00374
dstress	0.003353
hangover	0.003095
nchips	0.002708
nveg	0.00245
nsweets	0.002321
rundown	0.002063
nsoftdrk	0.001934
ndrink	0.001548
nfruit	0.001032
day	0

```r
data_daily_processing2 %>%
  group_by(ID) %>%
  summarise(percent_missing = mean(is.na(dnat)),
            n_missing = sum(is.na(dnat))) %>%
  arrange(desc(percent_missing)) %>%
  rownames_to_column() %>%
  mutate(ID = str_replace(ID, '\\d{2}$', '**')) %>%
  head(n = 35) %>%
  pander(split.cell = 80, split.table = Inf, justify = 'left',
         caption = 'Proportion of missing time spent in nature records across
         participants*')
```

```
## `summarise()` ungrouping output (override with `.groups` argument)
```

Table 4: Proportion of missing time spent in nature records across participants*

rowname	ID	percent_missing	n_missing
1	10**	1	11
2	14**	1	7
3	8**	0.9091	10
4	10**	0.9091	10
5	7**	0.9	9
6	8**	0.9	9
7	11**	0.9	9
8	10**	0.8889	8
9	7**	0.875	7
10	8**	0.8571	6
11	7**	0.8182	9
12	13**	0.8182	9
13	13**	0.8182	9
14	9**	0.7778	7
15	10**	0.7778	7
16	7**	0.7273	8
17	12**	0.7273	8
18	13**	0.7273	8
19	8**	0.6364	7
20	14**	0.6364	7
21	14**	0.6364	7
22	10**	0.6	6
23	11**	0.6	3
24	11**	0.6	6
25	13**	0.5714	4
26	13**	0.5556	5
27	14**	0.5556	5
28	9**	0.5455	6
29	12**	0.5455	6
30	12**	0.5455	6
31	8**	0.5	5
32	14**	0.5	4
33	14**	0.5	5
34	7**	0.4545	5
35	8**	0.4444	4

```r
data_daily_processing2 <- data_daily_processing2 %>%
  dplyr::select(- dnat)
```

(*ID numbers are masked to preserve anonymity)

While most variables contained only few missing values (< 1.3%), the time spent in nature variable (`dnat`) contained 4.85% missing values. Looking at the missing values, there were 30 participants missing most (>50%) of their time spent in nature records. Because of the high number of missing values, and also because it may be not as theoretically meaningful as the other variables, I decided to drop the `dnat` entirely. After excluding `dnat`, there were very few (0.5 %) missing values left. As the values were going to be averaged across days, I did not impute them.

Next, I took the mean of the flourishing scale items to create the daily flourishing variable, for each record.

Following that, I centered all variables and scaled them to 1 unit variance (including the newly created daily flourishing variable).

```
data_daily_processing3 <- data_daily_processing2 %>%
  mutate(dfs_mean = dplyr::select(., starts_with('dfs')) %>%
           rowMeans(na.rm = TRUE)) %>%
  dplyr::select(- matches('dfs\\d'))

data_daily_processing4 <- data_daily_processing3 %>%
  group_by(ID) %>%
  summarize_at(vars(- c(day)), ~ mean(.x, na.rm = TRUE))

glimpse(data_daily_processing4)
```

```
## Rows: 796
## Columns: 23
## $ ID       <dbl> 666, 667, 668, 669, 670, 671, 672, 673, 674, 675, 676, 677...
## $ ndrink   <dbl> 0.0000000, 0.0000000, 4.5000000, 1.0909091, 5.1428571, 1.5...
## $ nfruit   <dbl> 0.57142857, 0.90909091, 1.00000000, 0.54545455, 0.28571429...
## $ nchips   <dbl> 0.00000000, 0.00000000, 0.45000000, 0.31818182, 0.42857143...
## $ nveg     <dbl> 1.7142857, 1.5909091, 1.8000000, 1.1818182, 1.2142857, 0.3...
## $ nsweets  <dbl> 0.8571429, 0.7727273, 0.5000000, 0.6363636, 0.5714286, 0.5...
## $ nsoftdrk <dbl> 0.42857143, 0.00000000, 0.25000000, 0.54545455, 0.42857143...
## $ rundown  <dbl> 0.7142857, 1.2727273, 1.7000000, 0.9090909, 3.1428571, 1.9...
## $ cold     <dbl> 0.00000000, 1.45454545, 1.00000000, 1.45454545, 0.14285714...
## $ hangover <dbl> 0.0000000, 0.0000000, 0.6000000, 0.2727273, 2.1428571, 0.3...
## $ tired    <dbl> 3.4285714, 1.0909091, 2.4000000, 1.5454545, 3.5714286, 2.0...
## $ concent  <dbl> 3.5714286, 1.7272727, 2.6000000, 1.0909091, 3.2857143, 1.5...
## $ refresh  <dbl> 0.7142857, 1.0909091, 1.9000000, 1.3636364, 1.1428571, 1.3...
## $ sleep    <dbl> 6.714286, 7.863636, 8.150000, 7.545455, 8.357143, 8.045455...
## $ dstress  <dbl> 2.1428571, 1.1818182, 1.0000000, 2.4545455, 2.8571429, 1.2...
## $ devstres <dbl> 1.1428571, 1.1818182, 0.8000000, 1.8181818, 2.0000000, 1.0...
## $ dpac     <dbl> 8.5714286, 40.0000000, 53.5000000, 0.9090909, 7.1428571, 0...
## $ dfruit   <dbl> 0.5714286, 1.9090909, 1.6000000, 0.5454545, 0.7142857, 0.2...
## $ dchips   <dbl> 0.1428571, 0.0000000, 0.1000000, 0.0000000, 0.5714286, 0.5...
## $ dveg     <dbl> 0.8571429, 1.4545455, 1.9000000, 0.8636364, 0.7142857, 1.3...
## $ dsweets  <dbl> 1.28571429, 0.77272727, 0.10000000, 0.36363636, 0.57142857...
## $ dsoftdrk <dbl> 0.00000000, 0.00000000, 0.30000000, 0.22727273, 0.28571429...
## $ dfs_mean <dbl> 2.892857, 4.693182, 4.475000, 3.875000, 3.982143, 3.954545...
```

Next, I appended the initial, clinic, and subject list data and selected the following demographic variables as additional predictors: age, gender, ethnicity, ses items 1 through 3, and BMI. Following that, I specified the gender and ethnic variables as factors, took an average of the three SES items, centered and scaled age, BMI and the newly created SES variable, and grouped all but the three largest ethnicity categories together. There were 4 participants missing their BMI information and 1 participant missing two out of the three SES items, so I dropped these participants.

```
data_all_processing1 <- data_daily_processing4 %>%
  left_join(data_initial_raw, by = 'ID') %>%
  left_join(data_clinic_raw, by = 'ID') %>%
  left_join(data_subjectlist_raw, by = 'ID') %>%
  dplyr::select(ID:dfs_mean, age, gender, ethnic, BMI, ses1:ses3)
```

```r
data_all_processing2 <- data_all_processing1 %>%
  mutate_at(vars(gender, ethnic), ~ to_factor(.x)) %>%
  mutate(ID = factor(ID),
         ethnic = fct_lump(ethnic, 3),
         ses = dplyr::select(., starts_with('ses')) %>% rowMeans()) %>%
  mutate_if(is.numeric, ~ (.x - mean(.x, na.rm = TRUE)) / sd(.x, na.rm = TRUE)) %>%
  filter(!is.na(BMI) & !is.na(ses1)) %>%
  dplyr::select(ID, gender, age, ethnic, BMI, ses, everything()) %>%
  dplyr::select(-c(ses1:ses3))

glimpse(data_all_processing2)
```

```
## Rows: 791
## Columns: 28
## $ ID       <fct> 666, 667, 668, 669, 670, 671, 672, 673, 674, 675, 676, 677...
## $ gender   <fct> Female, Female, Male, Female, Male, Female, Female, Female...
## $ age      <dbl> -0.4193493, 0.1601681, -0.9988666, 0.1601681, 0.1601681, -...
## $ ethnic   <fct> European ancestry/Caucasian, European ancestry/Caucasian, ...
## $ BMI      <dbl> -0.8101798, -0.4969946, -0.4382901, 0.6101836, -0.6687940,...
## $ ses      <dbl> 0.3447240, -1.3711877, 0.0995938, -2.1065784, 0.0995938, -...
## $ ndrink   <dbl> -0.80135612, -0.80135612, 2.67107343, 0.04044498, 3.167134...
## $ nfruit   <dbl> -0.45884781, 0.06000240, 0.19969284, -0.49875937, -0.89787...
## $ nchips   <dbl> -0.931505013, -0.931505013, 0.474279869, 0.062484298, 0.40...
## $ nveg     <dbl> 0.33540285, 0.16679011, 0.45254434, -0.39229423, -0.347922...
## $ nsweets  <dbl> 0.40187732, 0.23694641, -0.29590728, -0.02948043, -0.15635...
## $ nsoftdrk <dbl> 0.31735839, -0.75171845, -0.12809029, 0.60892480, 0.317358...
## $ rundown  <dbl> -0.61911881, 0.07899834, 0.61313913, -0.37558957, 2.416879...
## $ cold     <dbl> -0.91122300, 0.81306347, 0.27422395, 0.81306347, -0.741873...
## $ hangover <dbl> -0.60824943, -0.60824943, 1.25858684, 0.24031251, 6.059022...
## $ tired    <dbl> 2.22729891, -0.87851479, 0.86074088, -0.27460657, 2.417098...
## $ concent  <dbl> 3.29678178, 0.51340015, 1.83060611, -0.44706252, 2.8655536...
## $ refresh  <dbl> -1.06274887, -0.51363008, 0.66602855, -0.11599234, -0.4378...
## $ sleep    <dbl> -0.90853614, 0.38825035, 0.71134800, 0.02925296, 0.9450626...
## $ dstress  <dbl> 1.43085247, -0.16222855, -0.46362226, 1.94752740, 2.614899...
## $ devstres <dbl> 0.001609552, 0.060777413, -0.519067628, 1.027185815, 1.303...
## $ dpac     <dbl> -0.70985755, 0.29619133, 0.72833505, -0.95513393, -0.75558...
## $ dfruit   <dbl> -0.8216293153, 0.8656936409, 0.4758073656, -0.8543928678, ...
## $ dchips   <dbl> -0.37954435, -0.80986993, -0.50864202, -0.80986993, 0.9114...
## $ dveg     <dbl> -0.59939577, 0.16337039, 0.73212864, -0.59110483, -0.78179...
## $ dsweets  <dbl> 1.32816774, 0.29971853, -1.04898195, -0.52043717, -0.10385...
## $ dsoftdrk <dbl> -0.68852097, -0.68852097, 0.10047174, -0.09079922, 0.06290...
## $ dfs_mean <dbl> -2.127855683, 0.001086728, -0.256920360, -0.966439850, -0....
```

```r
extra_vars <- tibble(`Variable label` = c('genderFemale', 'age',
                                          'ethnicAsian', 'ethnicMaoriDPacificIslander',
                                          'ethnicOther', 'BMI', 'ses'),
                     Name = c('Gender: Female', 'Age', 'Ethnicity: Asian',
                              'Ethnicity: Maori or Pacific Islander',
                              'Ethnicity: Other', 'BMI', 'SES'),
                     `Short name` = c('Female', 'Age', 'Eth: Asian', 'Eth: Maori',
                                      'Eth: Other', 'BMI', 'SES'),
                     Description = NA)
```

```r
vartable_plot <- extra_vars %>%
  bind_rows(vartable) %>%
  filter(!str_detect(`Variable label`, 'dfs') & `Variable label` != 'ID')

data_all_processing2 %>%
  count(gender) %>%
  mutate(prop = n / sum(n))
```

```
## # A tibble: 2 x 3
##   gender     n  prop
##   <fct>  <int> <dbl>
## 1 Male     217 0.274
## 2 Female   574 0.726
```

With the data cleaned, there were 791 participants, one outcome variable (average daily flourishing) and 26 predictor variables, and the ID variable (28 variables in total). Out of the 26 predictor variables, two were categorical (gender, ethnicity), and the rest were continuous. Because the ethnicity variable had four levels (Caucasian, Asian, Maori/Pacific Islander, Other), there were to additional dummy predictors, for a total of 28 predictors.

Next, I sampled the data into a training set (using a seed for reproducibility) and visualized the pairwise correlations between all continuous predictors:

```r
library(ggcorrplot)

unique_ids <- unique(data_all_processing2$ID)
set.seed(123456)
sample_id <- sample(unique_ids, floor(length(unique_ids) * 0.75))

data_training <- data_all_processing2 %>%
  filter(ID %in% sample_id)

corrs <- data_training %>%
  dplyr::select_if(is.numeric) %>%
  dplyr::select(- dfs_mean) %>%
  rownames_to_column() %>%
  gather('Variable label', 'value', -rowname) %>%
  left_join(vartable_plot, by = 'Variable label') %>%
  dplyr::select(rowname, `Short name`, value) %>%
  spread(`Short name`, value) %>%
  dplyr::select(- rowname) %>%
  cor()

ggcorrplot(corrs)
```

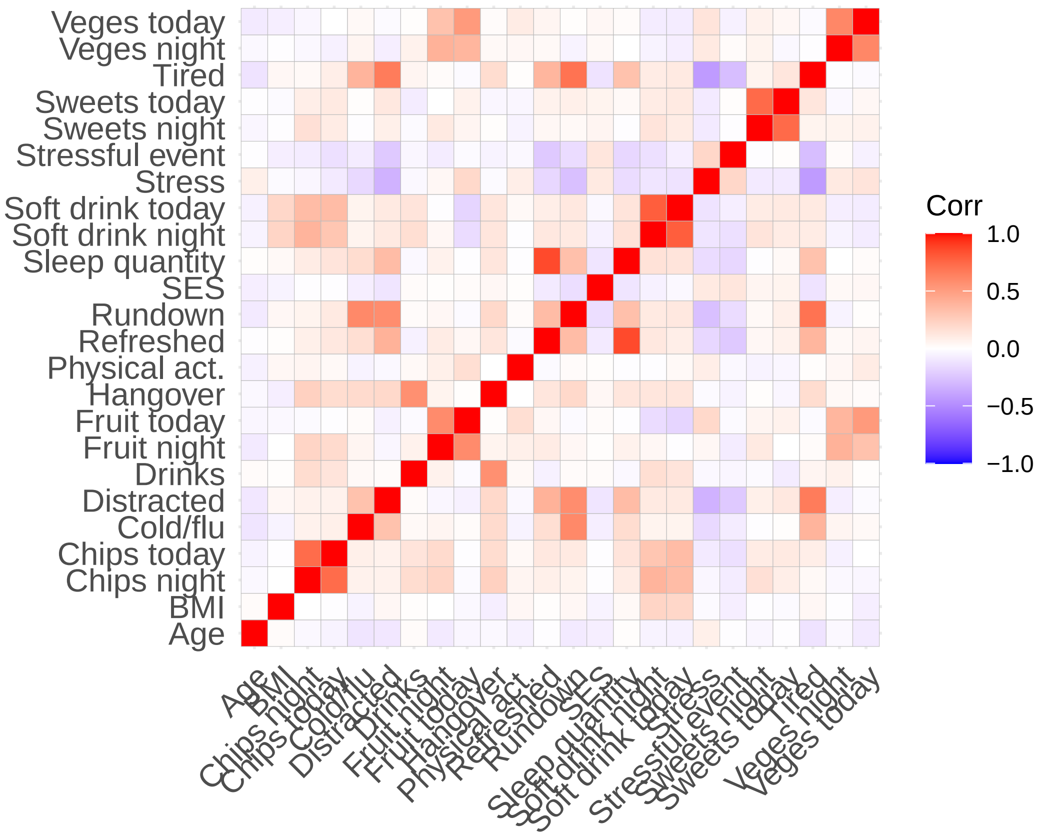

Figure 3: Pairwise correlations between continuous variables

Modeling: Predictive projection

I proceeded with modeling. First, I fitted a Bayesian multiple regression, predicting average daily flourishing from all other variables (excluding for ID). As the values for predictors and the response were standardized, their values for most (95%) observations could be expected to lie between -1.96 and 1.96. It would be very surprising to see any parameters lie outside of the 0 ± 2 range, and thus I put a weakly informative normal(0, 1) priors on the predictor slopes and intercept. Similarly, I put a weakly informative half-normal (0, 1) prior on the model standard deviation (sigma). As for sampling, I ran 4 chains with 4000 iterations each, and set a seed for replicability.

```
library(brms)
library(bayesplot)
library(projpred)
library(loo)
library(cowplot)

reference_fit <- brm(dfs_mean ~ . - ID, data = data_training,
                prior = c(prior(normal(0, 1), class = 'b'),
                          prior(normal(0, 1), class = 'Intercept'),
                          prior(normal(0, 1), class = 'sigma')),
                iter = 4000,
                seed = 123456)

color_scheme_set(scheme = 'gray')
```

```r
summary(reference_fit)
```

```
##  Family: gaussian
##   Links: mu = identity; sigma = identity
## Formula: dfs_mean ~ (ID + gender + age + ethnic + BMI + ses + ndrink + nfruit + nchips + nveg + nswe
##    Data: data_training (Number of observations: 593)
## Samples: 4 chains, each with iter = 4000; warmup = 2000; thin = 1;
##          total post-warmup samples = 8000
##
## Population-Level Effects:
##                              Estimate Est.Error l-95% CI u-95% CI Rhat Bulk_ESS
## Intercept                       -0.11      0.07    -0.25     0.04 1.00    15912
## genderFemale                     0.21      0.09     0.04     0.37 1.00    17014
## age                             -0.04      0.04    -0.11     0.03 1.00    16160
## ethnicAsian                     -0.23      0.13    -0.48     0.02 1.00    12214
## ethnicMaoriDPacificIslander      0.21      0.16    -0.10     0.52 1.00    15684
## ethnicOther                     -0.08      0.13    -0.35     0.18 1.00    16094
## BMI                             -0.08      0.04    -0.14    -0.00 1.00    16908
## ses                              0.03      0.04    -0.04     0.10 1.00    14629
## ndrink                           0.04      0.05    -0.05     0.14 1.00    11086
## nfruit                          -0.02      0.05    -0.12     0.08 1.00     9987
## nchips                          -0.01      0.05    -0.11     0.10 1.00    10638
## nveg                             0.01      0.05    -0.08     0.11 1.00    11327
## nsweets                          0.15      0.05     0.05     0.25 1.00     9862
## nsoftdrk                        -0.15      0.06    -0.27    -0.03 1.00    10149
## rundown                         -0.01      0.06    -0.12     0.11 1.00    10241
## cold                             0.02      0.04    -0.06     0.11 1.00    12030
## hangover                        -0.06      0.05    -0.15     0.04 1.00    11505
## tired                            0.14      0.06     0.03     0.25 1.00    10357
## concent                         -0.28      0.05    -0.38    -0.19 1.00    15339
## refresh                          0.43      0.04     0.35     0.50 1.00    15070
## sleep                           -0.07      0.04    -0.15     0.01 1.00    14537
## dstress                         -0.11      0.07    -0.25     0.03 1.00     8074
## devstres                         0.07      0.07    -0.06     0.21 1.00     8281
## dpac                             0.01      0.03    -0.05     0.08 1.00    17252
## dfruit                           0.09      0.05    -0.01     0.19 1.00     9778
## dchips                          -0.03      0.05    -0.13     0.08 1.00    10448
## dveg                             0.03      0.05    -0.07     0.13 1.00    11222
## dsweets                         -0.15      0.05    -0.26    -0.04 1.00    10021
## dsoftdrk                         0.09      0.06    -0.03     0.20 1.00     9897
##                              Tail_ESS
## Intercept                        6237
## genderFemale                     6270
## age                              5765
## ethnicAsian                      6833
## ethnicMaoriDPacificIslander      6211
## ethnicOther                      6549
## BMI                              6214
## ses                              5769
## ndrink                           6688
## nfruit                           6750
## nchips                           6305
```

```
## nveg                          6547
## nsweets                       6520
## nsoftdrk                      6773
## rundown                       6783
## cold                          6430
## hangover                      6264
## tired                         6746
## concent                       6227
## refresh                       6401
## sleep                         5973
## dstress                       5536
## devstres                      6093
## dpac                          6425
## dfruit                        6370
## dchips                        6548
## dveg                          6282
## dsweets                       6489
## dsoftdrk                      6966
##
## Family Specific Parameters:
##       Estimate Est.Error l-95% CI u-95% CI Rhat Bulk_ESS Tail_ESS
## sigma     0.82      0.02     0.77     0.87 1.00    16267     5616
##
## Samples were drawn using sampling(NUTS). For each parameter, Bulk_ESS
## and Tail_ESS are effective sample size measures, and Rhat is the potential
## scale reduction factor on split chains (at convergence, Rhat = 1).
```

The model converged well, with no divergent transitions, good rhat values (all ~ 1), and large effective sample size (> 5,000) for all parameters.

```
n_nzeuro_females <- data_all_processing2 %>%
  filter(gender == 'Female' & ethnic == 'European ancestry/Caucasian') %>%
  nrow()

prop_nzeuro_females <- round(n_nzeuro_females / nrow(data_all_processing2), 3)

npars <- ncol(as.matrix(reference_fit))

reference_fit_plot <- as.matrix(reference_fit)[, -c(1, npars -1, npars)]

names_to_replace_plot1 <- colnames(reference_fit_plot) %>%
  str_remove(., 'b_') %>%
  tibble(`Variable label` = .) %>%
  left_join(vartable_plot)

colnames(reference_fit_plot) <- names_to_replace_plot1$Name

mcmc_areas(reference_fit_plot) +
  geom_vline(xintercept = 0, linetype = 'dashed', col = 'grey80') +
  scale_x_continuous(limits = c(-0.8, 0.8), breaks = seq(-1, 1, by = 0.25))
```

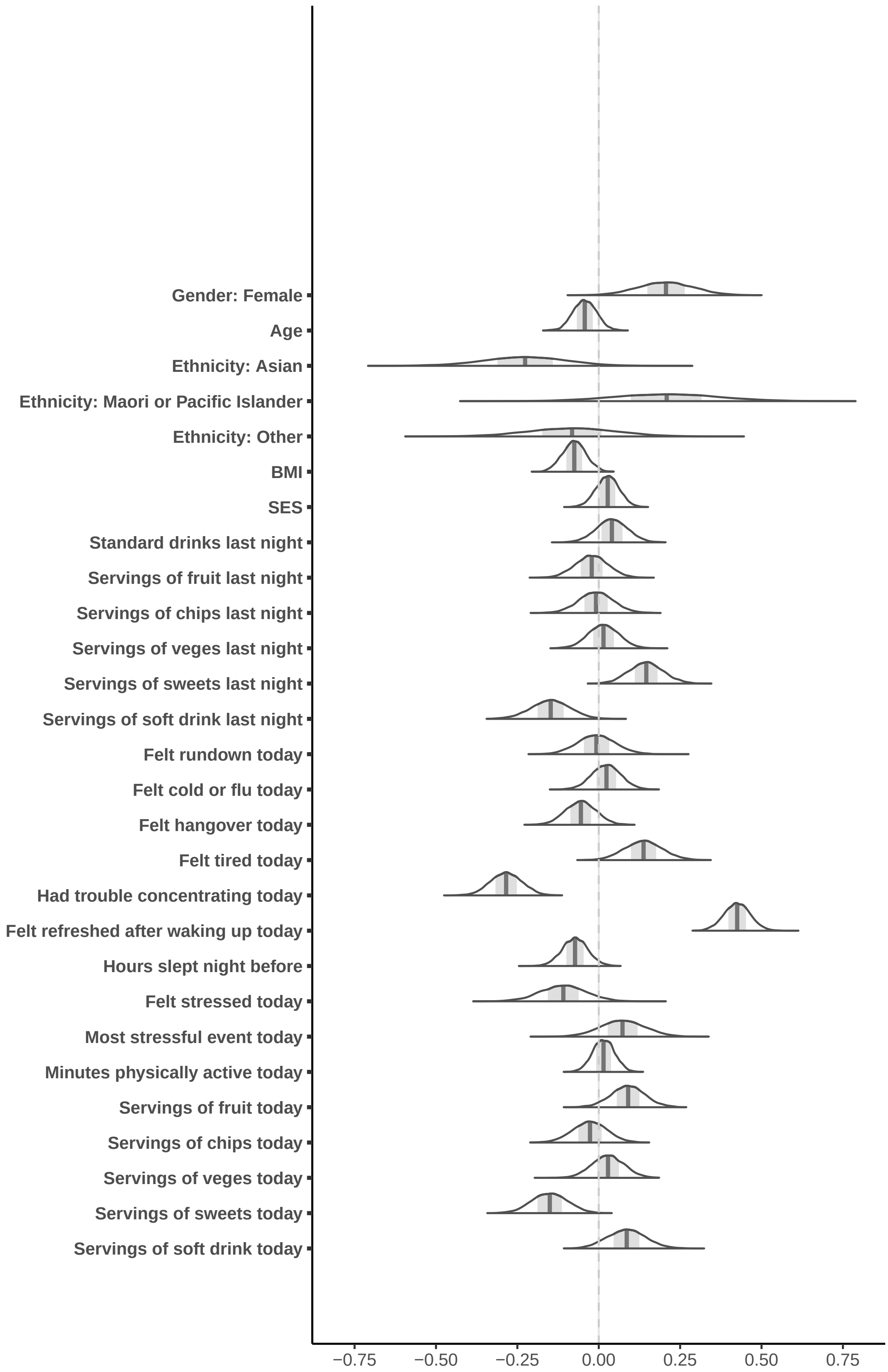

Gender: Female
Age
Ethnicity: Asian
Ethnicity: Maori or Pacific Islander
Ethnicity: Other
BMI
SES
Standard drinks last night
Servings of fruit last night
Servings of chips last night
Servings of veges last night
Servings of sweets last night
Servings of soft drink last night
Felt rundown today
Felt cold or flu today
Felt hangover today
Felt tired today
Had trouble concentrating today
Felt refreshed after waking up today
Hours slept night before
Felt stressed today
Most stressful event today
Minutes physically active today
Servings of fruit today
Servings of chips today
Servings of veges today
Servings of sweets today
Servings of soft drink today
−0.75
−0.50
−0.25
0.00
0.25
0.50
0.75

Figure 4: Marginal distributions of the coefficients in the reference model (28 predictors entered simultaneously)

The marginal posterior credible intervals of the predictor slopes looked did not look unreasonable under model assumptions. Some of the coefficients had an unexpected sign (e.g. felt tired today was positive, suggesting it was predicted *greater* well-being), however, this was likely the result of the predictors mutually controlling for each other. The slopes for gender and the ethnicity predictors had wide credible intervals, but that is not suprising given that the sample was relatively homogenous (0.571 were New Zealand European females).

Next, I proceeded with a visual posterior predictive check and used PSIS-LOO cv to check for influential outliers:

```
pp_check(reference_fit, nsamples = 500, alpha = 0.1) +
  labs(x = 'Average daily flourishing')
```

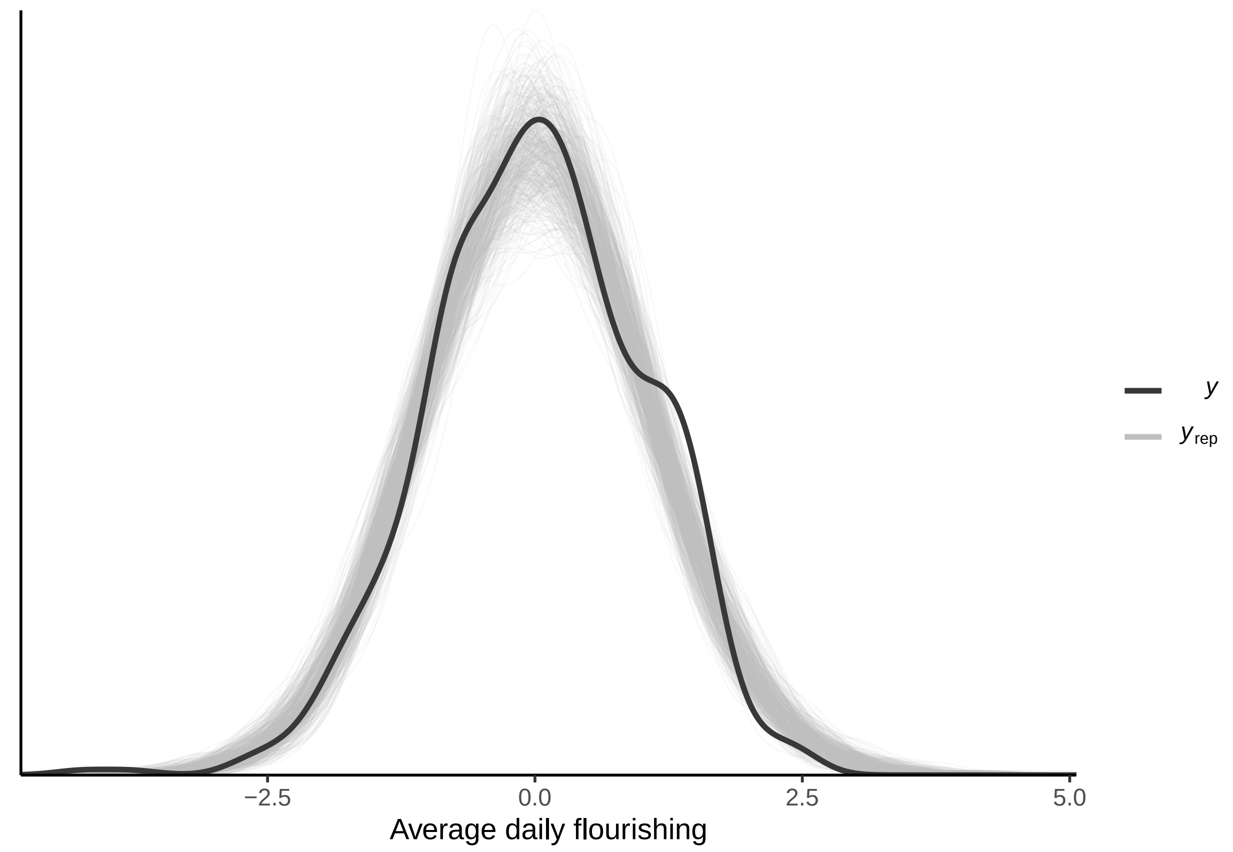

Figure 5: Simulated data of average daily flourishing as draws from the posterior predictive distribution (n = 500) vs. the actual observed distribution of average daily flourishing

```
loo_reference_fit <- loo(reference_fit)

loo_reference_fit
```

```
##
## Computed from 8000 by 593 log-likelihood matrix
##
```

```
##           Estimate   SE
## elpd_loo   -740.0 19.6
## p_loo        30.5  2.4
## looic      1480.0 39.2
## ------
## Monte Carlo SE of elpd_loo is 0.1.
##
## All Pareto k estimates are good (k < 0.5).
## See help('pareto-k-diagnostic') for details.
```

```
plot(loo_reference_fit)
```

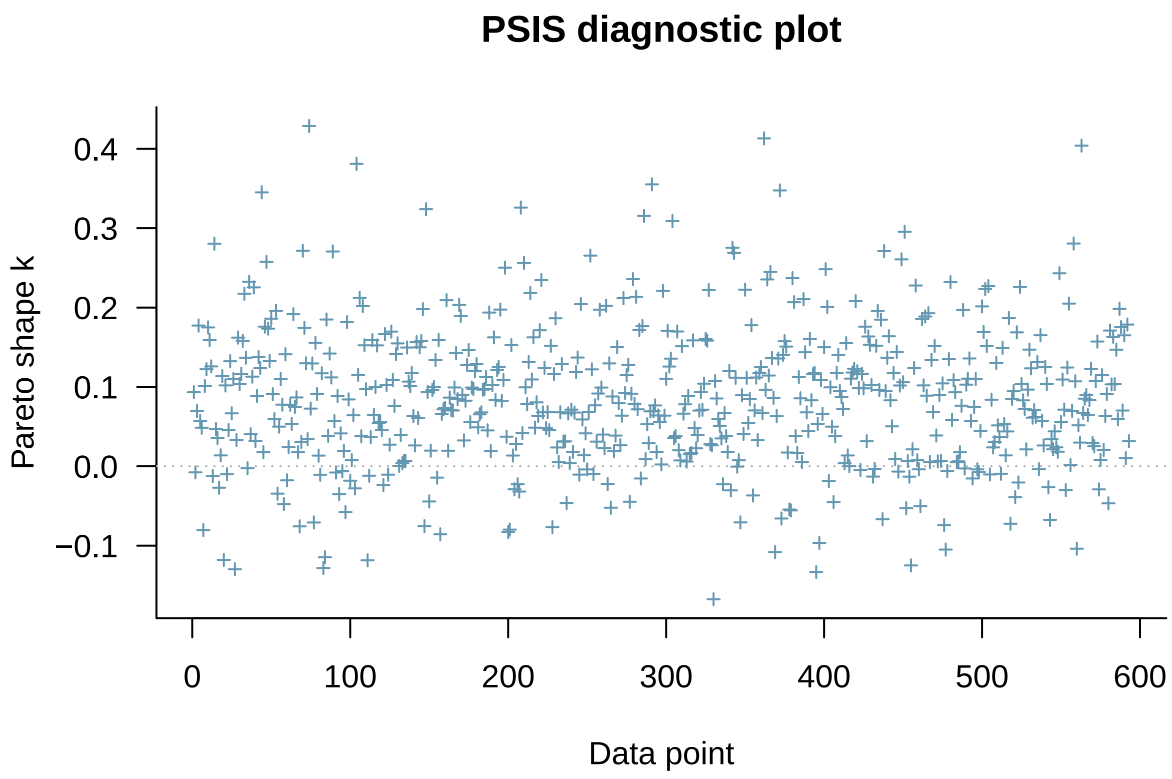

Figure 6: Pareto k values for each observation obtained via PSIS-LOO cross-validation

Using the posterior predictive check, the observed (training) data seemed to match the simulated draws (n = 500) from the posterior predictive distribution fairly well. There were a few outliers with very low average daily flourishing, however Pareto-smoothed importance sampling leave-one-out cross-validation (PSIS-LOO; Vehtari, Gelman, and Gabry 2017) suggested that these observations did not exert overly strong influence on the model fit, as indicated by satisfactory Pareto k values.

Next, I performed predictive proj1ection variable selection (Piironen, Paasiniemi, and Vehtari 2018) using the `projpred` package. The goal of predictive proj1ection is to find the smallest possible submodel (i.e. fewer predictors) similar predictions to those of the reference model.

```
varsel1 <- cv_varsel(reference_fit, seed = 123456)

varsel_stats(varsel1)
```

```r
suggest_size(varsel1)
```

```r
proj1_fig1 <- varsel_plot(varsel1, stats = c('elpd', 'rmse')) +
  theme(axis.title = element_text(size = 10))

proj1 <- project(varsel1, nv = suggest_size(varsel1),
                seed = 123456,
                ns = 2000)

posterior_summary(proj1)
```

```
##                Estimate  Est.Error        Q2.5       Q97.5
## (Intercept)  0.02847727 0.03455064 -0.03779583  0.09461269
## refresh      0.37714455 0.03724514  0.30606324  0.45309301
## concent     -0.21070919 0.03686143 -0.28132101 -0.13783466
## dfruit       0.13219979 0.03453943  0.06537386  0.19986815
## sigma        0.87756124 0.02649976  0.82587684  0.93127359
```

```r
npars2 <- ncol(as.matrix(proj1))
proj1_plot <- as.matrix(proj1)[, -c(1, npars2)]

names_to_replace_plot2 <- colnames(proj1_plot) %>%
  str_remove(., 'b_') %>%
  tibble(`Variable label` = .) %>%
  left_join(vartable_plot) %>%
  mutate(Name = case_when(
    Name == 'Felt refreshed after waking up today' ~ 'Refreshed',
    Name == 'Had trouble concentrating today' ~ 'Distracted',
    Name == 'Servings of fruit today' ~ 'Fruit'
  ))

colnames(proj1_plot) <- names_to_replace_plot2$Name

coef_fig <- mcmc_areas(proj1_plot) +
  geom_vline(xintercept = 0, linetype = 'dashed', col = 'grey60') +
  scale_x_continuous(limits = c(-0.75, 0.75), breaks = seq(-1, 1, by = 0.25)) +
  labs(x = 'Coefficient size (95% CI)') +
  theme(axis.title = element_text(size = 10)) +
  theme(axis.text.y = element_text(angle = 325, size = 8, vjust = 1))

preds_proj1 <- proj_predict(proj1, xnew = data_training, seed_samp = 123456) %>% colMeans()
set.seed(123456)
preds_reference_fit <- predict(reference_fit)[, 1]

cor1 <- cor(preds_reference_fit, preds_proj1)

proj1_fig2 <- tibble(preds_proj1, preds_reference_fit) %>%
  ggplot(aes(preds_proj1, preds_reference_fit)) +
  geom_point(size = 1, col = 'grey60') +
  geom_abline(slope = 1, size = 1, color='black') +
  annotate(geom = 'text', y = 2, x  = -0.5,
           label = paste('italic(r) ==', round(cor1, 3)), parse = TRUE) +
```

```
  scale_x_continuous(limits = c(-2.5, 2.5), breaks = seq(-5, 5, by = 1)) +
  scale_y_continuous(limits = c(-3, 3), breaks = seq(-5, 5, by = 1)) +
  labs(x = 'Submodel predictions',
       y = 'Reference model predictions') +
  theme(axis.title = element_text(size = 10))

plot_grid(proj1_fig1, proj1_fig2, labels = c('A', 'B'), nrow = 1, vjust = 1.1)
```

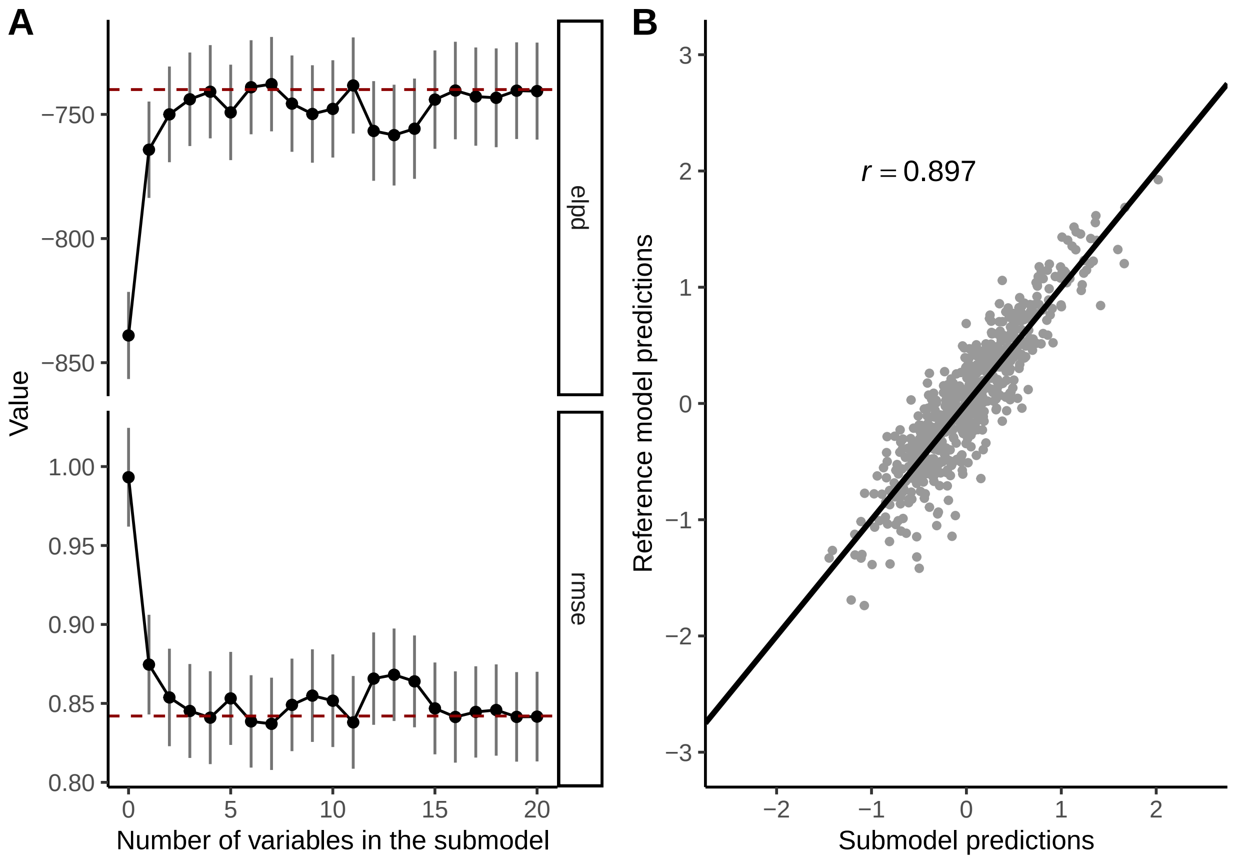


```
ggsave('fig4_projpath.png', height = 100, width = 170, units = 'mm')
```

Figure 7: A) Predictive projection feature selection trajectory, showing the increase in ELPD/decrease in RMSE as more predictors are added. B) Average daily flourishing predicted by the submodel (3 predictors) vs. the average daily flourishing predicted by the reference model (28 predictors; both predicting training data).

The feature selection trajectory shown that the performance of the reference model was reached after 3 predictors, as measured by PSIS-LOO cross-validated expected log-predictive density (ELPD) and root-mean-squared error (RMSE).

```
varsel_stats(varsel1, stats = c('elpd', 'rmse')) %>%
  pander(split.cell = 80, split.table = Inf, justify = 'left',
         caption = 'Feature selection trajectory statistics for
         the first 20 predictors entered into the projection
         submodel')
```

Table 5: Feature selection trajectory statistics for the first 20 predictors entered into the projection submodel

	size	vind	elpd	elpd.se	rmse	rmse.se	pctch
	0	NA	-839	17.65	0.9932	0.03144	NA
**refresh**	1	19	-764.2	19.51	0.8746	0.03175	1
**concent**	2	18	-750	19.38	0.8538	0.03105	1
**dfruit**	3	24	-743.9	18.94	0.8452	0.02992	1
**nsoftdrk**	4	13	-740.9	18.88	0.841	0.02958	1
**dveg**	5	26	-749.2	19.33	0.8532	0.02962	0.9174
**genderFemale**	6	1	-739.1	19.04	0.8386	0.02942	1
**ethnicAsian**	7	3	-737.8	19.11	0.8371	0.02939	0.9966
**BMI**	8	6	-745.7	19.51	0.8491	0.02946	0.8246
**dstress**	9	21	-749.8	19.74	0.855	0.02949	0.7201
**nveg**	10	11	-747.8	19.71	0.8517	0.0295	0.8617
**age**	11	2	-738.4	19.47	0.838	0.02953	0.9966
**tired**	12	17	-756.7	20.18	0.8657	0.02942	0.4553
**sleep**	13	20	-758.3	20.41	0.8681	0.02945	0.8988
**dsweets**	14	27	-755.8	20.3	0.864	0.02925	0.5666
**dchips**	15	25	-744.1	19.92	0.8469	0.02924	0.8229
**ethnicMaori/Pacific Islander**	16	4	-740.4	19.75	0.8414	0.02907	0.7386
**nchips**	17	10	-742.8	19.9	0.8446	0.02907	0.6712
**nsweets**	18	12	-743.3	20.01	0.8458	0.02908	0.9595
**ethnicOther**	19	5	-740.4	19.59	0.8415	0.02852	0.9207
**dpac**	20	23	-740.6	19.65	0.8417	0.02855	0.9224

```
coef_fig
```

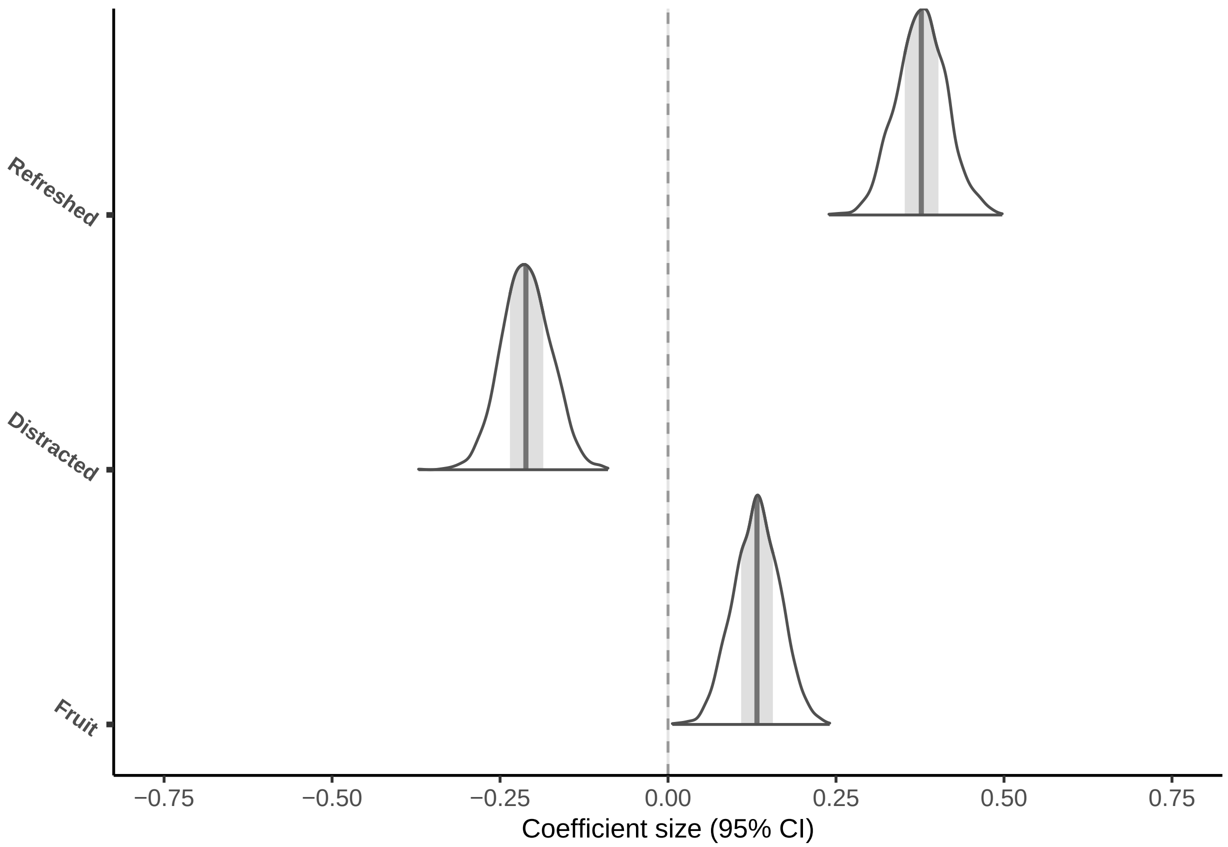

Figure 8: Marginal distributions of the predictors selected in the smallest submodel

We used 1 SE rule (projpred default) to select the optimal submodel size. The 1 SE rule selects the smallest submodel which is expected to perform the same as/outperform the select model with at least 16% probability. Based on the 1 SE rule, the projected submodel contained only the following 3 variables: felt refreshed after waking up today, had trouble concentrating today, and servings of softdrink last night.

Next, I visualized the models' predictions via scatterplots:

```
predvsreal_reference_fit <- tibble(predicted = preds_reference_fit, real = data_training$dfs_mean) %>%
  mutate(error = predicted - real)

predvsreal_proj1 <- tibble(predicted = preds_proj1, real = data_training$dfs_mean) %>%
  mutate(error = predicted - real)

predvsreal_proj1 %>%
  ggplot(aes(predicted, real)) +
  geom_point(size = 1, col = 'grey60') +
  geom_smooth(method = 'lm', col = 'black', se = FALSE) +
  scale_x_continuous(limits = c(-2.5, 2.5), breaks = seq(-5, 5, by = 1)) +
  scale_y_continuous(limits = c(-5, 5), breaks = seq(-5, 5, by = 1)) +
  labs(x = 'Predicted average daily flourishing (proj1ected submodel)',
       y = 'Observed average daily flourishing (training data)')
```

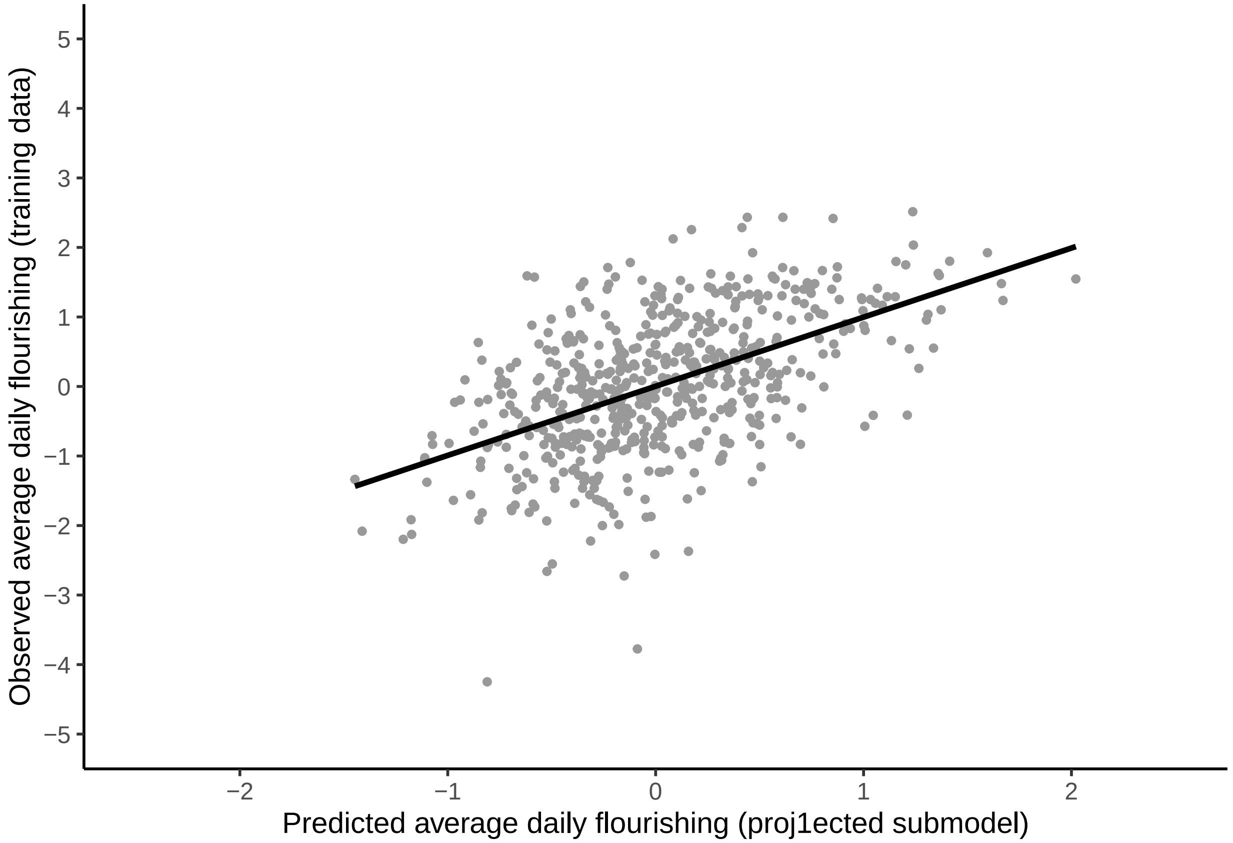

Figure 9: Average daily flourishing predicted by the submodel (3 predictors) vs. the observed average daily flourishing (training data).

```
predvsreal_proj1 %>%
  ggplot(aes(predicted, error)) +
  geom_point(size = 1, col = 'grey60') +
  geom_smooth(col = 'black', se = FALSE) +
  scale_x_continuous(limits = c(-2.5, 2.5)) +
  scale_y_continuous(limits = c(-4.25, 4.25), breaks = seq(-5, 5, by = 1)) +
  labs(x = 'Predicted average daily flourishing (proj1ected submodel)', y = 'Residual')
```

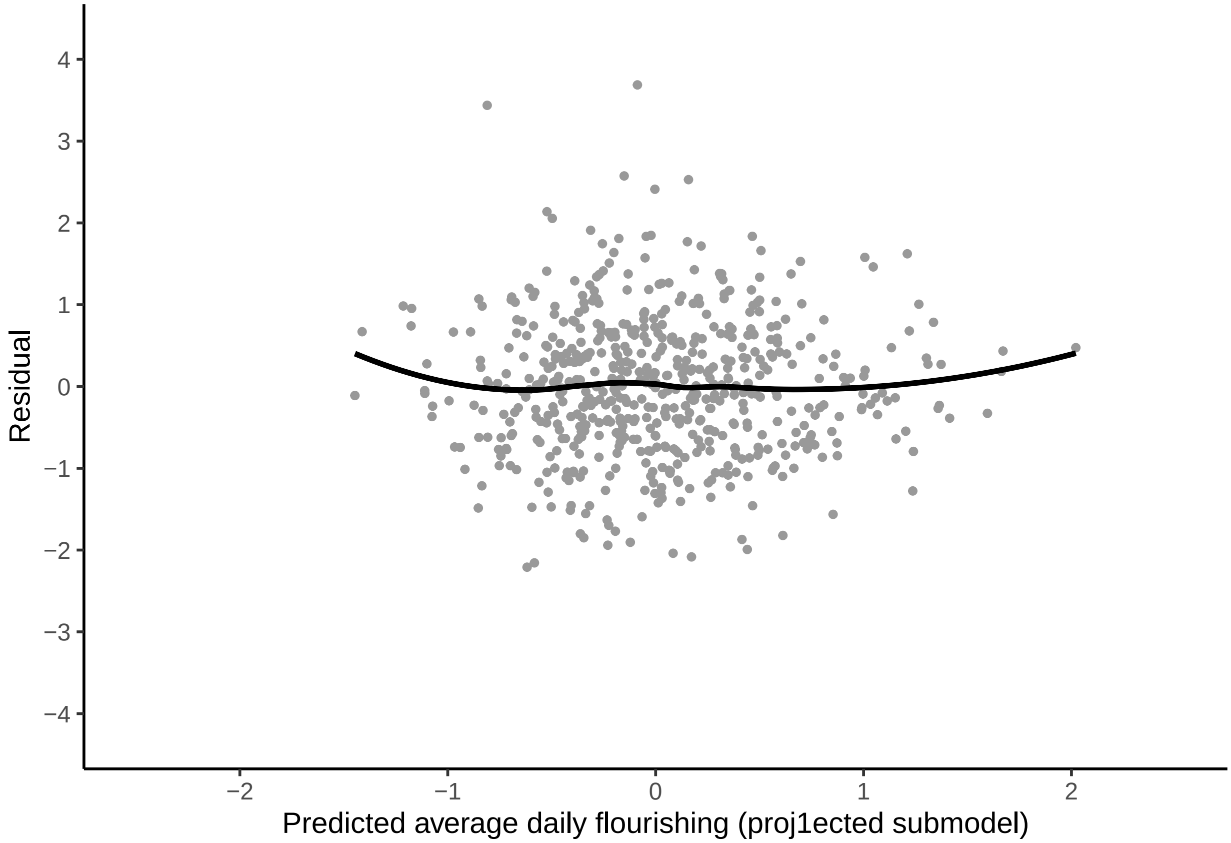

Figure 10: Average daily flourishing predicted by the submodel (3 predictor) vs. the residuals (training data).

The plot of the predicted values vs. residuals did not indicate any gross violation of normality or homoscedasticity, as the residuals seemed to be randomly scattered about zero across the range of predicted values.

```
reference_fit_r2_train_sims <- bayes_R2(reference_fit, summary = FALSE)
reference_fit_r2_train <- bayes_R2(reference_fit)
reference_fit_r2_train_est <- reference_fit_r2_train[1]

reference_fit_r2_train
```

```
##     Estimate  Est.Error      Q2.5     Q97.5
## R2 0.3587729 0.02466028 0.3084195 0.4052853
```

```
get_r2 <- function(object, newdata, y, seed) {

  mupred <- proj_linpred(object, xnew = newdata)

  var_mupred <- apply(mupred, 1, var)
  sigma2 <- as.matrix(object)[, ncol(as.matrix(object))] ^ 2

  r2_sims <- var_mupred / (var_mupred + sigma2)

  r2_sims

}
```

```r
proj1_r2_train_sims <- get_r2(proj1, y = data_training$dfs_mean,
                        newdata = model.matrix(dfs_mean ~ . - ID, data = data_training)[, -1])

proj1_r2_train <- c(mean(proj1_r2_train_sims), quantile(proj1_r2_train_sims, c(0.025, 0.975)))
proj1_r2_train_est <- mean(proj1_r2_train_sims)

proj1_r2_train
```

```
##                2.5%     97.5%
## 0.2674540 0.2130222 0.3231334
```

```r
round(posterior_summary(proj1), 3)
```

```
##             Estimate Est.Error   Q2.5  Q97.5
## (Intercept)    0.028     0.035 -0.038  0.095
## refresh        0.377     0.037  0.306  0.453
## concent       -0.211     0.037 -0.281 -0.138
## dfruit         0.132     0.035  0.065  0.200
## sigma          0.878     0.026  0.826  0.931
```

```r
both_r2 <- tibble(model = c('Reference model', 'Submodel'),
                  estimate = c(reference_fit_r2_train[1], proj1_r2_train[1]),
                  q025 = c(reference_fit_r2_train[3], proj1_r2_train[2]),
                  q975 = c(reference_fit_r2_train[4], proj1_r2_train[3]))

both_r2
```

```
## # A tibble: 2 x 4
##   model           estimate  q025  q975
##   <chr>              <dbl> <dbl> <dbl>
## 1 Reference model    0.359 0.308 0.405
## 2 Submodel           0.267 0.213 0.323
```

```r
both_r2 %>%
  ggplot(aes(model, estimate, ymin = q025, ymax = q975)) +
  geom_pointrange(size = 1) +
  scale_y_continuous(limits = c(0.2, 0.45), breaks = seq(0, 1, by = 0.05)) +
  labs(x = 'Model', y = expression(paste(R^{2}, " (95% CI)"))) +
  guides(col = FALSE)
```

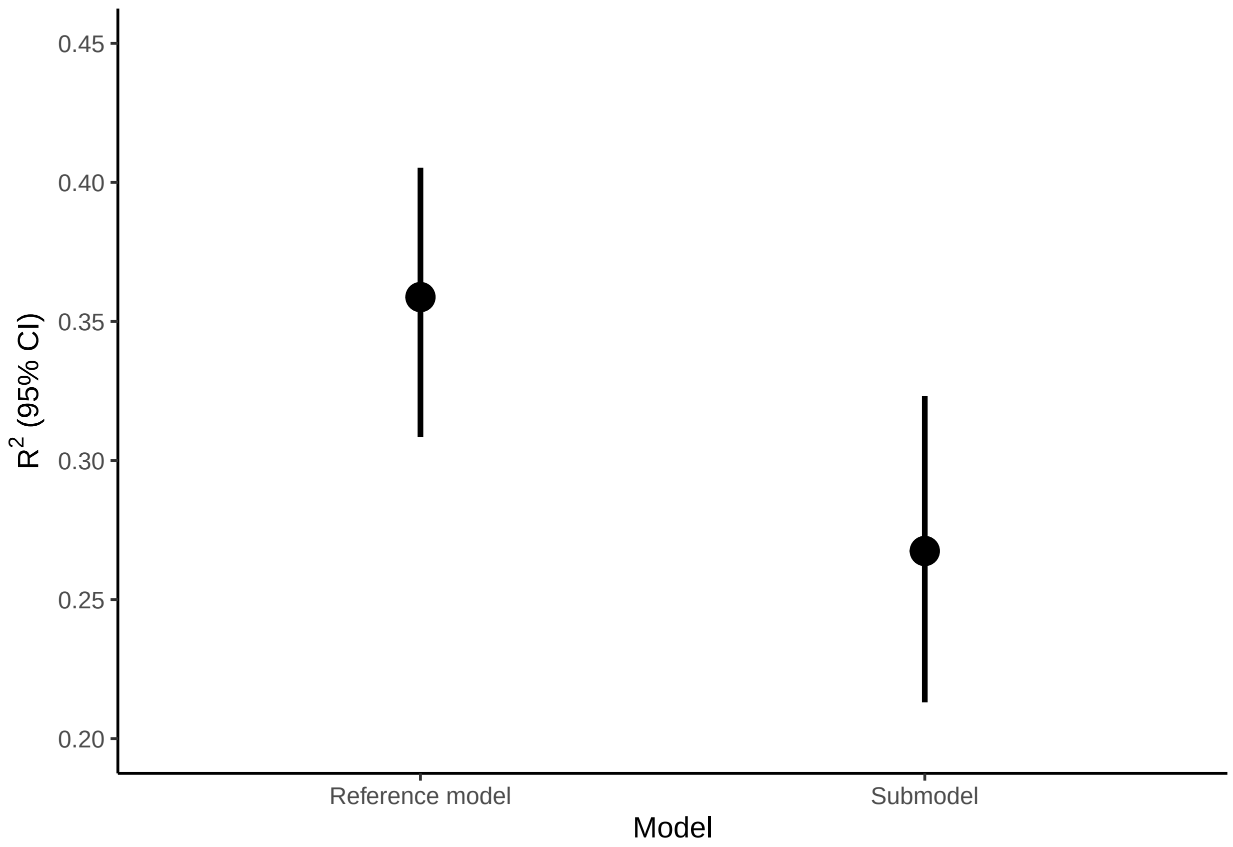


```
both_sims <- tibble(model = c(rep('reference_fit', 8000), rep('proj1', 2000)),
                    sims = c(reference_fit_r2_train_sims, proj1_r2_train_sims))

both_sims %>%
  mutate(model = ifelse(model == 'proj1', 'Projection', 'Reference')) %>%
  ggplot(aes(sims, fill = model)) +
  geom_density(alpha = 0.5, col = NA) +
  scale_fill_grey(start = 0.1, end = 0.4) +
  labs(x = 'R2 (training data)')
```

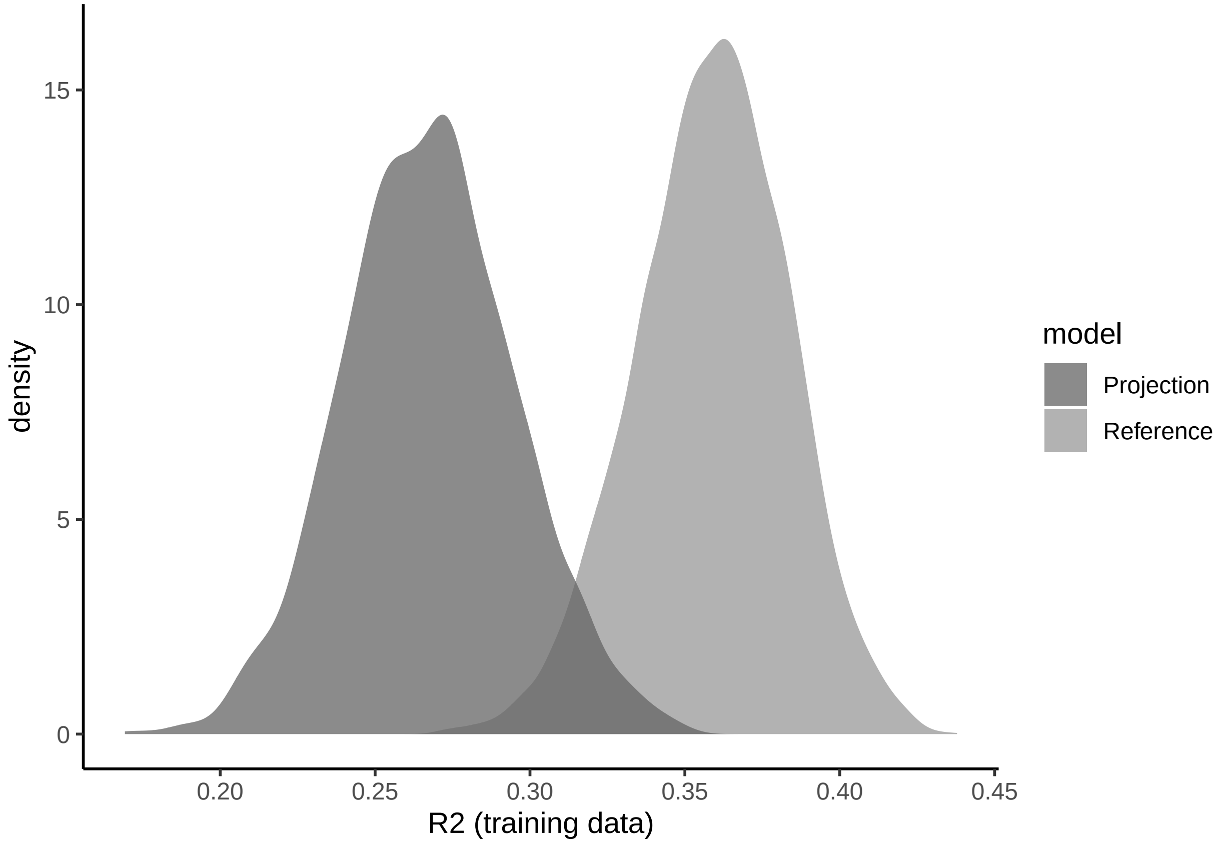

Figure 11: Bayesian R2 of the reference model (28 predictors) vs of the projected submodel (3 predictor slopes; both showing 95% CI)

The projected submodel with 3 predictors slopes performed on the training data similarly to the reference model with all 28 predictors (RMSE reference: 0.799, R2 reference: 0.359, RMSE projection: 0.841, R2 projection: 0.267).

Next, I tested the performance of the projected submodel on the test data:

```
data_test <- data_all_processing2 %>%
  anti_join(data_training)

x_test1 <- model.matrix(dfs_mean ~ . - ID,  data_test) %>%
  as_tibble()

preds_proj1_test <- proj_predict(varsel1, xnew = x_test1, nv = suggest_size(varsel1), seed_samp = 12345

predvsreal_proj1_test <- tibble(predicted = preds_proj1_test, real = data_test$dfs_mean) %>%
  mutate(error = predicted - real)

var_fit_proj1 <- var(predvsreal_proj1_test$predicted)
var_error_proj1 <- var(predvsreal_proj1_test$error)

proj1_r2_test_est <- var_fit_proj1 / (var_fit_proj1 + var_error_proj1)

proj1_r2_test_est
```

```
## [1] 0.2532895
```

```r
predvsreal_test_fig <- predvsreal_proj1_test %>%
  ggplot(aes(predicted, real)) +
  geom_point(size = 1, col = 'grey60') +
  geom_smooth(method = 'lm', col = 'black', se = FALSE) +
  annotate(geom = 'text', x = -1, y = 3,
           label = paste('italic(R) ^ 2 ==', round(proj1_r2_test_est, 3)),
           parse = TRUE) +
  scale_x_continuous(limits = c(-2, 2)) +
  scale_y_continuous(limits = c(-4.25, 4.25), breaks = seq(-5, 5, by = 1)) +
  labs(x = 'Predicted avg. daily flourishing (submodel)', y = 'Observed avg. daily flourishing (test da
  theme(axis.title = element_text(size = 10))

plot_grid(coef_fig, predvsreal_test_fig, labels = c('A', 'B'), nrow = 1, vjust = 1.1)
```

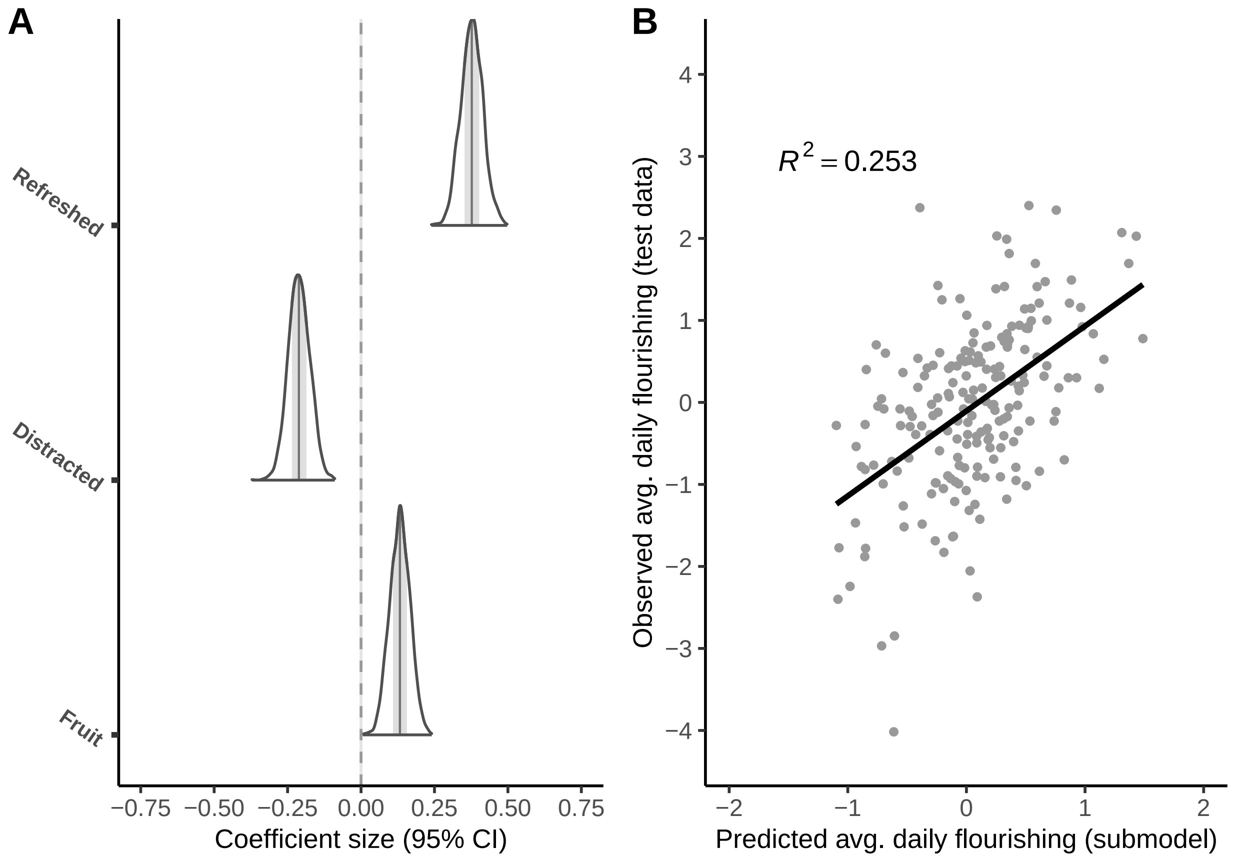


```r
ggsave('fig5_coefpreds.png', height = 100, width = 170, units = 'mm')

round(sqrt(mean(predvsreal_proj1_test$error ^ 2)), 3)
```

```
## [1] 0.883
```

Figure 12: Average daily flourishing predicted by the submodel (3 predictors) vs. the observed average daily flourishing (test data).

The projected submodel had an RMSE of 0.883 and a Bayesian R2 of 0.253 on the test data.

Modeling: LASSO (1 SE), LASSO (min.), multiple regression, stepwise (p-values), stepwise (AIC)

Lastly, to compare the predictive performance and selection of the projection and other variable selection methods, I fitted the following additional models: 1) 1 SE LASSO model, 2) min. cross-validated error LASSO model, 3) single frequentist multiple regression model, 4) stepwise regression using p-values, 5) stepwise regression using Akaike Information Criterion (AIC). I also compared the fit of a projection that matched the reference model (alpha = 1; 50/50 chance that the projected submodel will perform better/worse than the reference model). R does not have a default function for stepwise selection using p-values like SPSS does, so I used code written by Joris Meys which implements SPSS-like stepwise selection in R (see Appendix C).

The methods were compared on test-data R2, RMSE, and the number of predictors selected, using 0.05 alpha level for predictor significance where appropriate (despite the fact that p-values in stepwise regression are invalid because they are not adjusted for the selection process; Flom and Cassell 2007; Smith 2018). Results are summarized in Table 1.

```
library(broom)
library(MASS)
library(glmnet)

x_train2 <- model.matrix(dfs_mean ~ . - ID, data = data_training)
x_test2 <- model.matrix(dfs_mean ~ . - ID, data = data_test)

set.seed(12345)
lasso <- cv.glmnet(x = x_train2, y = data_training$dfs_mean, alpha = 1)

preds_lasso1 <- predict(lasso, newx = x_test2, s = 'lambda.1se')
error_lasso1 <- data_test$dfs_mean - preds_lasso1
var_fit_lasso1 <- var(preds_lasso1)
var_error_lasso1 <- var(error_lasso1)

preds_lasso2 <- predict(lasso, newx = x_test2, s = 'lambda.min')
error_lasso2 <- data_test$dfs_mean - preds_lasso2
var_fit_lasso2 <- var(preds_lasso2)
var_error_lasso2 <- var(error_lasso2)

lasso1_r2_test_est <- var_fit_lasso1 / (var_fit_lasso1 + var_error_lasso1)
lasso1_rmse_test_est <- sqrt(mean(error_lasso1 ^ 2))
lasso2_r2_test_est <- var_fit_lasso2 / (var_fit_lasso2 + var_error_lasso2)
lasso2_rmse_test_est <- sqrt(mean(error_lasso2 ^ 2))

multiple_regression1 <- lm(dfs_mean ~ ., data = data_training[, -1])
multiple_regression2 <- multiple_regression1 %>%
  tidy() %>%
  filter(term != '(Intercept)') %>%
  rename(`Variable label` = 'term') %>%
  left_join(vartable_plot, by = 'Variable label') %>%
  dplyr::select(`Variable label`, Name, estimate,
         std.error, statistic, p.value) %>%
  filter(p.value < 0.05) %>%
  arrange(desc(abs(estimate)))

multiple_regression2 %>%
```

```r
  pander(split.cell = 80, split.table = Inf, justify = 'left',
         'Significant predictors based on a single
         multiple regression')
```

Table 6: Significant predictors based on a single multiple regression

Variable label	Name	estimate	std.error	statistic	p.value
refresh	Felt refreshed after waking up today	0.4262	0.0394	10.82	6.574e-25
concent	Had trouble concentrating today	-0.2846	0.04821	-5.904	6.14e-09
genderFemale	Gender: Female	0.2067	0.08491	2.434	0.01524
dsweets	Servings of sweets today	-0.1515	0.0539	-2.81	0.005119
nsoftdrk	Servings of soft drink last night	-0.1485	0.05926	-2.505	0.01252
nsweets	Servings of sweets last night	0.1466	0.05259	2.787	0.005502
tired	Felt tired today	0.1387	0.05823	2.382	0.01755
BMI	BMI	-0.07563	0.03546	-2.133	0.03339

```r
preds_multi <- predict(multiple_regression1, newdata = data_test)
error_multi <- data_test$dfs_mean - preds_multi
var_fit_multi <- var(preds_multi)
var_error_multi <- var(error_multi)

multi_r2_test_est <- var_fit_multi / (var_fit_multi + var_error_multi)
multi_rmse_test_est <- sqrt(mean(error_multi ^ 2))

step_p_regression1 <- model.select(multiple_regression1)

step_p_regression2 <- step_p_regression1 %>%
  tidy() %>%
  filter(term != '(Intercept)') %>%
  rename(`Variable label` = 'term') %>%
  left_join(vartable_plot, by = 'Variable label') %>%
  dplyr::select(`Variable label`, Name, estimate,
         std.error, statistic, p.value) %>%
  filter(p.value < 0.05) %>%
  arrange(desc(abs(estimate)))

step_p_regression2 %>%
  pander(split.cell = 80, split.table = Inf, justify = 'left',
         'Significant predictors based on stepwise selection
         using p-values')
```

Table 7: Significant predictors based on stepwise selection using p-values

Variable label	Name	estimate	std.error	statistic	p.value
refresh	Felt refreshed after waking up today	0.4184	0.03865	10.83	5.156e-25
concent	Had trouble concentrating today	-0.2908	0.0454	-6.406	3.073e-10
genderFemale	Gender: Female	0.1988	0.08025	2.478	0.01351

Variable label	Name	estimate	std.error	statistic	p.value
dsweets	Servings of sweets today	-0.149	0.05164	-2.885	0.004057
tired	Felt tired today	0.148	0.04799	3.085	0.002133
nsweets	Servings of sweets last night	0.1414	0.05066	2.791	0.005418
dfruit	Servings of fruit today	0.1032	0.03549	2.907	0.003791
nsoftdrk	Servings of soft drink last night	-0.09355	0.03571	-2.619	0.009038

```r
preds_step_p <- predict(step_p_regression1, newdata = data_test)
error_step_p <- data_test$dfs_mean - preds_step_p
var_fit_step_p <- var(preds_step_p)
var_error_step_p <- var(error_step_p)

step_p_r2_test_est <- var_fit_step_p / (var_fit_step_p + var_error_step_p)
step_p_rmse_test_est <- sqrt(mean(error_step_p ^ 2))

step_AIC_regression1 <- stepAIC(multiple_regression1, trace = FALSE)

step_AIC_regression2 <- step_AIC_regression1 %>%
  tidy() %>%
  filter(term != '(Intercept)') %>%
  rename(`Variable label` = 'term') %>%
  left_join(vartable_plot, by = 'Variable label') %>%
  dplyr::select(`Variable label`, Name, estimate,
         std.error, statistic, p.value) %>%
  filter(p.value < 0.05) %>%
  arrange(desc(abs(estimate)))

step_AIC_regression2 %>%
  pander(split.cell = 80, split.table = Inf, justify = 'left',
         caption = 'Significant predictors based on stepwise
         selection using AIC')
```

Table 8: Significant predictors based on stepwise selection using AIC

Variable label	Name	estimate	std.error	statistic	p.value
refresh	Felt refreshed after waking up today	0.4268	0.03856	11.07	5.788e-26
ethnicAsian	Ethnicity: Asian	-0.3104	0.1155	-2.688	0.007401
concent	Had trouble concentrating today	-0.2893	0.04629	-6.248	8.06e-10
genderFemale	Gender: Female	0.209	0.08045	2.598	0.00961
nsoftdrk	Servings of soft drink last night	-0.1549	0.057	-2.717	0.006792
dsweets	Servings of sweets today	-0.154	0.05175	-2.977	0.003035
nsweets	Servings of sweets last night	0.1413	0.05045	2.8	0.005286
tired	Felt tired today	0.1394	0.04883	2.854	0.004469
dfruit	Servings of fruit today	0.09468	0.03595	2.633	0.008679
BMI	BMI	-0.07469	0.03459	-2.159	0.03124

```r
preds_step_AIC <- predict(step_AIC_regression1, newdata = data_test)
error_step_AIC <- data_test$dfs_mean - preds_step_AIC
```

```r
var_fit_step_AIC <- var(preds_step_AIC)
var_error_step_AIC <- var(error_step_AIC)

step_AIC_r2_test_est <- var_fit_step_AIC / (var_fit_step_AIC + var_error_step_AIC)
step_AIC_rmse_test_est <- sqrt(mean(error_step_AIC ^ 2))

preds_reference <- predict(reference_fit, newdata = data_test)[, 1]
error_reference <- data_test$dfs_mean - preds_reference
var_fit_reference <- var(preds_reference)
var_error_reference <- var(error_reference)

reference_r2_test_est <- var_fit_reference / (var_fit_reference + var_error_reference)
reference_rmse_test_est <- sqrt(mean(error_reference ^ 2))

reference_signif <- posterior_summary(reference_fit) %>%
  as_tibble(rownames = 'term') %>%
  filter(sign(Q2.5) == sign(Q97.5)) %>%
  mutate(`Variable label` = str_remove(term, 'b_')) %>%
  left_join(vartable_plot) %>%
  arrange(desc(abs(Estimate))) %>%
  drop_na(Name)

proj1_rmse_test_est <- sqrt(mean((data_test$dfs_mean - preds_proj1_test) ^ 2))

preds_proj2_test <- proj_predict(varsel1, xnew = x_test1,
                                 nv = suggest_size(varsel1, alpha = 1),
                                 seed_samp = 123456) %>% colMeans()

var_fit_proj2 <- var(preds_proj2_test)
error_proj2 <- data_test$dfs_mean - preds_proj2_test
var_error_proj2 <- var(error_proj2)
proj2_r2_test_est <- var_fit_proj2 / (var_fit_proj2 + var_error_proj2)
proj2_rmse_test_est <- sqrt(mean(error_proj2 ^ 2))


R2 <- c(reference_r2_test_est, multi_r2_test_est, proj1_r2_test_est,
         proj2_r2_test_est, step_AIC_r2_test_est, step_p_r2_test_est, lasso1_r2_test_est,
         lasso2_r2_test_est)

RMSE <- c(reference_rmse_test_est, multi_rmse_test_est, proj1_rmse_test_est,
           proj2_rmse_test_est, step_AIC_rmse_test_est, step_p_rmse_test_est, lasso1_rmse_test_est, las

model <- c('Reference Bayesian multiple regression',
            'Multiple regression',
            'Projected submodel (1 SE)',
            'Projected submodel (matched)',
            'Stepwise selection: AIC',
            'Stepwise selection: p-values',
            'LASSO (1 SE)', 'LASSO (min)')

n_predictors <-  c(28, 28, 3, 6, 10, 8, 4, 23)
n_signif_predictors <- c(8, 8, 3, 6, 10, 8, NA, NA)
```

```
selected_predictors <- c(NA, NA,
                 'Felt refreshed after waking up today, Had trouble concentrating today, Servings of fru
                 'Felt refreshed after waking up today, Had trouble concentrating today, Servings of fru
                paste(step_AIC_regression2$Name, collapse = ', '),
                paste(step_p_regression2$Name, collapse = ', '),
                'Felt refreshed after waking up today, Had trouble concentrating today, Servings of fru

comparison <- tibble(model, R2, RMSE, n_predictors, n_signif_predictors, selected_predictors)

comparison %>%
  mutate(R2 = round(R2, 3),
         RMSE = round(RMSE, 3),
         selected_predictors = str_to_sentence(selected_predictors)) %>%
  write_csv('comparison.csv')

comparison %>%
  rename(Model = model, `# of predictors` = n_predictors,
         `# of significant predictors` = n_signif_predictors,
         `Selected predictors` = selected_predictors) %>%
  pander(split.cell = 80, split.table = Inf, justify = 'left',
                      caption = 'R-squared (test data), number of selected
                      and significant predictors, and the names of significant
                      predictors across the variable selection strategies')
```

Table 9: R-squared (test data), number of selected and significant predictors, and the names of significant predictors across the variable selection strategies

Model	R2	RMSE	# of predictors	# of significant predictors	Selected predictors
Reference Bayesian multiple regression	0.3319	0.8563	28	8	NA
Multiple regression	0.3319	0.8575	28	8	NA
Projected submodel (1 SE)	0.2533	0.8834	3	3	Felt refreshed after waking up today, Had trouble concentrating today, Servings of fruit today
Projected submodel (matched)	0.2842	0.864	6	6	Felt refreshed after waking up today, Had trouble concentrating today, Servings of fruit today, Servings of softdrink last night, Servings of vegetables today, Gender: Female
Stepwise selection: AIC	0.3148	0.8723	10	10	Felt refreshed after waking up today, Ethnicity: Asian, Had trouble concentrating today, Gender: Female, Servings of soft drink last night, Servings of sweets today, Servings of sweets last night, Felt tired today, Servings of fruit today, BMI

Model	R2	RMSE	# of predictors	# of significant predictors	Selected predictors
Stepwise selection: p-values	0.2749	0.8713	8	8	Felt refreshed after waking up today, Had trouble concentrating today, Gender: Female, Servings of sweets today, Felt tired today, Servings of sweets last night, Servings of fruit today, Servings of soft drink last night
LASSO (1 SE)	0.1393	0.8971	4	NA	Felt refreshed after waking up today, Had trouble concentrating today, Servings of fruit today, Servings of softdrink last night
LASSO (min)	0.2934	0.8555	23	NA	(not included to save space)

```r
varsel_stats(varsel1)
```

```
##                                size vind      elpd  elpd.se     pctch
##                                   0   NA -839.0344 17.64937        NA
## refresh                           1   19 -764.2303 19.51424 1.0000000
## concent                           2   18 -749.9886 19.37814 1.0000000
## dfruit                            3   24 -743.9156 18.94214 1.0000000
## nsoftdrk                          4   13 -740.8786 18.88338 1.0000000
## dveg                              5   26 -749.1965 19.32960 0.9173693
## genderFemale                      6    1 -739.0698 19.04122 1.0000000
## ethnicAsian                       7    3 -737.8164 19.11386 0.9966273
## BMI                               8    6 -745.6641 19.51298 0.8246206
## dstress                           9   21 -749.8393 19.73504 0.7200675
## nveg                             10   11 -747.7835 19.70831 0.8617201
## age                              11    2 -738.3535 19.47282 0.9966273
## tired                            12   17 -756.6714 20.18372 0.4553120
## sleep                            13   20 -758.3431 20.41227 0.8988196
## dsweets                          14   27 -755.7660 20.30153 0.5666105
## dchips                           15   25 -744.0504 19.92396 0.8229342
## ethnicMaori/Pacific Islander     16    4 -740.3865 19.75231 0.7386172
## nchips                           17   10 -742.8287 19.89680 0.6711636
## nsweets                          18   12 -743.3222 20.01301 0.9595278
## ethnicOther                      19    5 -740.4437 19.58576 0.9207420
## dpac                             20   23 -740.6190 19.64575 0.9224283
```

The reference model, the frequentist multiple regression, and LASSO (min.) had the best predictive performance as measured by test data RMSE (and all included >20 predictors). After that, based on Bayesian R2, the models which had the best predictive accuracy based on R2 were: stepwise selection (AIC), projected submodel (matched), LASSO (min) stepwise (p-values), projected submodel (1 SE), and LASSO (1 SE). Based on RMSE, the models with best predictive accuracy were: reference model, LASSO (min.) the frequentist multiple regression, projected submodel (matched), stepwise (p-values), stepwise (AIC), projected submodel (1 SE), LASSO (1 SE). Apart from 1 SE LASSO, projection selected by far the most parsimonious models, with the 1 SE projection selecting 3 predictors and the matched projection selecting 6 predictors, which is fewer than in the other models. The multiple, stepwise, and LASSO (min.) all resulted in much greater number of selected predictors.

```
predvsreal_all <- tibble(
  model = factor(rep(c('Ref. Bayesian multiple regression',
            'Multiple regression',
            'Projected submodel (1 SE)',
            'Projected submodel (matched)',
            'Stepwise selection: AIC',
            'Stepwise selection: p-values',
            'LASSO (1 SE)', 'LASSO (min.)'), each = nrow(data_test))),
  preds = c(preds_reference, preds_multi, preds_proj1_test, preds_proj2_test,
            preds_step_AIC, preds_step_p, preds_lasso1[, 1], preds_lasso2[, 1]),
  real = rep(data_test$dfs_mean, 8)
)


predvsreal_all <- predvsreal_all %>%
  mutate(model = factor(model, levels = c('Ref. Bayesian multiple regression',
            'Multiple regression',
            'Projected submodel (1 SE)',
            'Projected submodel (matched)',
            'Stepwise selection: AIC',
            'Stepwise selection: p-values',
            'LASSO (1 SE)', 'LASSO (min.)')))

ggplot(predvsreal_all, aes(preds, real)) +
  geom_point(col = 'grey60') +
  geom_smooth(col = 'black', method = 'lm', se = FALSE) +
  scale_x_continuous(limits = c(-2, 2), breaks = seq(-4, 4, by = 1)) +
  scale_y_continuous(limits = c(-4.25, 4.25), breaks = seq(-4, 4, by = 2)) +
  facet_wrap(~ model)
```

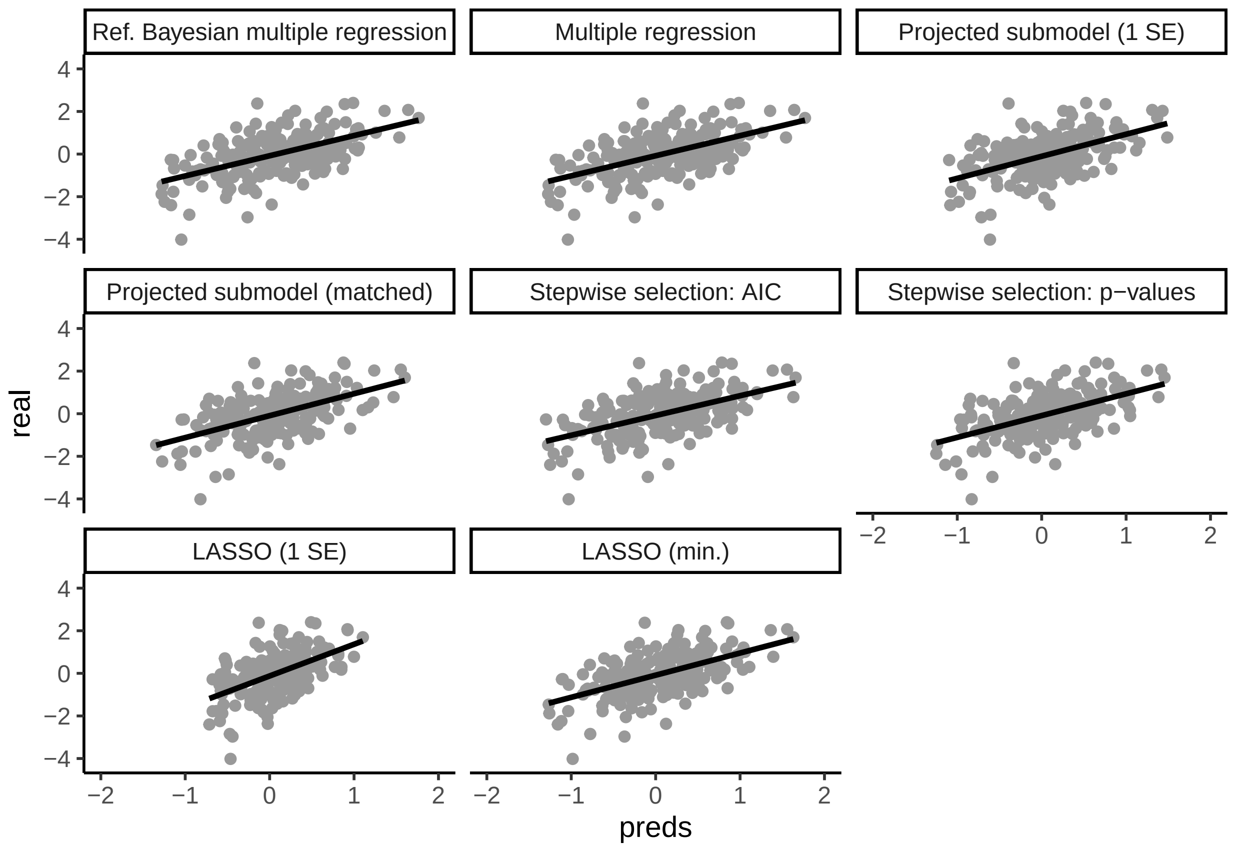

Appendix A: Sensitivity analysis including the first two days of the study

To check that dropping the first two days (to counter initial elevation bias) didn't bias our results, I repeated the analysis with the first two days *included* (and again excluding time spent in nature).

```
data_daily_processing1 <- data_daily_raw %>%
  filter(year %in% c(2013, 2014) & diary == 1 & ndiary >= 7)

data_daily_processing2 <- data_daily_processing1 %>%
  dplyr::select(ID, day, ndrink, nfruit:nsoftdrk, rundown:devstres,
        dpac, dfruit:dsoftdrk, dnat, starts_with('dfs')) %>%
  rename_at(vars(- ID), tolower)

data_daily_processing2 <- data_daily_processing2 %>%
  dplyr::select(- dnat)

data_daily_processing3 <- data_daily_processing2 %>%
  mutate(dfs_mean = dplyr::select(., starts_with('dfs')) %>%
           rowMeans(na.rm = TRUE)) %>%
  dplyr::select(- matches('dfs\\d'))

data_daily_processing4 <- data_daily_processing3 %>%
  group_by(ID) %>%
  summarize_at(vars(- c(day)), ~ mean(.x, na.rm = TRUE))
```

```r
data_all_processing1 <- data_daily_processing4 %>%
  left_join(data_initial_raw, by = 'ID') %>%
  left_join(data_clinic_raw, by = 'ID') %>%
  left_join(data_subjectlist_raw, by = 'ID') %>%
  dplyr::select(ID:dfs_mean, age, gender, ethnic, BMI, ses1:ses3)

data_all_processing2 <- data_all_processing1 %>%
  mutate_at(vars(gender, ethnic), ~ to_factor(.x)) %>%
  mutate(ID = factor(ID),
         ethnic = fct_lump(ethnic, 3),
         ses = dplyr::select(., starts_with('ses')) %>% rowMeans()) %>%
  mutate_if(is.numeric, ~ (.x - mean(.x, na.rm = TRUE)) / sd(.x, na.rm = TRUE)) %>%
  filter(!is.na(BMI) & !is.na(ses1)) %>%
  dplyr::select(ID, gender, age, ethnic, BMI, ses, everything()) %>%
  dplyr::select(-c(ses1:ses3))

unique_ids <- unique(data_all_processing2$ID)
set.seed(123456)
sample_id <- sample(unique_ids, floor(length(unique_ids) * 0.75))

data_training <- data_all_processing2 %>%
  filter(ID %in% sample_id)

reference_fit2 <- brm(dfs_mean ~ . - ID, data = data_training,
                 prior = c(prior(normal(0, 1), class = 'b'),
                           prior(normal(0, 1), class = 'Intercept'),
                           prior(normal(0, 1), class = 'sigma')),
                 iter = 4000,
                 seed = 123456)
```

```
##
## SAMPLING FOR MODEL 'e9e224dfaad0986154b86f4a494c3e47' NOW (CHAIN 1).
## Chain 1:
## Chain 1: Gradient evaluation took 0 seconds
## Chain 1: 1000 transitions using 10 leapfrog steps per transition would take 0 seconds.
## Chain 1: Adjust your expectations accordingly!
## Chain 1:
## Chain 1:
## Chain 1: Iteration:    1 / 4000 [  0%]  (Warmup)
## Chain 1: Iteration:  400 / 4000 [ 10%]  (Warmup)
## Chain 1: Iteration:  800 / 4000 [ 20%]  (Warmup)
## Chain 1: Iteration: 1200 / 4000 [ 30%]  (Warmup)
## Chain 1: Iteration: 1600 / 4000 [ 40%]  (Warmup)
## Chain 1: Iteration: 2000 / 4000 [ 50%]  (Warmup)
## Chain 1: Iteration: 2001 / 4000 [ 50%]  (Sampling)
## Chain 1: Iteration: 2400 / 4000 [ 60%]  (Sampling)
## Chain 1: Iteration: 2800 / 4000 [ 70%]  (Sampling)
## Chain 1: Iteration: 3200 / 4000 [ 80%]  (Sampling)
## Chain 1: Iteration: 3600 / 4000 [ 90%]  (Sampling)
## Chain 1: Iteration: 4000 / 4000 [100%]  (Sampling)
## Chain 1:
## Chain 1:  Elapsed Time: 0.463 seconds (Warm-up)
## Chain 1:                0.461 seconds (Sampling)
```

```
## Chain 1:                0.924 seconds (Total)
## Chain 1:
##
## SAMPLING FOR MODEL 'e9e224dfaad0986154b86f4a494c3e47' NOW (CHAIN 2).
## Chain 2:
## Chain 2: Gradient evaluation took 0 seconds
## Chain 2: 1000 transitions using 10 leapfrog steps per transition would take 0 seconds.
## Chain 2: Adjust your expectations accordingly!
## Chain 2:
## Chain 2:
## Chain 2: Iteration:    1 / 4000 [  0%]  (Warmup)
## Chain 2: Iteration:  400 / 4000 [ 10%]  (Warmup)
## Chain 2: Iteration:  800 / 4000 [ 20%]  (Warmup)
## Chain 2: Iteration: 1200 / 4000 [ 30%]  (Warmup)
## Chain 2: Iteration: 1600 / 4000 [ 40%]  (Warmup)
## Chain 2: Iteration: 2000 / 4000 [ 50%]  (Warmup)
## Chain 2: Iteration: 2001 / 4000 [ 50%]  (Sampling)
## Chain 2: Iteration: 2400 / 4000 [ 60%]  (Sampling)
## Chain 2: Iteration: 2800 / 4000 [ 70%]  (Sampling)
## Chain 2: Iteration: 3200 / 4000 [ 80%]  (Sampling)
## Chain 2: Iteration: 3600 / 4000 [ 90%]  (Sampling)
## Chain 2: Iteration: 4000 / 4000 [100%]  (Sampling)
## Chain 2:
## Chain 2:  Elapsed Time: 0.471 seconds (Warm-up)
## Chain 2:                0.459 seconds (Sampling)
## Chain 2:                0.93 seconds (Total)
## Chain 2:
##
## SAMPLING FOR MODEL 'e9e224dfaad0986154b86f4a494c3e47' NOW (CHAIN 3).
## Chain 3:
## Chain 3: Gradient evaluation took 0 seconds
## Chain 3: 1000 transitions using 10 leapfrog steps per transition would take 0 seconds.
## Chain 3: Adjust your expectations accordingly!
## Chain 3:
## Chain 3:
## Chain 3: Iteration:    1 / 4000 [  0%]  (Warmup)
## Chain 3: Iteration:  400 / 4000 [ 10%]  (Warmup)
## Chain 3: Iteration:  800 / 4000 [ 20%]  (Warmup)
## Chain 3: Iteration: 1200 / 4000 [ 30%]  (Warmup)
## Chain 3: Iteration: 1600 / 4000 [ 40%]  (Warmup)
## Chain 3: Iteration: 2000 / 4000 [ 50%]  (Warmup)
## Chain 3: Iteration: 2001 / 4000 [ 50%]  (Sampling)
## Chain 3: Iteration: 2400 / 4000 [ 60%]  (Sampling)
## Chain 3: Iteration: 2800 / 4000 [ 70%]  (Sampling)
## Chain 3: Iteration: 3200 / 4000 [ 80%]  (Sampling)
## Chain 3: Iteration: 3600 / 4000 [ 90%]  (Sampling)
## Chain 3: Iteration: 4000 / 4000 [100%]  (Sampling)
## Chain 3:
## Chain 3:  Elapsed Time: 0.458 seconds (Warm-up)
## Chain 3:                0.401 seconds (Sampling)
## Chain 3:                0.859 seconds (Total)
## Chain 3:
##
## SAMPLING FOR MODEL 'e9e224dfaad0986154b86f4a494c3e47' NOW (CHAIN 4).
```

```
## Chain 4:
## Chain 4: Gradient evaluation took 0 seconds
## Chain 4: 1000 transitions using 10 leapfrog steps per transition would take 0 seconds.
## Chain 4: Adjust your expectations accordingly!
## Chain 4:
## Chain 4:
## Chain 4: Iteration:    1 / 4000 [  0%]  (Warmup)
## Chain 4: Iteration:  400 / 4000 [ 10%]  (Warmup)
## Chain 4: Iteration:  800 / 4000 [ 20%]  (Warmup)
## Chain 4: Iteration: 1200 / 4000 [ 30%]  (Warmup)
## Chain 4: Iteration: 1600 / 4000 [ 40%]  (Warmup)
## Chain 4: Iteration: 2000 / 4000 [ 50%]  (Warmup)
## Chain 4: Iteration: 2001 / 4000 [ 50%]  (Sampling)
## Chain 4: Iteration: 2400 / 4000 [ 60%]  (Sampling)
## Chain 4: Iteration: 2800 / 4000 [ 70%]  (Sampling)
## Chain 4: Iteration: 3200 / 4000 [ 80%]  (Sampling)
## Chain 4: Iteration: 3600 / 4000 [ 90%]  (Sampling)
## Chain 4: Iteration: 4000 / 4000 [100%]  (Sampling)
## Chain 4:
## Chain 4:  Elapsed Time: 0.437 seconds (Warm-up)
## Chain 4:                0.383 seconds (Sampling)
## Chain 4:                0.82 seconds (Total)
## Chain 4:
```

```
color_scheme_set(scheme = 'gray')

varsel2 <- cv_varsel(reference_fit2, seed = 123456)
```

```
## Computing LOOs...
##   |                                                                              |
```

```
varsel_stats(varsel2)
```

```
##                              size vind      elpd  elpd.se     pctch
##                                 0   NA -841.9427 17.21867        NA
## refresh                         1   19 -761.9484 18.92064 1.0000000
## concent                         2   18 -748.9127 18.67492 1.0000000
## dfruit                          3   24 -742.9870 18.20997 1.0000000
## nsoftdrk                        4   13 -745.8499 18.75724 0.9679595
## dveg                            5   26 -739.5557 18.24024 1.0000000
## genderFemale                    6    1 -739.3841 18.67047 0.9966273
## ethnicAsian                     7    3 -737.7230 18.34282 0.9915683
## BMI                             8    6 -741.1861 18.83617 0.9494098
## dstress                         9   21 -749.0474 19.14692 0.6964587
## nchips                         10   10 -746.8782 19.11447 0.7537943
## nveg                           11   11 -737.1240 18.97448 0.9966273
## age                            12    2 -736.5741 18.91690 1.0000000
## tired                          13   17 -742.0181 19.27945 0.9224283
## ethnicMaori/Pacific Islander   14    4 -741.9793 19.37122 0.9308600
## nsweets                        15   12 -748.2065 19.72448 0.7841484
## cold                           16   15 -753.0759 19.90791 0.4924115
## ses                            17    7 -754.7813 20.04300 0.8330523
```

```
## sleep                             18   20 -751.1210 20.05237 0.7908938
## hangover                          19   16 -745.6036 19.94491 0.9629005
## dsweets                           20   27 -743.6858 19.86158 0.8954469
```

```
varsel_plot(varsel2, stats = c('elpd', 'rmse')) +
  theme(axis.title = element_text(size = 10))
```

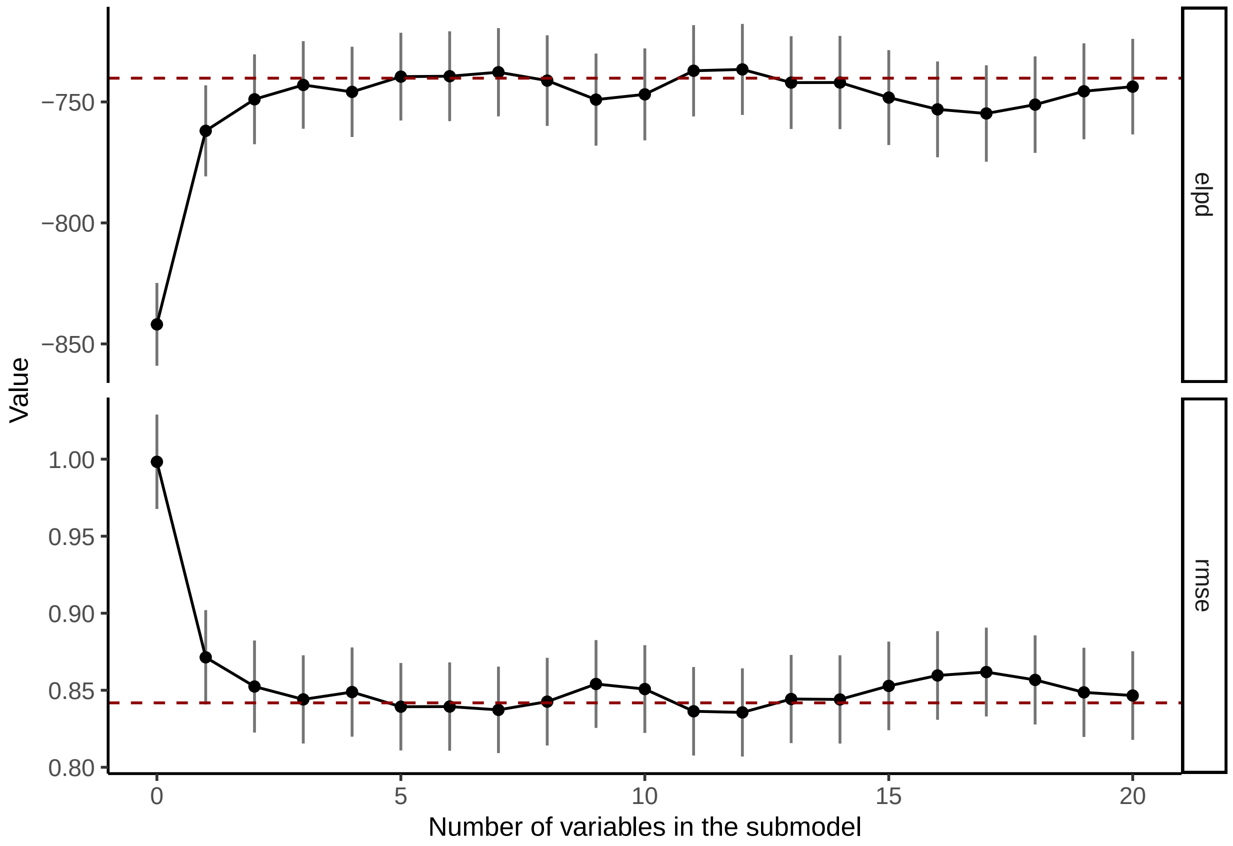

Predictive projection selected the same three variables regardless of whether the first two days of the were dropped or not.

Appendix B: Sensitivity analysis including the time spent in nature variable

To check that dropping the first two days (to counter initial elevation bias) didn't bias our results, I repeated the analysis with the first two days *included*.

```
data_daily_processing1 <- data_daily_raw %>%
  filter(year %in% c(2013, 2014) & diary == 1 & ndiary >= 7 & !(day %in% c(1, 2)))

data_daily_processing2 <- data_daily_processing1 %>%
  dplyr::select(ID, day, ndrink, nfruit:nsoftdrk, rundown:devstres,
        dpac, dfruit:dsoftdrk, dnat, starts_with('dfs')) %>%
  rename_at(vars(- ID), tolower)

data_daily_processing2 <- data_daily_processing2 %>%
  dplyr::select(- dnat)
```

```r
data_daily_processing3 <- data_daily_processing2 %>%
  mutate(dfs_mean = dplyr::select(., starts_with('dfs')) %>%
           rowMeans(na.rm = TRUE)) %>%
  dplyr::select(- matches('dfs\\d'))

data_daily_processing4 <- data_daily_processing3 %>%
  group_by(ID) %>%
  summarize_at(vars(- c(day)), ~ mean(.x, na.rm = TRUE))

data_all_processing1 <- data_daily_processing4 %>%
  left_join(data_initial_raw, by = 'ID') %>%
  left_join(data_clinic_raw, by = 'ID') %>%
  left_join(data_subjectlist_raw, by = 'ID') %>%
  dplyr::select(ID:dfs_mean, age, gender, ethnic, BMI, ses1:ses3)

data_all_processing2 <- data_all_processing1 %>%
  mutate_at(vars(gender, ethnic), ~ to_factor(.x)) %>%
  mutate(ID = factor(ID),
         ethnic = fct_lump(ethnic, 3),
         ses = dplyr::select(., starts_with('ses')) %>% rowMeans()) %>%
  mutate_if(is.numeric, ~ (.x - mean(.x, na.rm = TRUE)) / sd(.x, na.rm = TRUE)) %>%
  filter(!is.na(BMI) & !is.na(ses1)) %>%
  dplyr::select(ID, gender, age, ethnic, BMI, ses, everything()) %>%
  dplyr::select(-c(ses1:ses3))

unique_ids <- unique(data_all_processing2$ID)
set.seed(123456)
sample_id <- sample(unique_ids, floor(length(unique_ids) * 0.75))

data_training <- data_all_processing2 %>%
  filter(ID %in% sample_id)

reference_fit3 <- brm(dfs_mean ~ . - ID, data = data_training,
                 prior = c(prior(normal(0, 1), class = 'b'),
                           prior(normal(0, 1), class = 'Intercept'),
                           prior(normal(0, 1), class = 'sigma')),
                 iter = 4000,
                 seed = 123456)
```

```
##
## SAMPLING FOR MODEL 'e9e224dfaad0986154b86f4a494c3e47' NOW (CHAIN 1).
## Chain 1:
## Chain 1: Gradient evaluation took 0 seconds
## Chain 1: 1000 transitions using 10 leapfrog steps per transition would take 0 seconds.
## Chain 1: Adjust your expectations accordingly!
## Chain 1:
## Chain 1:
## Chain 1: Iteration:    1 / 4000 [  0%]  (Warmup)
## Chain 1: Iteration:  400 / 4000 [ 10%]  (Warmup)
## Chain 1: Iteration:  800 / 4000 [ 20%]  (Warmup)
## Chain 1: Iteration: 1200 / 4000 [ 30%]  (Warmup)
## Chain 1: Iteration: 1600 / 4000 [ 40%]  (Warmup)
## Chain 1: Iteration: 2000 / 4000 [ 50%]  (Warmup)
```

```
## Chain 1: Iteration: 2001 / 4000 [ 50%]  (Sampling)
## Chain 1: Iteration: 2400 / 4000 [ 60%]  (Sampling)
## Chain 1: Iteration: 2800 / 4000 [ 70%]  (Sampling)
## Chain 1: Iteration: 3200 / 4000 [ 80%]  (Sampling)
## Chain 1: Iteration: 3600 / 4000 [ 90%]  (Sampling)
## Chain 1: Iteration: 4000 / 4000 [100%]  (Sampling)
## Chain 1:
## Chain 1:  Elapsed Time: 0.42 seconds (Warm-up)
## Chain 1:                0.404 seconds (Sampling)
## Chain 1:                0.824 seconds (Total)
## Chain 1:
##
## SAMPLING FOR MODEL 'e9e224dfaad0986154b86f4a494c3e47' NOW (CHAIN 2).
## Chain 2:
## Chain 2: Gradient evaluation took 0 seconds
## Chain 2: 1000 transitions using 10 leapfrog steps per transition would take 0 seconds.
## Chain 2: Adjust your expectations accordingly!
## Chain 2:
## Chain 2:
## Chain 2: Iteration:    1 / 4000 [  0%]  (Warmup)
## Chain 2: Iteration:  400 / 4000 [ 10%]  (Warmup)
## Chain 2: Iteration:  800 / 4000 [ 20%]  (Warmup)
## Chain 2: Iteration: 1200 / 4000 [ 30%]  (Warmup)
## Chain 2: Iteration: 1600 / 4000 [ 40%]  (Warmup)
## Chain 2: Iteration: 2000 / 4000 [ 50%]  (Warmup)
## Chain 2: Iteration: 2001 / 4000 [ 50%]  (Sampling)
## Chain 2: Iteration: 2400 / 4000 [ 60%]  (Sampling)
## Chain 2: Iteration: 2800 / 4000 [ 70%]  (Sampling)
## Chain 2: Iteration: 3200 / 4000 [ 80%]  (Sampling)
## Chain 2: Iteration: 3600 / 4000 [ 90%]  (Sampling)
## Chain 2: Iteration: 4000 / 4000 [100%]  (Sampling)
## Chain 2:
## Chain 2:  Elapsed Time: 0.43 seconds (Warm-up)
## Chain 2:                0.397 seconds (Sampling)
## Chain 2:                0.827 seconds (Total)
## Chain 2:
##
## SAMPLING FOR MODEL 'e9e224dfaad0986154b86f4a494c3e47' NOW (CHAIN 3).
## Chain 3:
## Chain 3: Gradient evaluation took 0 seconds
## Chain 3: 1000 transitions using 10 leapfrog steps per transition would take 0 seconds.
## Chain 3: Adjust your expectations accordingly!
## Chain 3:
## Chain 3:
## Chain 3: Iteration:    1 / 4000 [  0%]  (Warmup)
## Chain 3: Iteration:  400 / 4000 [ 10%]  (Warmup)
## Chain 3: Iteration:  800 / 4000 [ 20%]  (Warmup)
## Chain 3: Iteration: 1200 / 4000 [ 30%]  (Warmup)
## Chain 3: Iteration: 1600 / 4000 [ 40%]  (Warmup)
## Chain 3: Iteration: 2000 / 4000 [ 50%]  (Warmup)
## Chain 3: Iteration: 2001 / 4000 [ 50%]  (Sampling)
## Chain 3: Iteration: 2400 / 4000 [ 60%]  (Sampling)
## Chain 3: Iteration: 2800 / 4000 [ 70%]  (Sampling)
## Chain 3: Iteration: 3200 / 4000 [ 80%]  (Sampling)
```

```
## Chain 3: Iteration: 3600 / 4000 [ 90%]  (Sampling)
## Chain 3: Iteration: 4000 / 4000 [100%]  (Sampling)
## Chain 3:
## Chain 3:  Elapsed Time: 0.421 seconds (Warm-up)
## Chain 3:                0.388 seconds (Sampling)
## Chain 3:                0.809 seconds (Total)
## Chain 3:
##
## SAMPLING FOR MODEL 'e9e224dfaad0986154b86f4a494c3e47' NOW (CHAIN 4).
## Chain 4:
## Chain 4: Gradient evaluation took 0 seconds
## Chain 4: 1000 transitions using 10 leapfrog steps per transition would take 0 seconds.
## Chain 4: Adjust your expectations accordingly!
## Chain 4:
## Chain 4:
## Chain 4: Iteration:    1 / 4000 [  0%]  (Warmup)
## Chain 4: Iteration:  400 / 4000 [ 10%]  (Warmup)
## Chain 4: Iteration:  800 / 4000 [ 20%]  (Warmup)
## Chain 4: Iteration: 1200 / 4000 [ 30%]  (Warmup)
## Chain 4: Iteration: 1600 / 4000 [ 40%]  (Warmup)
## Chain 4: Iteration: 2000 / 4000 [ 50%]  (Warmup)
## Chain 4: Iteration: 2001 / 4000 [ 50%]  (Sampling)
## Chain 4: Iteration: 2400 / 4000 [ 60%]  (Sampling)
## Chain 4: Iteration: 2800 / 4000 [ 70%]  (Sampling)
## Chain 4: Iteration: 3200 / 4000 [ 80%]  (Sampling)
## Chain 4: Iteration: 3600 / 4000 [ 90%]  (Sampling)
## Chain 4: Iteration: 4000 / 4000 [100%]  (Sampling)
## Chain 4:
## Chain 4:  Elapsed Time: 0.426 seconds (Warm-up)
## Chain 4:                0.391 seconds (Sampling)
## Chain 4:                0.817 seconds (Total)
## Chain 4:
```

```
color_scheme_set(scheme = 'gray')

varsel3 <- cv_varsel(reference_fit3, seed = 123456)
```

```
## Computing LOOs...
##   |                                                                              |
```

```
varsel_stats(varsel3)
```

```
##                              size vind      elpd  elpd.se     pctch
##                                 0   NA -839.0344 17.64937        NA
## refresh                         1   19 -764.2303 19.51424 1.0000000
## concent                         2   18 -749.9886 19.37814 1.0000000
## dfruit                          3   24 -743.9156 18.94214 1.0000000
## nsoftdrk                        4   13 -740.8786 18.88338 1.0000000
## dveg                            5   26 -749.1965 19.32960 0.9173693
## genderFemale                    6    1 -739.0698 19.04122 1.0000000
## ethnicAsian                     7    3 -737.8164 19.11386 0.9966273
## BMI                             8    6 -745.6641 19.51298 0.8246206
```

```
## dstress                         9   21 -749.8393 19.73504 0.7200675
## nveg                           10   11 -747.7835 19.70831 0.8617201
## age                            11    2 -738.3535 19.47282 0.9966273
## tired                          12   17 -756.6714 20.18372 0.4553120
## sleep                          13   20 -758.3431 20.41227 0.8988196
## dsweets                        14   27 -755.7660 20.30153 0.5666105
## dchips                         15   25 -744.0504 19.92396 0.8229342
## ethnicMaori/Pacific Islander   16    4 -740.3865 19.75231 0.7386172
## nchips                         17   10 -742.8287 19.89680 0.6711636
## nsweets                        18   12 -743.3222 20.01301 0.9595278
## ethnicOther                    19    5 -740.4437 19.58576 0.9207420
## dpac                           20   23 -740.6190 19.64575 0.9224283
```

```r
varsel_plot(varsel3, stats = c('elpd', 'rmse')) +
  theme(axis.title = element_text(size = 10))
```

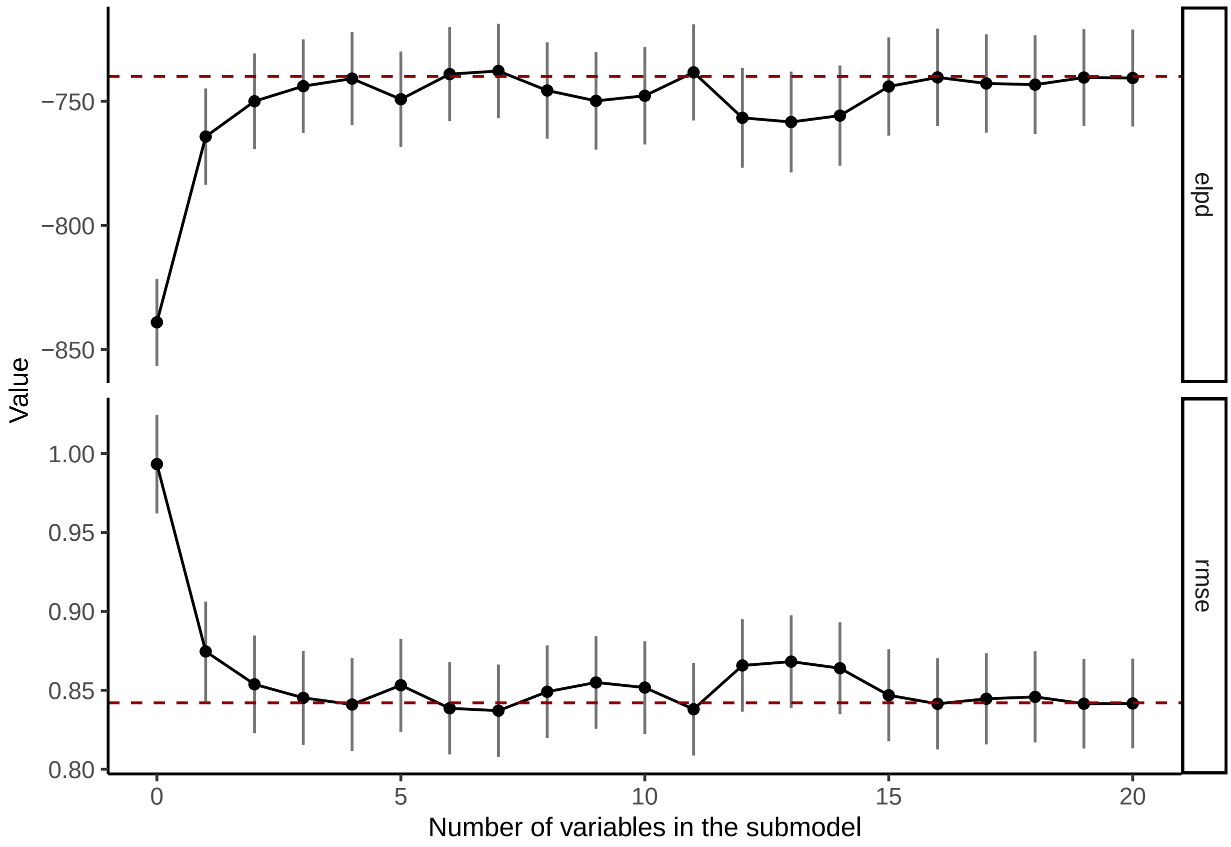

The three variables selected into the predictive projection submodel were the same regardless of whether time spent in nature was included or not.

Appendix C: Joris Meys' code for stepwise selection using p-values in R

```r
######################################
# Automated model selection
# Author      : Joris Meys
# version     : 0.2
# date        : 12/01/09
```

```r
######################################
#CHANGE LOG
# 0.2   : check for empty scopevar vector
######################################

# Function has.interaction checks whether x is part of a term in terms
# terms is a vector with names of terms from a model
has.interaction <- function(x,terms){
  out <- sapply(terms,function(i){
    sum(1-(strsplit(x,":")[[1]] %in% strsplit(i,":")[[1]]))==0
  })
  return(sum(out)>0)
}

# Function Model.select
# model is the lm object of the full model
# keep is a list of model terms to keep in the model at all times
# sig gives the significance for removal of a variable. Can be 0.1 too (see SPSS)
# verbose=T gives the F-tests, dropped var and resulting model after
model.select <- function(model,keep,sig=0.05,verbose=F){
  counter=1
  # check input
  if(!is(model,"lm")) stop(paste(deparse(substitute(model)),"is not an lm object\n"))
  # calculate scope for drop1 function
  terms <- attr(model$terms,"term.labels")
  if(missing(keep)){ # set scopevars to all terms
    scopevars <- terms
  } else{            # select the scopevars if keep is used
    index <- match(keep,terms)
    # check if all is specified correctly
    if(sum(is.na(index))>0){
      novar <- keep[is.na(index)]
      warning(paste(
        c(novar,"cannot be found in the model",
          "\nThese terms are ignored in the model selection."),
        collapse=" "))
      index <- as.vector(na.omit(index))
    }
    scopevars <- terms[-index]
  }

  # Backward model selection :

  while(T){
    # extract the test statistics from drop.
    test <- drop1(model, scope=scopevars,test="F")

    if(verbose){
      cat("-------------STEP ",counter,"-------------\n",
          "The drop statistics : \n")
      print(test)
    }
```

```r
    pval <- test[,dim(test)[2]]

    names(pval) <- rownames(test)
    pval <- sort(pval,decreasing=T)

    if(sum(is.na(pval))>0) stop(paste("Model",
                                  deparse(substitute(model)),"is invalid. Check if all coefficients

    # check if all significant
    if(pval[1]<sig) break # stops the loop if all remaining vars are sign.

    # select var to drop
    i=1
    while(T){
      dropvar <- names(pval)[i]
      check.terms <- terms[-match(dropvar,terms)]
      x <- has.interaction(dropvar,check.terms)
      if(x){i=i+1;next} else {break}
    } # end while(T) drop var

    if(pval[i]<sig) break # stops the loop if var to remove is significant

    if(verbose){
      cat("\n--------\nTerm dropped in step",counter,":",dropvar,"\n--------\n\n")
    }

    #update terms, scopevars and model
    scopevars <- scopevars[-match(dropvar,scopevars)]
    terms <- terms[-match(dropvar,terms)]

    formul <- as.formula(paste(".~.-",dropvar))
    model <- update(model,formul)

    if(length(scopevars)==0) {
      warning("All variables are thrown out of the model.\n",
              "No model could be specified.")
      return()
    }
    counter=counter+1
  } # end while(T) main loop
  return(model)
}
```

References

Diener, Ed, Derrick Wirtz, William Tov, Chu Kim-Prieto, Dong-Won Choi, Shigehiro Oishi, Robert Biswas-Diener, et al. 2009. "New Well-being Measures : Short Scales to Assess Flourishing and Positive and Negative Feelings." *Source: Social Indicators Research Soc Indie Res* 97 (2): 143–56. https://doi.org/10.1007/sll205-009-9493-y.

Flom, Peter L., and David L. Cassell. 2007. "Stopping stepwise: Why stepwise and similar selection methods are bad, and what you should use." In *Northeast Sas User Group (Nesug) Inc 20th Annual Conference:*

11-14th November 2007; Baltimore, Maryland, 1–7.

Piironen, Juho, Markus Paasiniemi, and Aki Vehtari. 2018. "Projective Inference in High-dimensional Problems: Prediction and Feature Selection," no. 2015: 1–42. http://arxiv.org/abs/1810.02406.

Smith, Gary. 2018. "Step away from stepwise." *Journal of Big Data* 5 (1). https://doi.org/10.1186/s40537-018-0143-6.

Vehtari, Aki, Andrew Gelman, and Jonah Gabry. 2017. "Practical Bayesian model evaluation using leave-one-out cross-validation and WAIC." *Statistics and Computing* 27 (5): 1413–32. https://doi.org/10.1007/s11222-016-9696-4.
